# Gene silencing in *Tribolium castaneum* as a tool for the targeted identification of candidate RNAi targets in crop pests

**DOI:** 10.1038/s41598-018-20416-y

**Published:** 2018-02-01

**Authors:** Eileen Knorr, Elane Fishilevich, Linda Tenbusch, Meghan L. F. Frey, Murugesan Rangasamy, Andre Billion, Sarah E. Worden, Premchand Gandra, Kanika Arora, Wendy Lo, Greg Schulenberg, Pablo Valverde-Garcia, Andreas Vilcinskas, Kenneth E. Narva

**Affiliations:** 1 Fraunhofer Institute for Molecular Biology and Applied Ecology, Department of Bioresources, Winchester Str. 2, 35394 Giessen, Germany; 2 Dow AgroSciences, 9330 Zionsville Road, Indianapolis, IN 46268 United States; 3 Institute for Insect Biotechnology, Heinrich-Buff-Ring 26–32, 35392 Giessen, Germany

## Abstract

RNAi shows potential as an agricultural technology for insect control, yet, a relatively low number of robust lethal RNAi targets have been demonstrated to control insects of agricultural interest. In the current study, a selection of lethal RNAi target genes from the iBeetle (*Tribolium castaneum*) screen were used to demonstrate efficacy of orthologous targets in the economically important coleopteran pests *Diabrotica virgifera virgifera* and *Meligethes aeneus*. Transcript orthologs of 50 selected genes were analyzed in *D*. *v*. *virgifera* diet-based RNAi bioassays; 21 of these RNAi targets showed mortality and 36 showed growth inhibition. Low dose injection- and diet-based dsRNA assays in *T*. *castaneum* and *D*. *v*. *virgifera*, respectively, enabled the identification of the four highly potent RNAi target genes: *Rop*, *dre4*, *ncm*, and *RpII140*. Maize was genetically engineered to express dsRNA directed against these prioritized candidate target genes. T_0_ plants expressing *Rop*, *dre4*, or *RpII140* RNA hairpins showed protection from *D*. *v*. *virgifera* larval feeding damage. dsRNA targeting *Rop*, *dre4*, *ncm*, and *RpII140* in *M*. *aeneus* also caused high levels of mortality both by injection and feeding. In summary, high throughput systems for model organisms can be successfully used to identify potent RNA targets for difficult-to-work with agricultural insect pests.

## Introduction

Worldwide, insect pests cause billions of U.S. dollars of yield loss each year. In past decades, the primary solution for insect control was the use of chemical pesticides. Continual application of insecticides has selected for insect resistance to multiple chemistries^1,2^, diminishing grower options to prevent crop damage caused by insects. Beginning in 1996, genetically modified (GM) crops expressing *Bacillus thuringiensis* (Bt) insecticidal proteins, provided a technological improvement in crop protection that significantly reduced reliance on chemical insecticides for important pests in maize, cotton, and more recently soybean^3–5^. Wide adoption of Bt trait technology has generated strong selective pressure that in combination with low fitness costs of resistance^6,7^ has resulted in field-evolved resistance in some pest populations to specific Bt proteins^8,9^ and, thus, created an urgent need for new insect control strategies.

RNA interference (RNAi) is a promising alternative approach for insect control. It is an endogenous cellular process in which double-stranded RNA (dsRNA) directs cleavage of complementary endogenous mRNA resulting in gene silencing^10^. RNAi has been explored as a strategy for pest control by expressing insect-targeted dsRNA in host plants in order to specifically block the expression of essential genes, resulting in insect mortality^11^. One prerequisite for successful plant-delivered insecticidal RNAi is for the target insect to possess an environmental RNAi response^12,13^, triggered by the uptake of dsRNA via feeding by the targeted pest species. Coleopteran insects are known to exhibit a robust environmental RNAi response^14–17^, and in 2007 Baum, *et al*.^15^ successfully engineered the first insect-resistant dsRNA-expressing plant for protection from feeding damage by *Diabrotica virgifera virgifera* LeConte (Coleoptera: Chrysomelidae). This milestone generated a high level of interest in RNAi-based control approaches for *D*. *v*. *virgifera* and other agricultural pests.

One of two pests explored in this study is the western corn rootworm (WCR), *D*. *v*. *virgifera*, which causes annual control and yield loss costs exceeding $US 1 billion in maize (*Zea mays* L.) production in North America^18^. The control of *D*. *v*. *virgifera* is challenged by the development of resistance to traditional insecticides^19^, crop rotation^20^, and GM crops expressing Bt Cry3 proteins, including Cry3Bb, mCry3A and Cry3A.1Ab^21–23^. Moreover, a recent study also observed increased tolerance to Cry34/35Ab1 in *Diabrotica*^24^. To counter resistance in *Diabrotica*, maize technology that combines Bt proteins and an RNAi trait, DvSnf7, is being developed for commercial WCR control^16,25–31^. A field study that evaluated the efficacy and durability of transgenic maize expressing two *Diabrotica*-active Bt traits (Cry3Bb1 and Cry34Ab1/Cry35Ab1 binary toxin) alone or with the DvSnf7 RNAi trait (SmartStax maize and SmartStax PRO maize respectively), estimated that the RNAi trait reduced rootworm emergence by 80–95%^29^. [SmartStax and SmartStax PRO are registered trademarks of Monsanto Technology LLC. SmartStax and SmartStax PRO are multi-event technologies developed by Dow AgroSciences and Monsanto.] Additionally DvSnf7, induces mortality on *D*. *v*. *virgifera* but on a slower time scale than Bts^16^. Modeling that accompanied the field trials indicted a high potential for RNAi to increase the durability of the Bt traits. This also suggests that additional RNAi targets in rootworm that are more highly efficacious may be identified to enable future pyramided insect-protected trait products.

Unlike *D*. *v*. *virgifera*, for which RNAi methodologies have been established, the pollen beetle, *Meligethes aeneus* Fabricius (Coleoptera: Nitidulidae) does not have a validated RNAi response. *M*. *aeneus* is an important pest that causes damage in oilseed rape (*Brassica napus*), an important food, feed, and biodiesel crop in Europe. In the case of *M*. *aeneus*, resistance to pyrethroids has resulted in substantial crop yield losses in Europe^32,33^. This situation has stimulated development of resistance management strategies that incorporate use of neonicotinoid-, organophosphate-, spinosyn-, and oxadiazine-based insecticides. Development of RNAi-based control could provide an additional mode of action to deploy against *M*. *aeneus*.

Despite early progress for RNAi to control Coleoptera^15,17,34–36^, the identification of novel, highly potent RNAi targets in crop pests such as *M*. *aeneus* remains a major challenge. Due to the limited genomic information or lack of laboratory colonies for many crop pests, high-throughput screening in the species of interest may not be practicable. A pre-screen in a model organism was often proposed as a solution^37–39^, but the application to orthologous RNAi targets in non-model pest insects is not well established.

The first insect genome-wide RNAi screen was performed in S2 *Drosophila* cell culture^40^ and explored genes that were essential for cell viability; these cell-based studies have paved a way for large-scale whole-genome RNAi screens in *Drosophila melanogaster* (Diptera: Drosophiladae)^41^. However, to achieve high rate of success when using pre-screens in model insects, closely related species may work better than those more evolutionarily distant. Recently, a pilot RNAi screen (iBeetle) in *Tribolium castaneum* Herbst (Coleoptera: Tenebrionidae) has analyzed the function of 5,000 genes^37,39,42^. Analyses of iBeetle results have identified 100 genes that showed lethality of ≥ 90% both nine days after pupal and eleven days after larval dsRNA injection^37^. Ulrich *et al*.^37^ also selected five *D*. *v*. *virgifera* RNAi target orthologs from Baum *et al*.^15^ for testing in *T*. *castaneum;* these gene targets showed lethally in *T*. *castaneum*, suggesting that RNAi target genes may be leveraged across Coleoptera. Additional examples also demonstrate the success of this approach^43,44^.

With a two-fold goal in mind of discovering highly efficacious RNAi targets for *D*. *v*. *virgifera* and leveraging them to *M*. *aeneus* we used information generated in the pilot RNAi screen of the model organism, *T*. *castaneum*. We identified potent RNAi targets that were validated in *D*. *v*. *virgifera* and *M*. *aeneus* feeding bioassays. Our results indicate that like other Coleoptera, *M*. *aeneus* exhibits and environmental and systemic RNAi response. Moreover, transgenic maize expressing dsRNA directed at these targets provided protection against root feeding damage by *D*. *v*. *virgifera*.

## Results

### *T*. *castaneum* screen reveals potential target genes in *D*. *v*. *virgifera*

**Figure 1:**
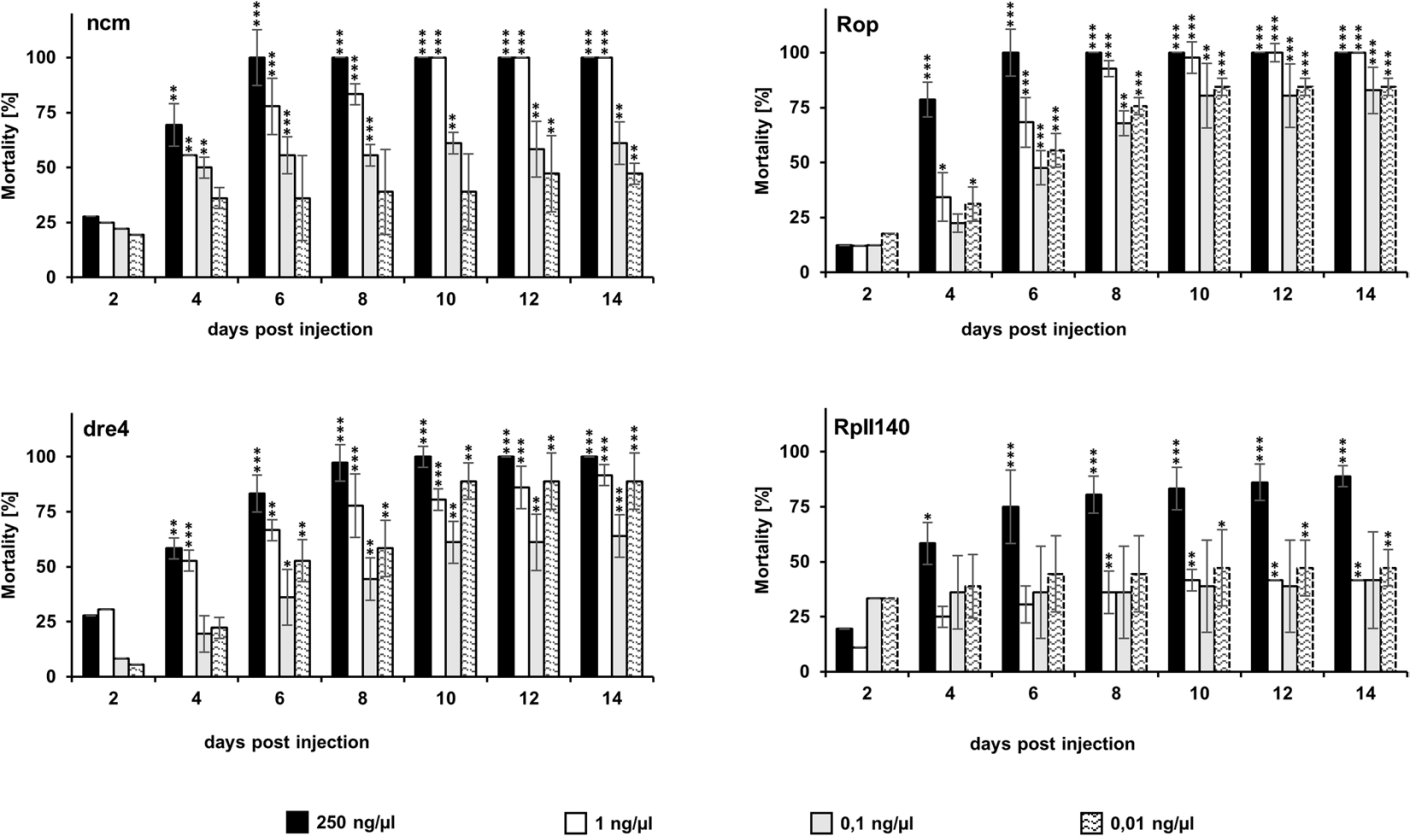
Dose response assay identifies highly active RNAi targets at low dsRNA concentration in *T*. *castaneum*. Injection of 150 nl of gene-specific dsRNA [250 ng/µl] into *T*. *castaneum* larvae. Mortality rates were checked every two days for 14 days. The data represent three biological replicates (n = 10 for each replicate). Raw data is in Supplementary Table 3. The results were analyzed with analysis of variance (ANOVA) (Supplementary Table 4).

We verified the lethality and dose response of the 50 genes selected from the iBeetle database by injecting a range of dsRNA concentrations (250 ng/μl, 1 ng/μl, 0.1 ng/μl and 0.01 ng/μl) into *T*. *castaneum* larvae (Table 1 and Supplementary Tables 1 and 3). Injection of 250 ng/μl dsRNA caused mortality rates of over 90% in over 75% of the tested target genes (Table 1). Dose responses over time for highly-lethal gene targets *ncm*, *Rop*, *dre4*, and *RpII140* injected into *T*. *castaneum* larvae appear in Fig. 1. These targets showed significant mortality at doses down to 0.01 ng/μl within 14 days of dsRNA application, except *RpII140* which showed a significant reduction in the survival at a doses of 1 ng/µl dsRNA.

**Table 1:** Mortality (mean and standard error % mortality in three replicates of bioassays) of 50 dsRNA targets in *T*. *castaneum (T*. *c*.*) and D*. *v*. *virgifera* (*D*. *v*. *v*.). Selected dsRNAs are those that produced the significant mortality of *D*. *v*. *virgifera* (marked with an asterisk). *T*. *castaneum* gene names, and when available, NCBI RefSeq and beetle base IDs are presented. *T*. *castaneum* were injected with 150 nl of 250 ng/μl dsRNA and assessed 14 days post injection. *D*. *v*. *virgifera* were fed dsRNA in diet overlay bioassays for 9 days. Additional bioassay data for *D*. *v*. *virgifera* are included in Supplementary Table 1; raw *D*. *v*. *virgifera* bioassay data are in Supplementary Table 5.

	Gene Name	NCBI ID	beetle base ID	*T*. *c*. % mortality ± SEM	*D*. *v*. *v*. dsRNA	*D*. *v*. *v*.% mortality ± SEM
1	*Arp1*	XM_964734.2	TC030579	100 ± 0	Arp1-1	40.01 ± 4.96*
2	*l(3)72Ab*	XM_965461.1	TC031972	97.22 ± 0	Brr2-1	54.90 ± 9.81*
3	*cas*	XM_962930.1	TC015935	88.89 ± 0.8	Cas-1	27.06 ± 3.36
4	*Cchl*	XM_961483.2	TC011725	100 ± 0	CchI-1	9.22 ± 1.47
5	*Cdc27*	XM_964716.1	TC006902	100 ± 0	CchI-3	41.57 ± 6.12*
6	*Cdc6*	XM_001813172.1	TC013003	100 ± 0	Cdc6-2	29.41 ± 3.39
7	*CG12104*	XM_969426.1	TC012723	63.89 ± 2.12	CG12104-1	29.41 ± 11.00
8	*CG1703*	XM_966469.1	TC004420	77.78 ± 1.6	CG1703-1	7.63 ± 2.90
9	*CG2063*	XM_961444.1	TC003747	97.44 ± 0.76	CG2063-1	22.16 ± 4.51
10	*CG2909*	XM_965359.1	TC014931	100 ± 0	CG2909-1	15.10 ± 4.87
11	*CG34184*	XM_015981502.1	TC004760	100 ± 0	CG34184-2	82.68 ± 9.73 *
12	*CG6843*	XM_966328.2	TC012381	100 ± 0	CG6843-1	52.08 ± 1.70*
13	*Chc*	XM_962736.1	TC015014	69.44 ± 2.12	Chc-1	30.88 ± 14.48
14	*DCTN1-p150*	XM_967301.2	TC012455	86.11 ± 0.8	GI-1	29.69 ± 5.17
15	*dre4*	XM_967384.1	TC014294	100 ± 0	dre4-1	87.49 ± 30.9*
16	*dUTPase*	XM_968608.1	TC008662	100 ± 0	dUTPase-1	53.99 ± 10.97*
17	*fkh*	NM_001039414.2	TC013245	100 ± 0	fsh-1	49.17 ± 6.45*
18	*gcm2*	XM_970010.2	TC014730	100 ± 0	gcm-1	19.12 ± 4.41
19	*Hsc70–3*	XM_965476.2	TC004425	100 ± 0	Hsc70-3-1	47.06 ± 6.62*
20	*Keap1*	XM_961255.1	TC016270	97.22 ± 0	Kaep1-1	22.29 ± 8.96
21	*Klp61F*	XM_965759.2	TC008263	94.44 ± 0.8	Klp61F-1	51.89 ± 6.09*
22	*mam*	—	TC030619	94.44 ± 0.8	mam-1	10.48 ± 3.73
23	*MED17*	XM_964448.1	TC007640	93.33 ± 0	iB-01233-363-534	8.82 ± 2.94
24	*mtRNApol*	XM_962507.1	TC006188	52.78 ± 1,6	mtRNApol-1	8.50 ± 2.62
25	*NAA15–16*	XM_970509.1	TC008190	100 ± 0	NAT1_1-1	47.06 ± 5.88*
26	*ncm*	XM_001811253.1	TC014785	100 ± 0	ncm-1	83.45 ± 5.62*
27	*Past1*	XM_970194.1	TC033251	47.22 ± 8.0	Past1–1	12.13 ± 0.37
28	*Picot*	XM_961223.2	TC003405	77.78 ± 12.73	Picot-1	14.71 ± 2.94
29	*Pka-R1-PP*	XM_967511.2	TC000040	100 ± 0	iB-00004-193-274	23.53 ± 5.88
30	*Prosα1-PA*	XM_961880.2	TC000258	100 ± 0	iB-00141-266-641	41.67 ± 8.78*
31	*Prosα6-PA*	XM_963762.1	TC000069	100 ± 0	iB-00053-911-273	35.10 ± 11.57
32	*Prosβ5*	XM_965101.1	TC030625	100 ± 0	Prosbeta5-1	44.12 ± 14.71
33	*Rab6*	XM_967360.2	TC001600	83.33 ± 3.21	Rab6-1	20.59 ± 2.94
34	*Rop*	NM_001170684.1	TC011120	100 ± 0	Rop-1	82.80 ± 1.78*
35	*Rpb7*	XM_965220.1	TC014109	100 ± 0	Rpb7-1	76.72 ± 3.07*
36	*RpII140*	XM_969560	TC011771	88.89 ± 0	RpII140-1	97.06 ± 1.70*
37	*RpL6*	XM_968022.2	TC030666	100 ± 0	RpL6-1	29.41 ± 5.88
38	*Rpn11*	XM_961333.2	TC005869	100 ± 0	iB-00950-010-291	23.11 ± 5.46
39	*Rpn12*	XM_966866.2	TC007891	100 ± 0	iB-01280-690-586	38.24 ± 7.78*
40	*Rpn7*	XM_968550.1	TC006375	97.22 ± 0	Rpn7-1	59.20 ± 4.97*
41	*Rpt3*	XM_962883.2	TC007999	100 ± 0	Rpt3-1	46.11 ± 3.49*
42	*Sam-S*	XM_961585.2	TC011082	100 ± 0	iB-01800–344-368	18.38 ± 6.62
43	*Sec*. *6*	XM_965057.2	TC010557	100 ± 0	sec. 6-1	47.06 ± 5.88*
44	*Sec*. *61α*	XM_965057.2	TC010557	100 ± 0	iB-07109-147-640	43.77 ± 3.09*
45	*sif*	XM_001815311.1	TC031145	100 ± 0	sif-2	11.77 ± 3.4
46	*snRNP-U1–70K*	XM_962210.2	TC002933	97.78 ± 0	iB-00472-005-561	29.41 ± 0.00
47	*Spx*	XM_963027.1	TC003731	83.33 ± 0.8	Spx_NEW	20.59 ± 8.83
48	*su(f)*	XM_967794.1	TC015727	91.67 ± 1.6	su-f-1	17.65 ± 5.89
49	*Surf4-PA*	XM_964082.2	TC000161	92.59 ± 2.27	iB-000029-022-670	25.10 ± 1.57
50	*Tango6*	XM_001814799.1	TC014300	88.89 ± 1.6	Tango6–2	18.38 ± 6.62

**Figure 2:**
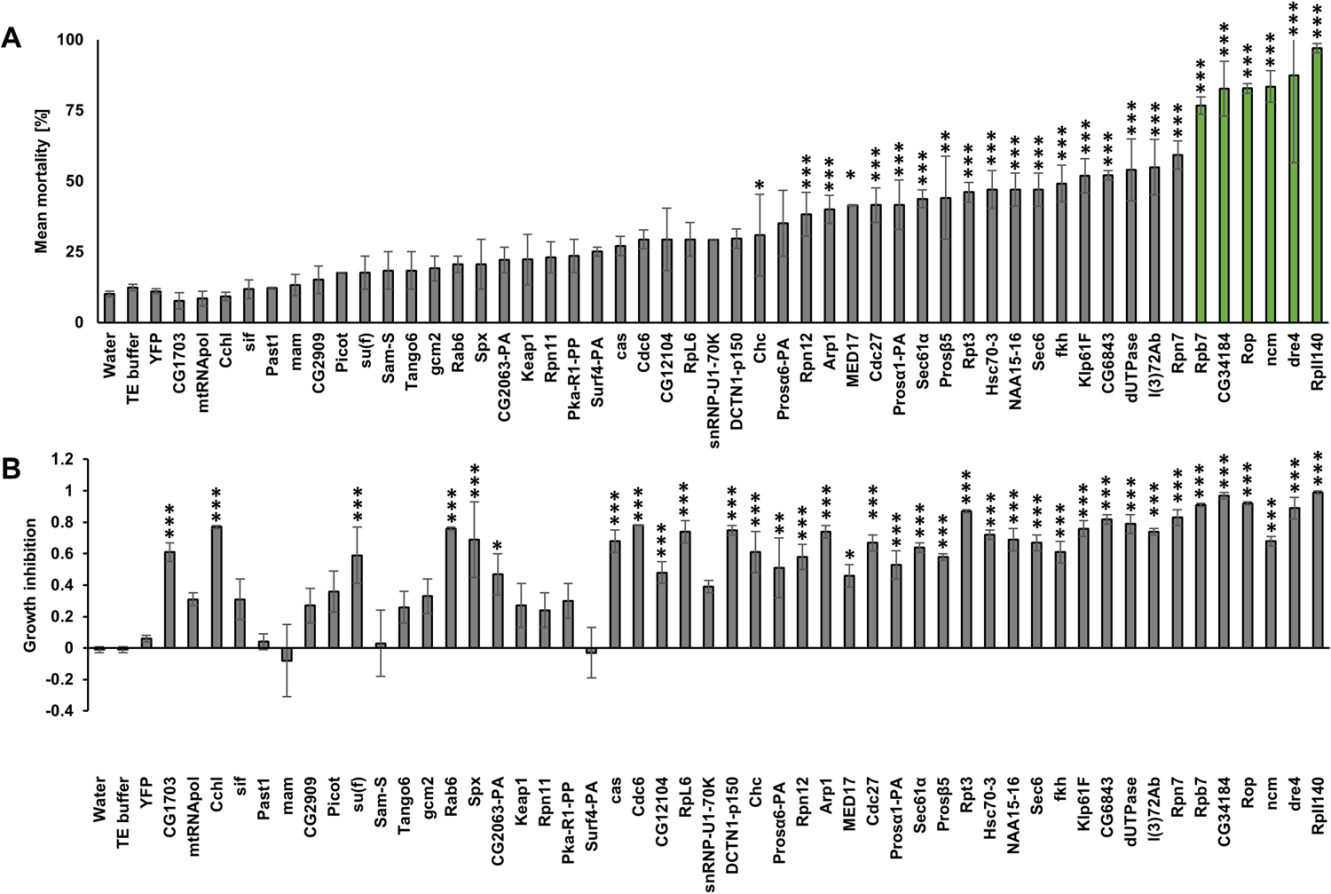
Mortality rates and GI value after dsRNA feeding bioassay revealed new potential *D*. *v*. *virgifera* RNAi targets. Neonate *D*. *v*. *virgifera* larvae were fed on artificial diet that was surface-overlaid with dsRNA at 500 ng/cm^2^. Yellow fluorescent protein gene (YFP) dsRNA, 0.1X TE buffer and water were used as negative controls. (**A**) Mortality (most active treatments are highlighted in green) and (**A**) growth inhibition (GI) were evaluated after nine days. See Supplementary Table 1 for number of replicates and standard deviations. Asterisks show statistical difference (*p < 0.05, **p < 0.01 and ***p < 0.001).

Homologs for all 50 *T*. *castaneum* lethal genes were identified within the 1^st^ instar transcriptome of *D*. *v*. *virgifera* (Supplementary Table 1) via TBLASTN searches using *T*. *castaneum* NCBI RefSeq protein accession IDs in Table 1. The diet feeding bioassays of 50 *D*. *v*. *virgifera* dsRNAs identified 21 dsRNAs with significantly higher percent mortality, compared to YFP dsRNA negative control (*p* < 0.001, marked with an asterisk in Fig. 2A and Supplementary Table 1). Of the 50 dsRNA targets tested in *D*. *v*. *virgifera*, 36 also showed significant Growth Inhibition (Fig. 2B). Larval transcript expression levels were compared to the bioassay outcomes for all 50 *D*. *v*. *virgifera* genes; however, no correlation between gene expression and lethality or growth inhibition were found (Supplementary Fig. 1). In this nine-day bioassay, dsRNAs targeting *RpII140*, *dre4*,*ncm*, *CG34184*, *Rop* and *Rpb7* transcripts, which showed more than 60% mortality (Fig. 2A, highlighted in green), were selected for further characterization.

To further confirm target sensitivity and probe the efficacy of the sub-regions of the selected targets, additional dsRNAs were designed. In most cases, the additional sequences, or versions, were located within the initially-tested active dsRNA region. For example, *ncm-1 v1* and *ncm-1 v2* dsRNAs represent non-overlapping sequences within *ncm-1* dsRNA region (Supplementary Materials, Sequence 4). *Rpb7–1 v1* is a sub-region or *Rpb7–1* dsRNA (Supplementary Materials, Sequence 5); *Rop-2 v3* is a sub-sequence of *Rop-2* (Supplementary Materials, Sequence 1); *dre-1 v1* and *dre-1 v2* are sub-sequences of *dre-1* dsRNA [*dre-1 v2* is also a sub-sequence of *dre-1 v1*] (Supplementary Materials, Sequence 2); and CG34184–2 v1 and CG34184–2 v2 are sub-sequences of CG34184–2 dsRNA (Supplementary Materials, Sequence 6). The additional dsRNA sequences *RpII140 v1* and *RpII140 v2* were outside of the initial *Rp140–1* dsRNA region (Supplementary Materials, Sequence 3). In addition to high-dose diet bioassays, concentration series were tested to estimate LC_50_ and GI_50_ values (concentrations that lead to 50% mortality and growth inhibition, respectively), for both the long dsRNAs and dsRNA sub-regions, or versions. The *CG34184–2* dsRNA sub-sequences selected from the original active sequence *CG34184–2 v1* and *CG34184–2 v2* did not show lethality at high dose (500 ng/cm^2^) in *D*. *v*. *virgifera* diet overlay bioassay, therefore were not considered for LC_50_ determination (Supplementary Table 1). The majority of dsRNAs tested showed LC_50_ values of less than 100 ng/cm^2^ (Table 2); the LC_50_ values ranged from approximately 2.7 ng/cm^2^ to 103.7 ng/cm^2^. Expression constructs for hairpin RNAs (hpRNAs) targeting *dre4*, *ncm*, *RpII140*, and *Rop* were subsequently transformed into maize. Raw data for the *D*. *v*. *virgifera* RNAi dose-response is in Supplementary Table 2.

**Table 2:** LC_50_ and GI_50_ for *dre4*, *ncm*, *RpII140*, and *Rop* in larval *D*. *v*. *virgifera* diet-based bioassay.

Gene Name	LC_50_ (ng/cm^2^)	Range	GI_50_ (ng/cm^2^)	Range
*dre4 v1*	43.7	33.4–57.4	25.6	15.5–42.5
*dre4 v2*	64.8	49.6–85.3	28.0	14.6–53.5
*dre4–1*	3.6	2.4–5.0	2.8	1.2–6.6
*ncm-1*	7.0	4.9–10.0	4.2	1.6–10.9
*ncm-2*	17.3	11.5–26.0	8.2	2.2–30.4
*ncm-1 v2*	2.5	2.2–2.9	18.2	12.8–25.8
*RpII140 v1*	6.7	4.4–9.8	4.3	1.8–10.5
*RpII140 v2*	2.7	1.72–4.01	1.3	0.8–2.3
*RpII 140*	103.7	68.4–167.3	20.5	11.6–36.1
*Rop- 1*	20.4	13.6–30.1	5.9	4.3–8.2
*Rop- 2*	29.7	19.3–45.4	7.1	2.2–23.2
*Rop-2 v3*	25.4	18.5–34.5	10.1	6.3–16.0
*Rpb7–1 v1*	12.5	8.9–17.2	1.2	1.2–2.1

### Plant-mediated RNAi protects maize roots against *D*. *v*. *virgifera*

**Figure 3:**
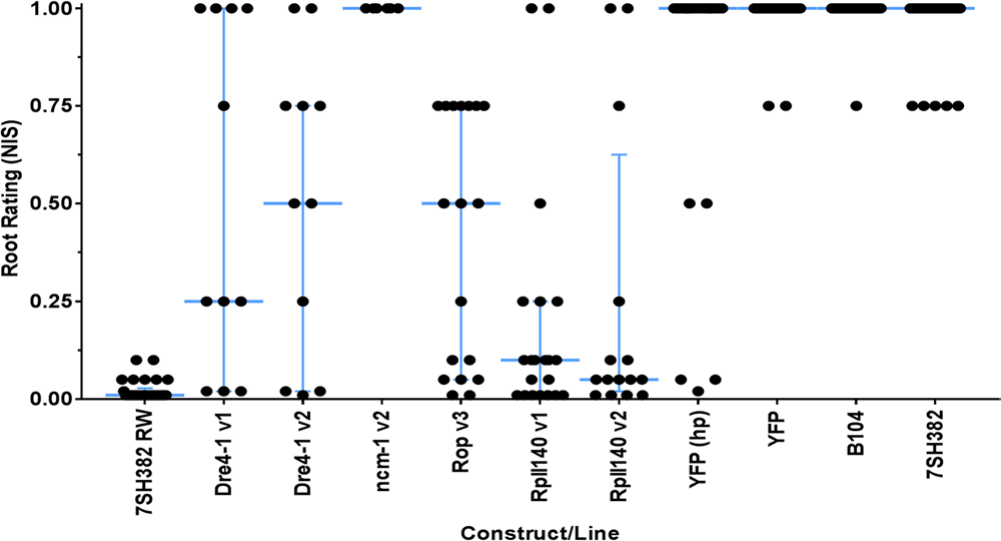
Root protection of T_0_ generation of transgenic RNAi maize plants. Root node injury score (NIS) ratings on scale from 0 to 1, with 1 indicating most root damage, of transgenic T_0_ maize plants expressing dsRNA hairpins targeting *dre4*, *ncm*, *Rop*, and *RpII140*, transcripts of *D*. *v*. *virgifera* were plotted in GraphPad Prism 7.03. Horizontal blue bars indicate median scores; the outer bars represent interquartile ranges. Homozygous line DAS59122–7, that expresses Cry34Ab1/35Ab1 in 7SH382 background and a transgenic maize plants expressing YFP in B104 background were used as positive and negative controls. Raw data is in Supplementary Table 6.

**Figure 4:**
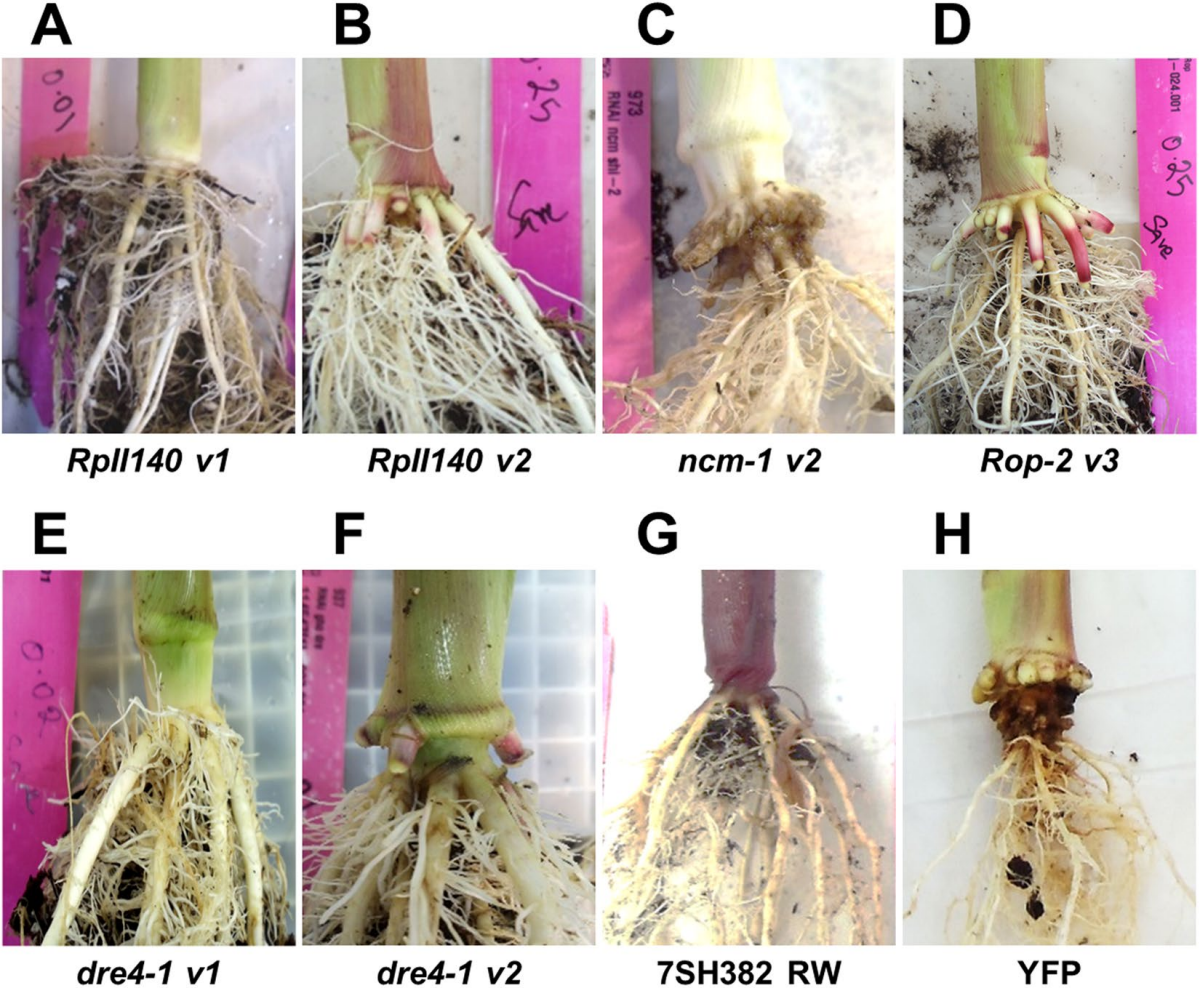
Images of representative maize roots of T_0_ maize hpRNA transformation events. Root images of representative **(A-B)** *RpII140*, **(C)** *ncm*, **(D)** *Rop*, *and* **(E,F)** *dre4* T_0_ plants at the end of root protection assay.

To determine if the selected RNAi targets confer root protection via hpRNAs, *Agrobacterium*-mediated maize transformation was performed. Maize transgenic plants were generated for hpRNAs *dre-1 v1*, *dre-1 v2*, *Rop-2 v3*, *RpII140 v1*, *RpII140 v2*, and *ncm-1 v2*. The *D*. *v*. *virgifera* bioassay for evaluation of T_0_ maize transgenic plants was run for fourteen days after infestation with the *D*. *v*. *virgifera* eggs. Figure 3 shows that *Rop*, *RpII140*, and *dre4* transgenes conferred high levels of root protection in multiple, independent T_0_ generation maize lines. The qualitative differences between hpRNA expressing plants and negative controls were also evident from plant photos (Fig. 4). The generalized linear mixed model analysis of root damage rating, based on a binomial response (pass or fail) revealed that the hpRNAs *dre-1 v1*, *dre-1 v2*, *Rop-2 v3*, *RpII140 v1*, and *RpII140 v2* had significantly higher proportion of plants with low root damage ratings compared to all negative controls and constructs expressing *hpncm-1 v2* (F_6, 15_ = 1230.6, *p* < 0.0001, Table 3). The plants expressing hpRNAs *RpII140 v1 and RpII140 v2* showed superior root protection with the highest frequency proportion of bioassay “passers” among the constructs evaluated, though not significantly different from hpRNAs *dre-1 v1*, *dre-1 v2*, and *Rop-2 v3*. The proportion of plants passing the bioassay was zero for non-transformed B104, non-transgenic isoline 7SH382, and transgenic plants expressing YFP protein, 0.09 for constructs expressing hairpin YFP and 1.0 for DAS59122–7 events expressing Cry34Ab1/35Ab1 (Table 3).

**Table 3:** T_0_ maize plants expressing *D*. *v*. *virgifera* hpRNA show robust root protection compared to negative controls. The root damage rating data estimated using 0–1.0 NIS scale was converted into binomial categorical data and the proportion of bioassay passers ( ≤ 0.5 root rating) in each constructs was compared against controls and other constructs were using SAS PROC GLIMMIX procedure (SAS 9.3, 2013).

maize genotype	Construct	# events tested	proportion of plants “passed”**	SEM
DAS59122–7	positive control	30	1a	0
hpRpII140 v1	pDAB114524	21	0.94b	0.05
hpRpII140 v2	pDAB114525	16	0.8b	0.14
hpRop-2 v3	pDAB115770	17	0.73b	0.17
hpdre4–1 v1	pDAB114546	11	0.46bc	0.26
hpdre4–1 v2	pDAB114547	11	0.46bc	0.26
hpncm-1 v2	pDAB119706	6	0d	0
YFP protein	pDAB101556	33*	0d	0
hpYFP	pDAB110853	33*	0.09c	0.06
Non-transformed B104	negative control	34	0d	0
Non-transgenic isoline 7SH382	negative control	32	0d	0

*Transgenic negative control events expressing either *yfp* hairpin dsRNA or YFP protein.

**Proportion values with the same letter are not significantly different.

### Core components of RNAi machinery are present in *M*. *aeneus*

**Figure 5:**
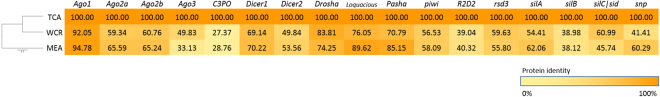
Identity of RNAi pathway genes in *T*. *castaneum*, *M*. *aeneus* and *D*. *v*. *virgifera*. The identity was based on protein-protein BLAST with query sequences from T. castaneu*m* (Supplementary Table 8).

An RNAi response to dsRNA as well as the RNAi pathway machinery have not been described in *M*. *aeneus*. Since a conserved RNAi machinery is a prerequisite for a robust and reliable RNAi response, we searched the transcriptomic data from *M*. *aeneus*^45^ for RNAi pathway genes by using *T*. *castaneum* homologs as queries. We identified all major RNAi pathway genes in the *M*. *aeneus* transcriptome, which include *Argonaute1 (Ago1)*, *Argonaute2a (Ago2a)*, *Argonaute2b (Ago2b)*, *Argonaute 3 (Ago3)*, *C3PO*, *Dicer1 (Dcr1)*, *Dicer2 (Dcr2)*, *Drosha*, *Loquacious*, *Pasha*, *piwi*, *R2D2*, *rsd3*, *snp* (Fig. 5 and Supplementary Table 8). Additionally, the putative systemic RNA interference defective (SID-like or SIL) genes *silA*, *silB*, *silC|sid* and corresponding proteins were also identified. The highest levels of protein identity were observed within the RNase III family, with over 70% identity for Dicer-1 and Drosha proteins, and for dsRNA binding proteins Loquacious and Pasha that are conserved at over 85% identity. Cofactor R2D2 has a lower identity of 40.3%, but comprises both RNA binding domains of the *Tribolum* reference sequence (data not shown). The proteins of the effector complex RISC with Ago1 (94.8%), Ago2a (65.6%), Ago2b (65.2%), Ago3 (33.1%), and piwi (58.1%) are conserved. A comparatively low homology could be detected with the RISC key activator *C3PO* (29%) and the interference defective genes of *silB* (38.1%) and *silC* (45.7%) (Fig. 5).

### dsRNA-induced RNAi causes high mortality in *M*. *aeneus*

**Figure 6:**
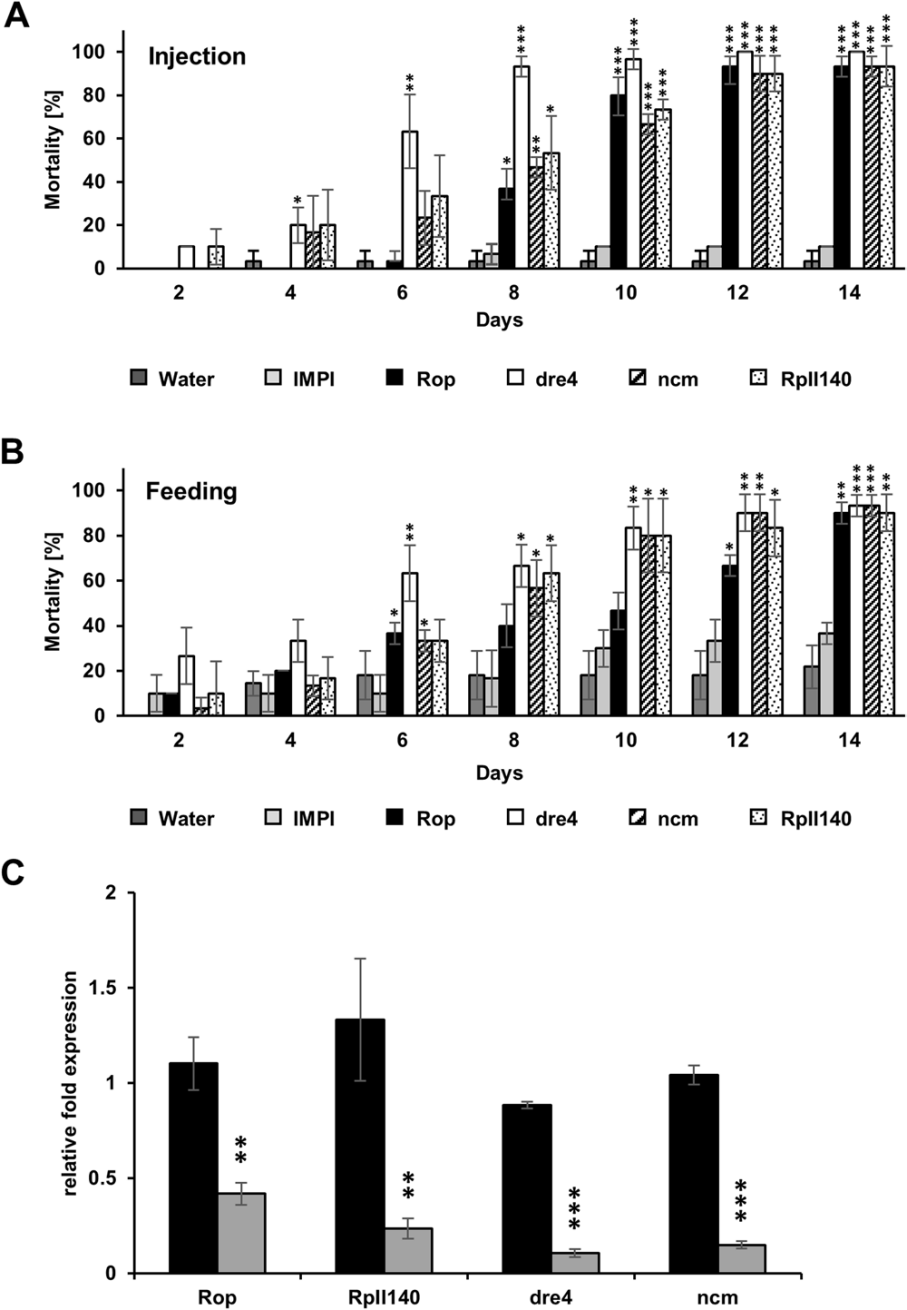
dsRNA induces RNAi effect and causes high mortality and silencing of gene expression in *M*. *aeneus*. **(A)** Adult *M*. *aeneus* were injected dsRNA at 250 ng/µl or **(B)** fed with 500 ng/cm^2^ with gene-specific dsRNA, targeting *Rop*, *dre4*, *ncm* or *RpII140* (controls: IMPI dsRNA and water). Mortality rates were checked every two days for 14 days. Three biological replicates (n = 10 per replicate) and were analyzed with analysis of variance (ANOVA) (Supplementary Table 4. **(C)** The mRNA knock-down of gene-specific dsRNA in *M*. *aeneus* were verified four days after injection by qPCR analyzes. Normalized gene expression levels were determined using the ΔΔC_T_ method, based on the gene of interest and reference gene *rps3*, relative to the *IMPI* control expression levels. Asterisk show statistical difference (*p < 0.05, **p < 0.01 and ***p < 0.001). The data represent three independent experiments. Mean values and standard deviations are provided in Supplementary Table 7.

The four most lethal target genes from the *D*. *v*. *virgifera* diet bioassays were selected for *M*. *aeneus* knock-down experiments to confirm functional RNAi. The ortholog sequences of *dre4*, *RpII140*, *Rop*, and *ncm* were identified from the *M*. *aeneus* transcriptome. Gene-specific dsRNAs were designed and injected into adult beetles (~150 nl of 250 ng/µl). Silencing of *dre4* (63.33% ± 17.00) showed statistically significant mortality 6 days post injection (dpi), whereas knock-down of *Rop* (80.00% ± 8.16), *ncm* (66.67% ± 4.71) or *RpII140* (73.33% ± 4.71) caused significant mortality 10 dpi (Fig. 6A and Supplementary Table 7). In all treatments, the mortality rate of RNAi treated groups reached over 90% 14 dpi, including 100% in the *dre4* dsRNA-treated group (Fig. 6A). The *p*-values for all statistical comparisons are shown in Supplementary Table 4. Real time quantitative PCR (qPCR) was performed on beetles injected with gene specific dsRNA to confirm silencing of the corresponding mRNA. All tested genes exhibited a significantly reduced transcript level four days after treatment (Fig. 6C and Supplementary Table 7). Decrease of *Rop* (62.00% ± 5.33), *dre4* (87.89% ± 2.45), *ncm* (85.65% ± 1.84), and *RpII140* (82.36% ± 4.00) expression were significant at 4 dpi.

Feeding bioassays using *Rop*, *dre4*, *ncm*, and *RpII140* dsRNAs (Fig. 6B) caused similar mortality rates in *M*. *aeneus* as observed in the injection assays. Significant mortality was induced six days after feeding on dsRNA for *dre4* (63.33% ± 12.47) and after 10 days for *Rop* (46.67% ± 9.43), *ncm* (80.00% ± 16.33) and *RpII140* (80.00% ± 16.33) (Fig. 6B, and Supplementary Tables 4 and 7). All treatments showed at least 90% mortality after two weeks of treatment (Fig. 6B).

### Conservation of Rop, dre4, and RpII140 proteins across three coleopteran insects

The domain analysis of Rop, RpII140, and dre4 showed that the domain architecture of these three proteins is conserved between the three species examined here (Supplementary Fig. 2). Further, at the amino acid level, Dre4 shows identity of 78.3%–82.9% across *T*. *castaneum*, *D*. *v*. *virgifera*, and *M*. *aeneus* (see Supplementary Materials, Sequence 7). *Dre4* nucleotide identity ranges from 67.0% to 71.9%, with a single contiguous match of 21 nucleotides or longer [22 nt match] (Supplementary Materials, Sequence 11). Rop has amino acid identity of 83.0%–87.9% across the three species (see Supplementary Materials, Sequence 9). In contrast to high level of identity at amino acid level, at nucleotide level, *Rop* retains 69.1% to 74.5% identity across the three species, with no 21-mer matches (Supplementary Materials, Sequence 10). RpII140 shows high level of identity at amino acid level as well, with identifies ranging from 94.6% to 97.1% (Supplementary Materials, Sequence 8). At nucleotide level RpII140 has 76.5% to 79.4% identity, with a 23-mer, 26-mer, and 29-mer contiguous matches between *D*. *v*. *virgifera* and *M*. *aeneus*; a 26-mer match between *D*. *v*. *virgifera* and *T*. *castaneum*, and two 23-mers and single 26-mer and 32-mer matches between *T*. *castaneum* and *M*. *aeneus* over the entire sequence span (Supplementary Materials, Sequence 12). None of the plant-expressed *D*. *v*. *virgifera* dsRNA sequences had matches of 21 nucleotides or longer to the corresponding genes of *T*. *castaneum* and *M*. *aeneus*. Nucleotide and amino acid sequences of *D*. *v*. *virgifera*, *T*. *castaneum*, and *M*. *aeneus* Rop, RpII140, and dre4 are in Supplementary Table 9.

## Discussion

Plant-delivered RNAi has recently been developed as an insect control method with the advantage of species-specific gene silencing. The success of RNAi approaches to crop protection will largely depend on a robust oral RNAi response in the target insect and the efficacy of the individual RNAi targets. This is because the transcript knockdown via RNAi seems to be fairly rapid and may occur as early as 24 hours or less after dsRNA feeding^17^. Thus, an RNAi-induced lethal phenotype largely depends on gene target attributes including the time-course of gene silencing, the function of the targeted gene product and the turnover of the target-encoded protein. Additional determinants of RNAi-induced insect mortality can also depend on the number of gene paralogs with substituting functions, gene expression patterns, and other factors. Based on these technical considerations, the most reliable method for identification of RNAi targets that are highly lethal on short timescale may be empirical screening in the pest of interest or, alternatively, in a model insect when pest genomic information is lacking. In this study, we selected 50 *T*. *castaneum* target genes, the majority of which were previously identified in iBeetle to be lethal^46^, in order to test if corresponding orthologous RNAi targets are lethal in the maize pest insect *D*. *v*. *virgifera*. The selected genes were first tested in serial dilutions in *T*. *castaneum* to identify highly efficacious targets at a low dose of dsRNA. Of the 50 RNAi gene targets, high proportion showed mortality or growth inhibition. Further, the RNAi targets that showed high lethality in *D*. *v*. *virgifera* were also highly effective at low dose in *T*. *castaneum*. Thus, pre-screening for high levels of RNAi efficacy in a model insect can greatly increase the probability of finding efficacious RNAi targets in another species. The utility of these gene targets in plant-directed RNAi was demonstrated by the root protection to WCR feeding in maize expressing *Rop*, *RpII140*, and *dre4* hairpin dsRNA. Not only did several individual T_0_ integration events for each RNAi construct show high levels of root protection, each group of these constructs showed efficacy that was significantly different from the negative controls.

RNAi targets *Rop*, *RpII140*, and *dre4* encode polypeptides with essential functions in important biological processes. The current list of plant-expressed dsRNAs that are known to confer root protection in maize includes: 1) vacuolar ATPase subunit A, *V-ATPase A*^15^, 2) vacuolar protein sorting gene of the (ESCRT-III) Endosomal Sorting Complex Required for Transport-III, *Snf7*^29^ (also called *Vps32* or *shrub* in *Drosophila*), 3) vacuolar ATPase subunit C, *V-ATPase C*^34^, and 4) smooth septate junction proteins *dvssj1* and *dvssj2*, which correspond to the orthologs *snakeskin* (*ssk*) and *mesh*, respectively^36^. Interestingly, RNAi target relationships become apparent even among the small number of root-protective RNAi targets for *D*. *v*. *virgifera*. Like Snf7 and V-ATPase A, Rop is involved in vesicular traffic within the cell, while RpII140, and dre4 are involved in transcription^47,48^. More specifically, dre4 (homolog of SPT16) is part of the FACT (facilitates chromatin transactions) complex and acts as a general chromatin regulator, recognizing nucleosomes^49,50^. RpII140 is part of the DNA-directed RNA polymerase II subunit that catalyzes the transcription of DNA into RNA^51,52^. The *Drosophila* Ras opposite (Rop) is a homolog of the yeast Sec. 1 protein (also known as Unc-18 from *C*. *elegans* or the rat Munc-18/n-Sec. 1/rbSec. 1 gene) and essential for vesicle trafficking and membrane fusion^53^. These housekeeping processes are essential to cell viability and represent opportunities to source additional genes for plant-delivered or environmentally-supplied RNAi for insect control. To determine if the observed lethal effect of the identified RNAi target genes identified in *T*. *castaneum* and validated in *D*. *v*. *virgifera* can also induce RNAi in another coleopteran pest, we selected an important oilseed rape pest *M*. *aeneus*. As RNAi has not yet been demonstrated in *M*. *aeneus*, we first analyzed transcriptomic data for RNAi pathway genes^54^. All major RNAi pathway genes (siRNA, miRNA, and piRNA) that we searched for are present in *M*. *aeneus*, supporting a functional RNAi mechanism. As *M*. *aeneus* bioassays relied on field-collected beetles, RNAi experiments were limited to the four most active target genes from the above *D*. *v*. *virgifera* assays: *Rop*, *dre4*, *ncm*, and *RpII140*. Both injection and feeding of dsRNA led to high mortality rates in *M*. *aeneus*. The injection of *dre4* dsRNA showed the most rapid effect. Subsequent quantitative qPCR further confirmed the suppression of the targeted mRNA levels. A significant level of transcript knockdown,, within mRNA extracted from whole beetles, suggests a systemic RNAi response in *M*. *aeneus*. Further, the feeding responses of *M*. *aeneus* demonstrate an environmental RNAi response in this pest. In total, these observations revealed a functional RNAi pathway, oral/environmental and systemic RNAi responses in *M*. *aeneus*, and confirmed the sequence-specific sensitivity for targets identified in *T*. *castaneum*.

High mortality rates observed in the *M*. *aeneus* feeding bioassay indicate a clear potential of utilizing RNAi as alternative control method for *M*. *aeneus*. A possible commercial approach for pollen beetle protection is via topical application by spraying dsRNA onto the host plants. Studies in the Colorado potato beetle (*Leptinotarsa decemlineata*) have shown that foliar application of dsRNA mediated plant protection that last for at least 28 days^55^, making this approach viable for further development. However, unlike *L*. *decemlineata*, the larval stages of *M*. *aeneus* reside inside the flower buds and might be not be exposed to typical foliar application. Thus, the development of plants expressing dsRNA constructs provides a promising alternative.

As RNAi targeting of genes described here was effective in three different coleopteran species, additional coleopteran pests might also be sensitive to their knockdown (*e*.*g*., other *Diabrotica* species, Colorado potato beetle (*Leptinotarsa decemlineata*), or wireworms (Coleoptera: Elateridae). The high level of identity at amino acid level across most of these proteins, with few or no 21-mer nucleotide identities, suggests that the RNAi response in Coleoptera may be generalized to the target type but is very specific to a target sequence^44,45^. We were able to identify gene orthologs in the preliminary transcriptome of *M*. *aeneus* that showed a robust lethal RNAi response. This enabled the transcript to be scanned for specific dsRNA trigger sequences devoid of potential off-target transcript matches, which can be as short as < 100 bp^16,34^. Taken together, the model organism screen approach in *Tribolium* followed by further refinement of potent RNAi targets in *Diabrotica* has enabled the selection of robust and specific RNAi targets for the difficult-to-work-with pollen beetle pest insect.

## Conclusions

This research identified in the model insect *T*. *castaneum* four novel RNAi targets that induce high mortality in *D*. *v*. *virgifera* feeding bioassays. We used all four target genes for the production of transgenic dsRNA crops, three of which show root protection against *D*. *v*. *virgifera* larvae by RNAi. Moreover, we showed, for the first time, functional RNAi in field collections of the agricultural pest *M*. *aeneus*. Our data demonstrate the feasibility of RNAi applications for combating pollen beetle and potentially other pests. Further, the identified gene targets can be used to better understand RNAi responses in non-pest insects. Taken together, lethal RNAi target genes identified in *T*. *castaneum* showed high activity in two additional coleopteran pest species that can pave the way to a new generation of species-specific plant protection. The principle of target identification that we explored in this study may be applied to other pest insects with the goal of developing insect resistance strategies.

## Methods

### Insect rearing

Wild-type *T*. *castaneum* San Bernardino beetles were maintained as described previously^56^. Adult *M*. *aeneus* were collected from fields with flowering *Brassica napus* plants in the surroundings of Giessen and kept on greenhouse-grown rape plants in a climate chamber with a 16-hour photoperiod and a day/night temperature of 24/18 °C. Non-diapausing *D*. *v*. *virgifera* eggs were purchased from Crop Characteristics, Inc. (Farmington, MN). *D*. *v*. *virgifera* eggs were washed from soil with water and surface-sterilized with 10% formaldehyde for three minutes^57^. The eggs were then rinsed with water and hatched on artificial diet at 28 °C, as described previously^58,59^.

### RNA extraction, library preparation and next-generation sequencing

Total RNA was extracted from *T*. *castaneum* mid-late larvae (L5) and last larval instars (LLI) using Direct-zol™ RNA MiniPrep (Zymo Research, Irvine, California, USA), according to the manufacturer’s instructions. The quality and quantity of RNA was determined using a Nanodrop ND-1000 spectrophotometer (NanoDrop Technologies Inc., Wilmington, Delaware, USA). Small RNA populations ( < 200 nt) were isolated and purified from *D*. *v*. *virgifera* using a mirVana™ miRNA Isolation Kit (Ambion Life Technologies). The Illumina TruSeq Small RNA Sample Prep Kit (Illumina, Inc., San Diego, CA, USA) was used according to the manufacturer’s protocol to convert small RNA into cDNA libraries. Final libraries were validated on the Bioanalyzer 2100 high-sensitivity DNA chip (Agilent Technologies, Santa Clara, CA, USA), normalized to 2 nm concentration and pooled (8 samples/pool). Each pool was then denatured with sodium hydroxide and diluted to 8 pM in hybridization buffer for loading onto a single lane of a HiSeq flow cell. Cluster generation, primer hybridization and sequencing reactions were carried out according to Illumina’s protocol.

*D*. *v*. *virgifera* total RNA was isolated and purified from approximately 0.9 g of whole first-instar larvae by following TRI REAGENT-Protocol (Sigma, St. Louis, MO, USA). The TruSeq stranded mRNA library preparation kit (Illumina, San Diego, CA, USA) was used to prepare mRNA libraries for 1^st^ instar *D*. *v*. *virgifera* by following instructions from the manufacturer’s recommended protocol. In brief, poly-A containing mRNA was purified from 4 µg of total RNA using poly-T oligo-attached magnetic beads. Purified mRNA fragments were subsequently fragmented into smaller pieces (about 300 bp average length) using divalent cations under elevated temperature. Next, SuperScript II reverse transcriptase (Thermo Fisher Scientific, Waltham, MA, USA) and random primers were used to copy the mRNA into first strand cDNA. The cDNA was further converted into double-stranded cDNA (ds cDNA). These ds cDNA fragments then underwent A-tailing and then ligation to indexed Illumina adapters. Lastly, adapter-ligated library products were cleaned up and enriched with 15 cycles of PCR and purified. The purified, enriched libraries were normalized to 2 nM concentration, denatured with sodium hydroxide, and diluted in hybridization buffer. Paired-end sequencing (2 × 151) was carried out on Illumina HiSeq. 2000 according to Illumina’s recommended protocol, yielding 87 million reads.

### Transcriptome assembly and gene expression analysis

*T*. *castaneum* raw reads from Illumina HiSeq were processed by CASAVA software (Illumina) for demultiplexing and removal of the primers attached to the reads. Fastq-mcf was used to trim the adaptors attached to the reads. The trimmed reads ranged from 18 to 30 bp, and the 20–24 bp reads were considered potential siRNAs for further analysis. These filtered reads were mapped to transgene sequence using Bowtie software with no mismatch allowed (Langmead *et al*. 2009). The mapped 21- and 24-nt reads were visualized using Integrative Genomics Viewer software (Broad Institute, Cambridge, MA, USA).

The transcriptome sequence data generated by Vogel, *et al*.^45^ was used for the identification of *M*. *aeneus* genes. Briefly, paired-end reads (2 × 100 nt) were acquired from Illumina HiSeq. 2500 with the error rate < 0.001 for 88% of bases. Quality of obtained reads was checked by fastQC (0.11.4). Trimming was performed by Trimmomatic v.0.36 (parameter: slidingwindow:4:5; leading:5; trailing:5; minlen:25). Sequences shorter 25 bp were discarded. The transcriptome *de novo* assembly was performed using Trinity (v.2.3.2.). Various assembly combinations were performed and analyzed by transrate (v1.0.3.). Resulting transcripts were aligned with the NCBI NR database by BLASTX search with an E-value cutoff of 1 × 10^−4^. The resulting BLAST hits were processed using Blast2GO software to classify transcripts into GO term categories, including molecular function, biological process, and cellular component. Additionally, the transcripts were translated in all six frames by transeq (EMBOSS package) and aligned by BLAST to the COG database with minimum protein identity of 50% and a protein coverage of at least 75% and lower than 125%.

To calculate gene expression levels and significance of expression differences, pairwise comparisons were performed in Cuffdiff, which is part of the Cufflinks package (2.2.1). Cuffdiff was used with geometric normalization and a threshold criteria for a false discovery rate (FDR) of 0.01. The expression levels were expressed as log2-fold-change of Fragments Per Kilobase per Million mapped reads (FPKM)-normalized count data.

*D*. *v*. *virgifera* raw sequencing data was processed using fastq-mcf to remove adaptors and low quality sequencing data with Q30 cut off. The transcriptome *de novo* assembly was performed on trimmed reads using Trinity (v.2.0.2) that generated 69,840 transcripts. This *de novo* transcriptome is used for further analyses.

### Identification of *T*. *castaneum* gene orthologs in *D*. *v*. *virgifera* and *M*. *aeneus*

Based on the genome sequence of *T*. *castaneum* ver.Tcas5.2, ortholog proteins of *M*. *aeneus* were identified by using NCBI BLASTP with an E-value of 0.01. The output were further filtered by a minimum protein identity of 50% as well as a protein coverage of at least 75% and lower than 125%. Resulting hits were ranked by score; redundant and overlapping sequences were removed.

To identify *T*. *castaneum* proteins in *D*. *v*. *virgifera*, TBLASTN searches using candidate protein coding sequences were run against BLASTable databases containing the unassembled *D*. *v*. *virgifera* sequence reads or the assembled contigs. Significant hits to a *D*. *v*. *virgifera* sequence (defined as lower than <1 × 10^−20^ for contig homologies and better than E-value of <1 × 10^−10^ for unassembled sequence reads homologies) were confirmed using BLASTX against the NCBI non-redundant database. The results of this BLASTX search confirmed that the *D*. *v*. *virgifera* homolog candidate gene sequences identified in the TBLASTN search indeed comprised *D*. *v*. *virgifera* genes, or were the best hits to the non *D*. *v*. *virgifera* candidate gene sequence present in the *D*. *v*. *virgifera* sequences. In most cases, *T*. *castaneum* candidate genes, which were annotated as encoding a protein, showed unambiguous sequence homology to a sequence or sequences within the *D*. *v*. *virgifera* transcriptome. In few cases, partially-overlapping contigs were assembled into longer contigs using Sequencher™ v4.9 (Gene Codes Corporation, Ann Arbor, MI).

### Identification of genes involved in RNAi pathway

Identification of core RNAi and potential systemic RNAi genes in *T*. *castaneum* was based on findings of Tomoyasu *et al*.^54^. The reference set of 17 proteins was aligned to the assembled and translated transcripts of *M*. *aeneus* and *D*. *v*. *virgifera* by BLASTP. Proteins with an identity greater than 50% and a minimum coverage of 75% were considered as homologs. Additionally, domain architecture was analyzed by ScanProsite and hits with equal or higher profile score than 10.0 indicated a domain occurrence. Hits with alignment scores below 8.5 are usually common, but were regarded as questionable and therefore excluded from further analysis.

### Template preparation and dsRNA design and synthesis

Total RNA was extracted from *T*. *castaneum* larvae and *M*. *aeneus* adults using Direct-zol™ RNA MiniPrep (Zymo Research, Irvine, California, USA) according to the manufacturer’s instructions. cDNA was reverse transcribed from 500 ng RNA using the First Strand cDNA Synthesis Kit (Thermo Fisher Scientific, Waltham, MA). Appropriate PCR templates (Supplementary Table 1) were generated with gene-specific RNAi primers including the T7 promoter sequence (TAATACGACTCACTATAGGGAGA) at the 5′, purchased from Sigma-Aldrich (St Louis, MO, USA). Ambion MEGAscript T7 kit (Thermo Fisher Scientific, Waltham, MA) was used to prepare dsRNA according to the manufacturer’s protocol.

#### T. castaneum RNAi design

Unassembled *D*. *v*. *virgifera* sequence reads or the assembled contigs identified to contain either *T*. *castaneum* RNAi target orthologs or RNAi pathway targets were annotated with the location of the open reading frame (ORF) for ortholog based on BLASTX results from NCBI non-redundant database. Using the ORF location dsRNA sequence was designed to be between 200 and 500 base pairs with a %GC between 40 and 60 and a distance from the ATG and stop codon of greater than seventy base pairs.

Total RNA was extracted from *D*. *v*. *virgifera* eggs, larvae, and adults using the TRIzol (Life Technologies, Grand Island, NY) isolation method according to the manufacturer’s instructions. cDNA was synthesized from 1 µg of total RNA using a SuperScript III reverse transcription kit (Thermo Fisher Scientific, Waltham, MA). Oligonucleotide primers for PCR template production were designed using VectorNTI (Invitrogen, Carlsbad, CA) or Primer3 software and contained a T7 promoter sequence at their 5´ ends. dsRNAs were synthesized using Ambion MEGAscript T7 (Thermo Fisher Scientific, Waltham, MA) dsRNA synthesis kit, according to the manufacturer’s protocol. Sequences within the open reading frame of up to approximately 500 bp were selected for the initial dsRNA bioassay. The sequences of *D*. *v*. *virgifera* dsRNA amplicons appear in Supplementary Table 1. Synthesized dsRNA was quantified on NanoDrop 8000 spectrophotometer (Thermo Fisher Scientific, Waltham, MA) and diluted in 0.1 × TE to a working concentration of 12.5 ng/µl.

### Injection bioassays

*T*. *castaneum* larvae and *M*. *aeneus* adults were anaesthetized on ice before affixation to double-stick tape. Dorsolateral injection of 150 nl dsRNA [250 ng/µl] into each of the insects was performed using a pulled glass capillary (Item No: 504949, World Precision Instruments, Sarasota, FL) and the micromanipulator M3301 (World Precision Instruments, Sarasota, FL) under a dissecting stereomicroscope (n = 12, three replicates). Negative controls received an equivalent amount of water or dsRNA corresponding to the *Galleria mellonella* metalloproteinase inhibitor (IMPI) gene (AY330624), which is absent in beetles. *T*. *castaneum* larvae were kept on whole-grain flour with 5% yeast powder after injection, whereas *M*. *aeneus* beetles were kept in petri dishes with dried pollen and wet tissues as food and water sources. Survival rates were monitored and counted every 48 h for 14 days.

### Artificial diet bioassays

*M*. *aeneus* adults were kept without water for 24 h before treatment to ensure that beetles drank from a droplet of 5 µl dsRNA solution (1 µg/µl). After visual examination of dsRNA solution uptake, beetles were transferred to petri dishes with artificial diet and a wet tissue. The water-based diet (1% gelatin and 50% homogenized pollen) was mixed with dsRNA to a final concentration of 500 ng/cm^2^, to ensure continuous uptake of dsRNA (n = 10, three replicates). Negative controls received an equivalent amount of water or dsRNA corresponding to the *Galleria mellonella* metalloproteinase inhibitor (IMPI) gene (AY330624). As the recipe did not contain any antibiotics or antimycota, the diet was exchanged every two days to avoid fungal contamination. Mortality rates were checked every two days.

The *D*. *v*. *virgifera* feeding bioassays were conducted with neonate larvae (two to three larvae per well) in 128-well plastic bioassay trays (BIO-BA-128, C-D International, Pitman, NJ) that contained 1.5 ml of an artificial diet^60^. DsRNA in 0.1 × TE was surface-applied at 500 ng/cm^2^. Trays were sealed with Pull N’ Peel Tab vented adhesive plastic sheets (BIO-CV-16, C-D International, Pitman, NJ) and held at 28 °C, ~40% Relative Humidity. The total number of insects exposed to each sample, the number of dead insects, and the weight of surviving insects were recorded after nine days. DsRNA targeting *yellow fluorescent protein gene* (YFP), 0.1XTE buffer, and water were used as negative controls. Growth Inhibition (GI) was calculated based on the average weights of all controls, as follows:

${\rm{G}}{\rm{I}}=[1-({\rm{T}}{\rm{W}}{\rm{I}}{\rm{T}}/{\rm{T}}{\rm{N}}{\rm{I}}{\rm{T}})/({\rm{T}}{\rm{W}}{\rm{I}}{\rm{B}}{\rm{C}}/{\rm{T}}{\rm{N}}{\rm{I}}{\rm{B}}{\rm{C}})],$ where TWIT is the Total Weight of live Insects in the Treatment; TNIT is the Total Number of Insects in the Treatment; TWIBC is the Total Weight of live Insects in the Background Check (negative control); and TNIBC is the Total Number of Insects in the Background Check (negative control). To assess the potency of active RNAi targets, four-fold serial dilutions of dsRNAs were bioassayed and the LC_50_ (concentration at which 50% of the insects are dead) and GI_50_ (concentration that causes 50% growth inhibition or GI) values were calculated using log-logistic regression analysis in JMP Pro from SAS Institute Inc; raw data appears in Supplementary Table 2.

### Transcript knockdown verification in *M*. *aeneus*

Total RNA was isolated from flash-frozen adult beetles zero and four days after injection (n = 15; 3 replicates) using Direct-zol RNA MiniPrep (Zymo Research, Irvine, California, USA). The quantity and quality of RNA was validated by NanoDrop ND-1000 spectrophotometer (NanoDrop Technologies Inc., Wilmington, DE, USA). cDNA was synthesized from 500 ng of RNA with oligo(dT) primers and the First Strand cDNA Synthesis Kit (Thermo Fisher Scientific, Waltham, MA, USA). Quantitative real-time PCR was performed on a StepOnePlus system using TaqMan Gene Expression Assays with TaqMan Universal Master Mix II, and 50 ng of cDNA per reaction (Thermo Fisher Scientific, Waltham, MA, USA). The ribosomal protein gene *Rps6* was used as an endogenous control. Relative gene expression was calculated based on three biological replicates, each with two technical replicates, according to Pfaffl^61^.

### Hairpin RNA and plasmid design

Subsequences of the *D*. *v*. *virgifera dre4*, *ncm*, *Rop*, and *RpII140* were selected for maize transformation. These sequences were selected to be devoid of known maize splice sites and 21-mers or longer matches to the *Apis mellifera* (honey bee), *Bombus terrestris* (bumblebee), and *Mus musculus* (mouse) transcriptomes. Sequences *dre4–1 v1*, *dre4–1 v2*, *ncm-1 v2*, *Rop-2 v3*, *RpII140 v1*, and *RpII140 v2* contained no 21-mer or longer matches to *A*. *mellifera*, *B*. *terrestris* and *M*. *musculus*.

Standard cloning methods were used in the construction of Gateway-enabled entry vectors (Invitrogen, Carlsbad, CA) containing RNAi hairpin expression cassettes. Hairpins were designed to include target sense and antisense sequences separated by a flexible linker. Expression of the hairpin was driven by the promoter from the maize *ubiquitin 1* (*Ubi-1*) gene^62^ and terminated by the 3′ untranslated region of the maize peroxidase 5 gene^63^. Each hairpin containing entry vector was recombined using Gateway technology (Invitrogen, Carlsbad, CA) with a destination vector harboring a selectable marker cassette to create binary vectors for maize transformation.

### Development of transgenic plants

Binary expression vectors, based on pTI15955 plasmid from *Agrobact-erium*^64^, contained *hpdre4–1 v1*, *hpdre4–1 v2*, *hpRop-2 v3*, *hpncm-1 v2*, *hpRpII140 v1*, and *hpRpII140 v2* hairpins. Each of these plasmids were transformed into *Agrobacterium tumefaciens* strain DAt13192 (RecA-deficient ternary strain)^65^. Colonies were selected and plasmid DNA was isolated and confirmed via restriction enzyme digestion. Each binary vector was separately transformed into maize via *Agrobacterium*-mediated transformation of immature embryos isolated from the inbred line, *Zea mays* c.v. B104 using conventional methods with modifications^66^. Briefly, the immature embryos were incubated with a suspension containing *Agrobacterium* cells and surfactant and then were moved to solid medium plates followed by co-cultivation for 3–5 days. The treated embryos were transferred onto a medium containing antibiotics and compounds for selective isolation of genetically transformed corn tissues. The corn tissues were grown on selection medium until plants were regenerated.

### Transgene copy number analysis

The described binary destination vector contained an herbicide tolerance gene (aryloxyalknoate dioxygenase; AAD-1 v3), under the expression regulation of a maize Ubi-1 promoter and a fragment containing a 3′ untranslated region from a maize lipase gene (ZmLip 3’UTR). DNA Real-time PCR analyses to detect a portion of the AAD1 coding region in gDNA were used to estimate transgene insertion copy number. The AAD levels were compared to the levels of single-copy native gene. Simple events (having one or two copies of transgene insertions) were selected for greenhouse bioassay. Additionally, PCR assays designed to detect a portion of the spectinomycin-resistance gene (SpecR; from the binary vector plasmids outside of the T-DNA) were used to determine if the transgenic plants contain extraneous integrated plasmid backbone. Samples for these analyses were collected from plants grown in environmental chambers at the V2–V3 growth stage. Maize leaf pieces approximately equivalent to two leaf punches were collected in 96-well collection plates (QIAGEN). Tissue disruption was performed with a KLECKO™ tissue pulverizer (Garcia Manufacturing, Visalia, CA) in BioSprint 96 AP1 lysis buffer (supplied with a BioSprint 96 DNA Plant Kit; QIAGEN) with one stainless steel bead. Following tissue maceration, gDNA was isolated in high throughput format using a BioSprint 96 DNA Plant Kit and a BioSprint 96 extraction robot. gDNA is diluted 2:3 DNA:water prior to setting up the qPCR reaction.

#### qPCR analysis

Transgene detection was performed by hydrolysis probe real-time quantitative PCR assay. Primers and probe to detect a portion of the SpecR gene (SPC1) and a segment of the AAD-1 herbicide tolerance gene (GAAD1) appear in Supplementary Table 10. Assays were multiplexed with reagents for an endogenous maize chromosomal Invertase gene (IVR1, GENBANK Accession No: U16123), which served as an internal reference sequence to ensure gDNA is present in each assay. PCR amplification was set up using LightCycler 480 Probes Master mix (Roche). PCR was as performed on LightCycler 480 Instrument (Roche) using fluorophore activation and emission for the FAM- and HEX-labeled probes. Cp scores (the point at which the fluorescence signal crosses the background threshold) are determined from the real time PCR data using the fit points algorithm (LightCycler 480 Software, Version 1.5) and the Relative Quant module (based on ΔΔC_T_ method).

### Root protection assays

The whole plant maize bioassays were conducted by following the protocol described in Dönitz, *et al*.^46^. In brief, the transgenic corn plants (T_0_, one plant per event) were planted into root trainer pots containing Metromix soil after reaching V2 or V3 stage. The plants were infested with 125–150 *D*. *v*. *virgifera* eggs and allowed to grow for two weeks. Two weeks after infestation, the plant roots were washed and rootworm feeding damage was scored using node-injury scale (NIS) ranging from 0 to 1 as compared to 0 to 3 described by Oleson, *et al*.^67^. Event DAS59122-7, expressing Cry34Ab1/Cry35Ab1, served as Bt positive control. The negative controls included non-transformed B104, B104 plants expressing either *yfp* hairpin dsRNA or YFP protein, and non-transgenic isoline of DAS59122–7, 7SH382. The constructs expressing RNAi targets were bioassayed at different times with both positive and negative controls included in each experiment.

### Statistical analysis

Analyses of variance (ANOVA) for *T*. *castaneum* and *M*. *aeneus* bioassays were followed by a Holm-Sidak test with significance threshold of p < 0.05 using Daniel’s XL toolbox for Excel, version 7.2.10 ^68^. Each experiment was compared to a control group and all experiments were conducted independently at least three times. For *D*. *v*. *virgifera* experiments, means comparisons were performed for all pairs using Tukey-Kramer HSD method in JMP Pro 11.1.1 (SAS, Cary, NC).

The T_0_ root damage rating data are not normally distributed; hence they were converted into categorical data which follows binomial distribution. All T_0_ events and control plants that showed a root rating of ≤ 0.5 were designated as “pass” and the events with root ratings > 0.5 to 1.0 were called “fail”. To identify the constructs that provided better root protection, the proportion of plants which passed the bioassay was analyzed with the generalized linear mixed model procedure^69^ (SAS PROC GLIMMIX, SAS 2013): ${\eta }_{ij}=\eta +Construc{t}_{i}+Test\,dat{e}_{j}$ with observations binomially distributed, *y*_*ij*_ ~ *Binomial* (*N*_*ij*_, *π*_*ij*_). The link function for the binomial distribution is the logit function ${\eta }_{ij}=log[{\pi }_{ij}/1-{\pi }_{ij}]$. Construct is modeled as a fixed effect and test date is modeled as random effect.

### Data availability

All data generated or analyzed during this study are included in this published article (and its Supplementary Information files).

## Electronic supplementary material

Supplementary Tables

Supplementary Figures and Materials
