# Supplementary material for: Gene silencing in Tribolium castaneum as a tool for the targeted identification of candidate RNAi targets in crop pests
Source: Sci Rep. 2018 Feb 1;8:2061. doi: 10.1038/s41598-018-20416-y (PMC5794766; doi:10.1038/s41598-018-20416-y)

**Gene silencing in *Tribolium* *castaneum* as a tool for the targeted identification of candidate RNAi targets in crop pests**

Eileen Knorr ^a^, Elane Fishilevich ^b^, Linda Tenbusch ^a^, Meghan L. F. Frey ^b^, Murugesan Rangasamy ^b^, Andre Billion ^a^, Sarah E. Worden ^b^, Premchand Gandra ^b^, Kanika Arora ^b^, Wendy Lo ^b^, Greg Schulenberg ^b^, Pablo Valverde-Garcia ^b^, Andreas Vilcinskas ^a,c^ and Kenneth E. Narva ^b*^

^a^ Fraunhofer Institute for Molecular Biology and Applied Ecology, Department of Bioresources, Winchester Str. 2, 35394 Giessen, Germany.

^b^ Dow AgroSciences, 9330 Zionsville Road, Indianapolis, IN, 46268, United States.

^c^ Institute for Insect Biotechnology, Heinrich-Buff-Ring 26-32, 35392 Giessen, Germany.

^*^ KNarva@dow.com

**Supplementary Figures and Materials**

**A B**

**Supplementary Figure 1. *D. v. virgifera* percent mortality and growth inhibition plotted against first instar log_2_-transformed transcript expression values in Fragments Per Kilobase of transcript per Million mapped reads (FPKM).** The expression values transcripts corresponding to 82 *D. v. virgifera* dsRNAs (see supplementary Table 1) that target 50 genes (see Table 1) were obtained from the transcriptome (Methods). Linear fit (R^2^) values were calculated in JMP® Pro 12.2.0. A. Scatter plot of percent larval mortality vs. transcript expression; R^2^ = 0.09. B. Scatter plot of larval growth inhibition (GI) vs. transcript expression; R^2^ = 0.11. Low R^2^ values indicate that there is no correlation between transcript expression and bioassay efficacy.

**
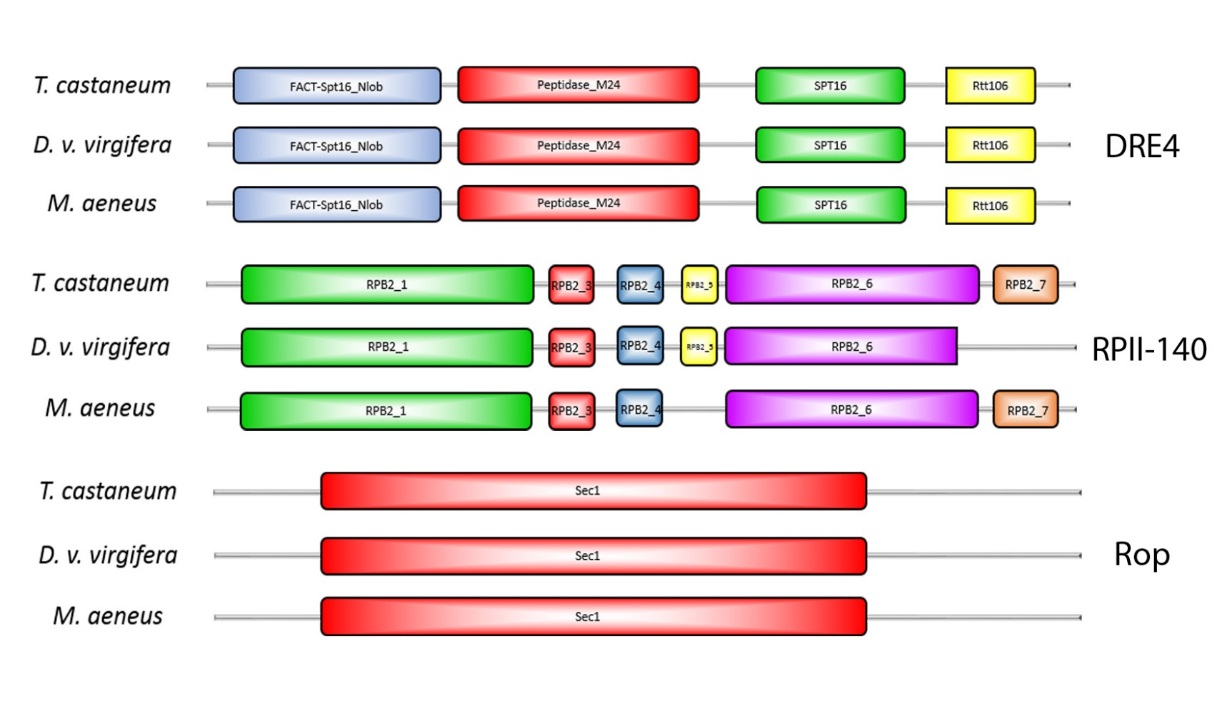
**

**Supplementary Figure 2. Domain architecture of three RNAi target genes.** Domain architecture of the three lethal genes of *T. castaneum*, *M. aeneus* and *D. v. virgifera* was analyzed by Scan-Prosite with PROSITE 2017_1 and annotated against Pfam domain families v.31. Domain hits with a Pfam bit-score above 50 and a Prosite similarity score above 8.5 indicated motif occurrence and were annotated.

**Sequence 1:** Rop Open Reading Frame (ORF), Rop Region 1, Rop Region 2, and Rop v3 Alignment. Blue sequence is region red sequence is version.

1 50

Dvv_Rop_ORF (1) ---ATGGCGTTAAAGAACCAAGTTGGTCAAAAAATCATGAATGAAGTCAT

Dvv_Rop_Region_1 (1) ACCATGGCGTTAAAGAACCAAGTTGGTCAAAAAATCATGAATGAAGTCAT

Dvv_Rop_Region_2 (1) --------------------------------------------------

Dvv_Rop-2_v3 (1) ------------------------------------------------

51 100

Dvv_Rop_ORF (48) CAAGCACAAGCCCACCAAGAAGAATGGGCCAACTCCAGGACAGCAAGCCC

Dvv_Rop_Region_1 (51) CAAGCACAAGCCCACCAAGAAGAATGGGCCAACTCCAGGACAGCAAGCCC

Dvv_Rop_Region_2 (1) --------------------------------------------------

Dvv_Rop-2_v3 (1) --------------------------------------------------

101 150

Dvv_Rop_ORF (98) ATGGGGTAGAATGGAGGATCCTTGTGGTGGACCAGCTTGCCATGAGGATG

Dvv_Rop_Region_1 (101) ATGGGGTAGAATGGAGGATCCTTGTGGTGGACCAGCTTGCCATGAGGATG

Dvv_Rop_Region_2 (1) --------------------------------------------------

Dvv_Rop-2_v3 (1) --------------------------------------------------

151 200

Dvv_Rop_ORF (148) GTTTCAGCATGCTGTAAAATGCATGATATATCAGCAGAAGGCATTACATT

Dvv_Rop_Region_1 (151) GTTTCAGCATGCTGTAAAATGCATGATATATCAGCAGAAGGCATTACATT

Dvv_Rop_Region_2 (1) --------------------------------------------------

Dvv_Rop-2_v3 (1) ----------------------------------------------

201 250

Dvv_Rop_ORF (198) GGTTGAAGATATTATGAAGAAAAGGGAACCGCTTGGTACCATGGAAGCTG

Dvv_Rop_Region_1 (201) GGTTGAAGATATTATGAAGAAAAGGGAACCGCTTGGTACCATGGAAGCTG

Dvv_Rop_Region_2 (1) --------------------------------------------------

Dvv_Rop-2_v3 (1) --------------------------------------------------

251 300

Dvv_Rop_ORF (248) TGTACTTGATAACACCTTCAGAAAAGTCAGTTCATGCTCTTATGAATGAC

Dvv_Rop_Region_1 (251) TGTACTTGATAACACCTTCAGAAAAGTCAGTTCATGCTCTTATGAATGAC

Dvv_Rop_Region_2 (1) --------------------------------------------------

Dvv_Rop-2_v3 (1) --------------------------------------------------

301 350

Dvv_Rop_ORF (298) TTTGAACCACCAAGACAGATGTACAGAGGGGCACACGTGTTTTTTACAGA

Dvv_Rop_Region_1 (301) TTTGAACCACCAAGACAGATGTACAGAGGGGCACACGTGTTTTTTACAGA

Dvv_Rop_Region_2 (1) --------------------------------------------------

Dvv_Rop-2_v3 (1) --------------------------------------------------

351 400

Dvv_Rop_ORF (348) AGCGTGTCCAGACCAATTATTTAGTACCTTGTGCCACCACCCCGTAGCAA

Dvv_Rop_Region_1 (351) AGCGTGTCCAGACCAATTATTTAGTACCTTGTGCCACCACCC--------

Dvv_Rop_Region_2 (1) --------------------------------------------------

Dvv_Rop-2_v3 (1) --------------------------------------------------

401 450

Dvv_Rop_ORF (398) AGTTTATTAAAACCCTAAAAGAAATCAACATAGCATTCATTCCGACTGAG

Dvv_Rop_Region_1 (393) --------------------------------------------------

Dvv_Rop_Region_2 (1) --------------------------------------------------

Dvv_Rop-2_v3 (1) --------------------------------------------------

451 500

Dvv_Rop_ORF (448) TCACAGGTGTTCTCATTGGATTCACCAGACACGTTCCAGTGTAGCTACGA

Dvv_Rop_Region_1 (393) --------------------------------------------------

Dvv_Rop_Region_2 (1) --------------------------------------------------

Dvv_Rop-2_v3 (1) --------------------------------------------------

501 550

Dvv_Rop_ORF (498) TCCATCATTTTCCGCTGCTAGAAACGCCAACATGGAAAGAATGGCAGAAC

Dvv_Rop_Region_1 (393) --------------------------------------------------

Dvv_Rop_Region_2 (1) --------------------------------------------------

Dvv_Rop-2_v3 (1) --------------------------------------------------

551 600

Dvv_Rop_ORF (548) AAATTGCGACACTCTGTGCGACTCTAGGGGAATACCCACACGTCAGATAT

Dvv_Rop_Region_1 (393) --------------------------------------------------

Dvv_Rop_Region_2 (1) --------------------------------------------------

Dvv_Rop-2_v3 (1) --------------------------------------------------

601 650

Dvv_Rop_ORF (598) AGAACTGATTGGGAAAGAAATGTTGAGCTGGCTCAACTAATTCAGCAGAA

Dvv_Rop_Region_1 (393) --------------------------------------------------

Dvv_Rop_Region_2 (1) --------------------------------------------------

Dvv_Rop-2_v3 (1) --------------------------------------------------

651 700

Dvv_Rop_ORF (648) ATTGGACGCCTATAAAGCCGACGAACCTACCATGGGAGAGGGGCCGGAAA

Dvv_Rop_Region_1 (393) --------------------------------------------------

Dvv_Rop_Region_2 (1) --------------------------------------------------

Dvv_Rop-2_v3 (1) --------------------------------------------------

701 750

Dvv_Rop_ORF (698) AGGCGAGATCACAATTAATTATCCTCGACCGAGGTTTCGACTGTGTATCT

Dvv_Rop_Region_1 (393) --------------------------------------------------

Dvv_Rop_Region_2 (1) -----------------------CTCGACCGAGGTTTCGACTGTGTATCT

Dvv_Rop-2_v3 (1) --------------------------------------------------

751 800

Dvv_Rop_ORF (748) CCCCTTCTTCACGAACTTACTTTCCAAGCAATGGCCTATGACTTACTACC

Dvv_Rop_Region_1 (393) --------------------------------------------------

Dvv_Rop_Region_2 (28) CCCCTTCTTCACGAACTTACTTTCCAAGCAATGGCCTATGACTTACTACC

Dvv_Rop-2_v3 (1) --------------------------------------------------

801 850

Dvv_Rop_ORF (798) CATAGAAAATGATGTATATAAGTACGAAGCATCGGCTGGTGTTATGAAAG

Dvv_Rop_Region_1 (393) --------------------------------------------------

Dvv_Rop_Region_2 (78) CATAGAAAATGATGTATATAAGTACGAAGCATCGGCTGGTGTTATGAAAG

Dvv_Rop-2_v3 (1) --------------------------------------------------

851 900

Dvv_Rop_ORF (848) AAGTCCTTCTAGACGAAAACGACGAGCTTTGGGTCGATCTACGCCACCAA

Dvv_Rop_Region_1 (393) --------------------------------------------------

Dvv_Rop_Region_2 (128) AAGTCCTTCTAGACGAAAACGACGAGCTTTGGGTCGATCTACGCCACCAA

Dvv_Rop-2_v3 (1) --------------------------------------------------

901 950

Dvv_Rop_ORF (898) CACATCGCGGTGGTGTCTCAGAGCGTCACCAAGAATCTGAAGAAATTCAC

Dvv_Rop_Region_1 (393) --------------------------------------------------

Dvv_Rop_Region_2 (178) CACATCGCGGTGGTGTCTCAGAGCGTCACCAAGAATCTGAAGAAATTCAC

Dvv_Rop-2_v3 (1) --------------------------------------------------

951 1000

Dvv_Rop_ORF (948) CGACTCCAAACGCATGACCCAGAGCGACAAGCAGTCGATGAAGGATCTCT

Dvv_Rop_Region_1 (393) --------------------------------------------------

Dvv_Rop_Region_2 (228) CGACTCCAAACGCATGACCCAGAGCGACAAGCAGTCGATGAAGGATCTCT

Dvv_Rop-2_v3 (1) --------------------------------------------------

1001 1050

Dvv_Rop_ORF (998) CAACCATGATCAAAAAGATGCCGCAATATCAGAAAGAATTGTCCAAGTAT

Dvv_Rop_Region_1 (393) --------------------------------------------------

Dvv_Rop_Region_2 (278) CAACCATGATCAAAAAGATGCCGCAATATCAGAAAGAATTGTCCAAGTAT

Dvv_Rop-2_v3 (1) -------------------------------------------CAAGTAT

1051 1100

Dvv_Rop_ORF (1048) GCTACGCATCTTCATCTCGCTGAAGACTGCATGAAGGCCTATCAGGGGTA

Dvv_Rop_Region_1 (393) --------------------------------------------------

Dvv_Rop_Region_2 (328) GCTACGCATCTTCATCTCGCTGAAGACTGCATGAAGGCCTATCAGGGGTA

Dvv_Rop-2_v3 (8) GCTACGCATCTTCATCTCGCTGAAGACTGCATGAAGGCCTATCAGGGGTA

1101 1150

Dvv_Rop_ORF (1098) TATAGACAAGTTGTGTAAAGTTGAGCAGGATTTGGCAATGGGAACTGATG

Dvv_Rop_Region_1 (393) --------------------------------------------------

Dvv_Rop_Region_2 (378) TATAGACAAGTTGTGTAAAGTTGAGCAGGATTTGGCAATGGGAACTGATG

Dvv_Rop-2_v3 (58) TATAGACAAGTTGTGTAAAGTTGAGCAGGATTTGGCAATGGGAACTGATG

1151 1200

Dvv_Rop_ORF (1148) CCGAAGGCGAGAAAATCAAGGATCACATGCGCAACATCGTCCCCATCTTG

Dvv_Rop_Region_1 (393) --------------------------------------------------

Dvv_Rop_Region_2 (428) CCGAAGGCGAGAAAATCAAGGATCACATGCGCAACATCGTCCCCATCTTG

Dvv_Rop-2_v3 (108) CCGAAGGCGAGAAAATCAAGGATCACATGCGCAACATCGTCCCCATCTTG

1201 1250

Dvv_Rop_ORF (1198) CTAGATCCCAAAATCACCAATGAATACGATAAGATGCGTATTATAGCATT

Dvv_Rop_Region_1 (393) --------------------------------------------------

Dvv_Rop_Region_2 (478) CTAGATCCCAAAATCACCAATGAATACGATAAGATGCGTATTATAGCATT

Dvv_Rop-2_v3 (158) CTAGATCCCAAAATCACCAATGAATACGATAAGA----------------

1251 1300

Dvv_Rop_ORF (1248) GTACGCCATGACGAAAAACGGCATCACAGATGAAAATCTCTCCAAATTGG

Dvv_Rop_Region_1 (393) --------------------------------------------------

Dvv_Rop_Region_2 (528) GTACGCCATGACGAAAAACGGCATCACAGATGAAAATCTCTCCAAATTGG

Dvv_Rop-2_v3 (192) --------------------------------------------------

1301 1350

Dvv_Rop_ORF (1298) CTACCCATGCCCAAATCAAGGACAAACAGACCATCGCCAACCTTCAGTTA

Dvv_Rop_Region_1 (393) --------------------------------------------------

Dvv_Rop_Region_2 (578) CTACCCATGCCCAAATCAAGGACAAACAGACCATCGCCAACCTTCAGTTA

Dvv_Rop-2_v3 (192) --------------------------------------------------

1351 1400

Dvv_Rop_ORF (1348) CTTGGAGTCAACGTTATTAATGATGGAGGACCAAGAAAAAAACAATATAC

Dvv_Rop_Region_1 (393) --------------------------------------------------

Dvv_Rop_Region_2 (628) --------------------------------------------------

Dvv_Rop-2_v3 (192) --------------------------------------------------

1401 1450

Dvv_Rop_ORF (1398) AGTACCGCGCAAAGAAAGAATTACAGAACAAACGTACCAAATGTCAAGAT

Dvv_Rop_Region_1 (393) --------------------------------------------------

Dvv_Rop_Region_2 (628) --------------------------------------------------

Dvv_Rop-2_v3 (192) --------------------------------------------------

1451 1500

Dvv_Rop_ORF (1448) GGACACCTATCATTAAGGATATAATGGAGGATTGCATAGACGACAAACTG

Dvv_Rop_Region_1 (393) --------------------------------------------------

Dvv_Rop_Region_2 (628) --------------------------------------------------

Dvv_Rop-2_v3 (192) --------------------------------------------------

1501 1550

Dvv_Rop_ORF (1498) GATCAGAAACACTACCCGTATTTGAGCGGACGAGCACAGTCTACGGGATA

Dvv_Rop_Region_1 (393) --------------------------------------------------

Dvv_Rop_Region_2 (628) --------------------------------------------------

Dvv_Rop-2_v3 (192) --------------------------------------------------

1551 1600

Dvv_Rop_ORF (1548) CCATGCAGCGCCCTCTAGTGCCCGTTATGGCCAGTGGCACAAAGACAGAG

Dvv_Rop_Region_1 (393) --------------------------------------------------

Dvv_Rop_Region_2 (628) --------------------------------------------------

Dvv_Rop-2_v3 (192) --------------------------------------------------

1601 1650

Dvv_Rop_ORF (1598) GTCAACAAGCCGTGAAGAACGTTCCTCGACTGCTCGTCTTCGTCGTGGGT

Dvv_Rop_Region_1 (393) --------------------------------------------------

Dvv_Rop_Region_2 (628) --------------------------------------------------

Dvv_Rop-2_v3 (192) --------------------------------------------------

1651 1700

Dvv_Rop_ORF (1648) GGAATCAGTTTTTCAGAGATCAGGTGCGCCTACGAAGTGACCAACGCGCA

Dvv_Rop_Region_1 (393) --------------------------------------------------

Dvv_Rop_Region_2 (628) --------------------------------------------------

Dvv_Rop-2_v3 (192) --------------------------------------------------

1701 1750

Dvv_Rop_ORF (1698) GAAGAACTGGGAAGTCATCATCGGCTCGTCGCACATACTCACTCCCGAGG

Dvv_Rop_Region_1 (393) --------------------------------------------------

Dvv_Rop_Region_2 (628) --------------------------------------------------

Dvv_Rop-2_v3 (192) --------------------------------------------------

1751 1785

Dvv_Rop_ORF (1748) ACTTCCTAAGCAATCTGGCAACGTTGGCCGGCTAG

Dvv_Rop_Region_1 (393) -----------------------------------

Dvv_Rop_Region_2 (628) -----------------------------------

Dvv_Rop-2_v3 (192) -----------------------------------

**Sequence 2:** Dre4 Open Reading Frame (ORF), Dre4 Region 1, Dre4 Region 1 v1 and v2 alignment. Blue sequence is region red sequence is version. Sequences are reverse complement.

1 50

Dvv_Dre4_ORF (1) TCACTTTCTAGATTTCTTCGAGTGGTGTTTGTCACCGCTGGGACTTCTTT

Dvv_Dre4_Region_1 (1) --------------------------------------------------

Dvv_Dre4-1_v1 (1) --------------------------------------------------

Dvv-Dre4-1_v2 (1) --------------------------------------------------

51 100

Dvv_Dre4_ORF (51) GTCTCTTTTTATCGCCAGAACTGCTGCTTTTGTGAGAAGACGAGCTCTTT

Dvv_Dre4_Region_1 (1) --------------------------------------------------

Dvv_Dre4-1_v1 (1) --------------------------------------------------

Dvv-Dre4-1_v2 (1) --------------------------------------------------

101 150

Dvv_Dre4_ORF (101) TTTCTGTCCTTGTCCTTATCTCTAGATGAGGATGAGTGGCTAGAAGATTT

Dvv_Dre4_Region_1 (1) --------------------------------------------------

Dvv_Dre4-1_v1 (1) --------------------------------------------------

Dvv-Dre4-1_v2 (1) --------------------------------------------------

151 200

Dvv_Dre4_ORF (151) ATGGTTCGATGATGAGTGTCTATCTTTGTCCCTACTTTTTGAACTGCTGT

Dvv_Dre4_Region_1 (1) --------------------------------------------------

Dvv_Dre4-1_v1 (1) --------------------------------------------------

Dvv-Dre4-1_v2 (1) --------------------------------------------------

201 250

Dvv_Dre4_ORF (201) TTCGGTCCTTATCTTTTGAAGGTGAATGTTTACTACTGCTTGAAGAGGAG

Dvv_Dre4_Region_1 (1) --------------------------------------------------

Dvv_Dre4-1_v1 (1) --------------------------------------------------

Dvv-Dre4-1_v2 (1) --------------------------------------------------

251 300

Dvv_Dre4_ORF (251) TTATGTTTGCTAGGGCTCTTATGACTGCTACTGCTATGTCTGCTTTTATC

Dvv_Dre4_Region_1 (1) --------------------------------------------------

Dvv_Dre4-1_v1 (1) --------------------------------------------------

Dvv-Dre4-1_v2 (1) --------------------------------------------------

301 350

Dvv_Dre4_ORF (301) TTTTGATGATTTGCCACCTTTGGATGAAAATTTATCATGACCCTTGCTGC

Dvv_Dre4_Region_1 (1) --------------------------------------------------

Dvv_Dre4-1_v1 (1) --------------------------------------------------

Dvv-Dre4-1_v2 (1) --------------------------------------------------

351 400

Dvv_Dre4_ORF (351) GATGTTTATTTCTTCTGTCACCTTCGTCTTCAGCAGTATAAACTTTTTCT

Dvv_Dre4_Region_1 (1) --------------------------------------------------

Dvv_Dre4-1_v1 (1) --------------------------------------------------

Dvv-Dre4-1_v2 (1) --------------------------------------------------

401 450

Dvv_Dre4_ORF (401) CTATCCTCTTCTTCCGCTTCACGTTCAAGATCAGACCAGTCTTTTCCCGA

Dvv_Dre4_Region_1 (1) --------------------------------------------------

Dvv_Dre4-1_v1 (1) --------------------------------------------------

Dvv-Dre4-1_v2 (1) --------------------------------------------------

451 500

Dvv_Dre4_ORF (451) TTCTTCGTCAGATCCTAGATCTTCAGAAGCATCTTCATCATCGTCATCAT

Dvv_Dre4_Region_1 (1) --------------------------------------------------

Dvv_Dre4-1_v1 (1) --------------------------------------------------

Dvv-Dre4-1_v2 (1) --------------------------------------------------

501 550

Dvv_Dre4_ORF (501) CTTCAGTATCACCTTCCGAATATTCAGAGTCTTCTTCGCTCCAATCAGAT

Dvv_Dre4_Region_1 (1) --------------------------------------------------

Dvv_Dre4-1_v1 (1) --------------------------------------------------

Dvv-Dre4-1_v2 (1) --------------------------------------------------

551 600

Dvv_Dre4_ORF (551) CCTACTTCTTCTAAATCAGACGGTTGATACGCTTCATCTTCTTCTTCTGA

Dvv_Dre4_Region_1 (1) --------------------------------------------------

Dvv_Dre4-1_v1 (1) --------------------------------------------------

Dvv-Dre4-1_v2 (1) --------------------------------------------------

601 650

Dvv_Dre4_ORF (601) TTCTTCTTCTTTAGCTTGTTCTTCGTCAGATTCTGGGTCAAGGAAAGTCC

Dvv_Dre4_Region_1 (1) --------------------------------------------------

Dvv_Dre4-1_v1 (1) --------------------------------------------------

Dvv-Dre4-1_v2 (1) --------------------------------------------------

651 700

Dvv_Dre4_ORF (651) AACCACCACTGTCAAAGAAACCTTCTGGATCATCTGTAATGGTTTTCATA

Dvv_Dre4_Region_1 (1) --------------------------------------------------

Dvv_Dre4-1_v1 (1) --------------------------------------------------

Dvv-Dre4-1_v2 (1) --------------------------------------------------

701 750

Dvv_Dre4_ORF (701) ATTTTGACCCAATTAAGAGACTGAACTCCTTCAGAATATCTAATGTCACA

Dvv_Dre4_Region_1 (1) --------------------------------------------------

Dvv_Dre4-1_v1 (1) --------------------------------------------------

Dvv-Dre4-1_v2 (1) --------------------------------------------------

751 800

Dvv_Dre4_ORF (751) AGAATTTAACCATTCTTTTACATGATCCAGCATATTCATTGGTATAGCAT

Dvv_Dre4_Region_1 (1) --------------------------------------------------

Dvv_Dre4-1_v1 (1) --------------------------------------------------

Dvv-Dre4-1_v2 (1) --------------------------------------------------

801 850

Dvv_Dre4_ORF (801) TTACCATGACAGTCTTTTTAAGATACTCCTTAAAAACAAACACCATATCG

Dvv_Dre4_Region_1 (1) --------------------------------------------------

Dvv_Dre4-1_v1 (1) --------------------------------------------------

Dvv-Dre4-1_v2 (1) --------------------------------------------------

851 900

Dvv_Dre4_ORF (851) AAGTTCTTCAGGTGGAATTGTACACGTTCAAAATGTACTAACTCTACATC

Dvv_Dre4_Region_1 (1) --------------------------------------------------

Dvv_Dre4-1_v1 (1) --------------------------------------------------

Dvv-Dre4-1_v2 (1) --------------------------------------------------

901 950

Dvv_Dre4_ORF (901) TTCTAGGGTGACAACAAATGGAGGCCATTCAGTCAGGTTAACCAAACAAC

Dvv_Dre4_Region_1 (1) --------------------------------------------------

Dvv_Dre4-1_v1 (1) --------------------------------------------------

Dvv-Dre4-1_v2 (1) --------------------------------------------------

951 1000

Dvv_Dre4_ORF (951) CAGATGTAGGCTGGAGAAGGACTGTAGAGCGGAAGGGGGCTCCTGGAAAT

Dvv_Dre4_Region_1 (1) --------------------------------------------------

Dvv_Dre4-1_v1 (1) --------------------------------------------------

Dvv-Dre4-1_v2 (1) --------------------------------------------------

1001 1050

Dvv_Dre4_ORF (1001) CCCAACTCCCGGAATGGTGTATCAAATTCAATTTCTTGTTTTGTCATAGA

Dvv_Dre4_Region_1 (1) --------------------------------------------------

Dvv_Dre4-1_v1 (1) --------------------------------------------------

Dvv-Dre4-1_v2 (1) --------------------------------------------------

1051 1100

Dvv_Dre4_ORF (1051) TTCAACCTTTTCACAAAAACTTTTGAATGCAGTTTTCAATTTTTGTCTGA

Dvv_Dre4_Region_1 (1) --------------------------------------------------

Dvv_Dre4-1_v1 (1) --------------------------------------------------

Dvv-Dre4-1_v2 (1) --------------------------------------------------

1101 1150

Dvv_Dre4_ORF (1101) GCTCTCTTTCCGACTGCTCAGCAGCCAGATCATCTCGATCGTGCATGTGT

Dvv_Dre4_Region_1 (1) --------------------------------------------------

Dvv_Dre4-1_v1 (1) --------------------------------------------------

Dvv-Dre4-1_v2 (1) --------------------------------------------------

1151 1200

Dvv_Dre4_ORF (1151) TGATGTTTGCCCAAATCTGTGGTAATTTCTCCGACTTCCGTATAGAATTG

Dvv_Dre4_Region_1 (1) --------------------------------------------------

Dvv_Dre4-1_v1 (1) --------------------------------------------------

Dvv-Dre4-1_v2 (1) --------------------------------------------------

1201 1250

Dvv_Dre4_ORF (1201) AACGTCAACATGTTTCTTTTTTCCGAACATGATAGCATGTTTTAGGTGAA

Dvv_Dre4_Region_1 (1) --------------------------------------------------

Dvv_Dre4-1_v1 (1) --------------------------------------------------

Dvv-Dre4-1_v2 (1) --------------------------------------------------

1251 1300

Dvv_Dre4_ORF (1251) AATGTAACAGAATTATCATTTCTCCATCACACGGTTGAAAGAAGGCATTC

Dvv_Dre4_Region_1 (1) --------------------------------------------------

Dvv_Dre4-1_v1 (1) --------------------------------------------------

Dvv-Dre4-1_v2 (1) --------------------------------------------------

1301 1350

Dvv_Dre4_ORF (1301) TTGATGTTGTTGTAAAGAATGTCTACTTTATCACCCCTCACAGAAGTATA

Dvv_Dre4_Region_1 (1) --------------------------------------------------

Dvv_Dre4-1_v1 (1) --------------------------------------------------

Dvv-Dre4-1_v2 (1) --------------------------------------------------

1351 1400

Dvv_Dre4_ORF (1351) CCTAAAACCATTAGTGTGAGCTTCTAATGATCCTGTCATACGTTTTGAAA

Dvv_Dre4_Region_1 (1) --------------------------------------------------

Dvv_Dre4-1_v1 (1) --------------------------------------------------

Dvv-Dre4-1_v2 (1) --------------------------------------------------

1401 1450

Dvv_Dre4_ORF (1401) CAATATTTGGCCTAATATACAGATCTTTTAATTTAGGATTTCCTTTATTC

Dvv_Dre4_Region_1 (1) --------------------------------------------------

Dvv_Dre4-1_v1 (1) --------------------------------------------------

Dvv-Dre4-1_v2 (1) --------------------------------------------------

1451 1500

Dvv_Dre4_ORF (1451) TGTGAAAGTACTAATGTGTCCTGTTTAACCAAATCCTCTTTTTCTTTTTC

Dvv_Dre4_Region_1 (1) --------------------------------------------------

Dvv_Dre4-1_v1 (1) --------------------------------------------------

Dvv-Dre4-1_v2 (1) --------------------------------------------------

1501 1550

Dvv_Dre4_ORF (1501) TTCAGCTTCTCGTGTCTTAAATTTCCTTTGAACTTCTTTAATTAGACGAA

Dvv_Dre4_Region_1 (1) --------------------------------------------------

Dvv_Dre4-1_v1 (1) --------------------------------------------------

Dvv-Dre4-1_v2 (1) --------------------------------------------------

1551 1600

Dvv_Dre4_ORF (1551) AGGCTGTATTAAGGTTAGACGAGGGAGCAGTTACTACTCCTGGTTCTTTT

Dvv_Dre4_Region_1 (1) --------------------------------------------------

Dvv_Dre4-1_v1 (1) --------------------------------------------------

Dvv-Dre4-1_v2 (1) --------------------------------------------------

1601 1650

Dvv_Dre4_ORF (1601) GTATTGGTACTCCTATATGTTAATTCTTTAAGGAAAGTTGCTTCAGGCTG

Dvv_Dre4_Region_1 (1) --------------------------------------------------

Dvv_Dre4-1_v1 (1) --------------------------------------------------

Dvv-Dre4-1_v2 (1) --------------------------------------------------

1651 1700

Dvv_Dre4_ORF (1651) AGACCAGACATTACCTTCATTTCTGCCCATAGTTGTACCGGGATGGAAAA

Dvv_Dre4_Region_1 (1) --------------CTTCATTTCTGCCCATAGTTGTACCGGGATGGAAAA

Dvv_Dre4-1_v1 (1) --------------------------------------------------

Dvv-Dre4-1_v2 (1) --------------------------------------------------

1701 1750

Dvv_Dre4_ORF (1701) AGTTAATACGCAAATAGGTGTAGTCTCCCTCAATAGATTGAGAAATATTT

Dvv_Dre4_Region_1 (37) AGTTAATACGCAAATAGGTGTAGTCTCCCTCAATAGATTGAGAAATATTT

Dvv_Dre4-1_v1 (1) --------------------------------------------------

Dvv-Dre4-1_v2 (1) --------------------------------------------------

1751 1800

Dvv_Dre4_ORF (1751) TTGATTGTAGAAATATGGAAAGGTACCGCAATTCCATAAATAGGTAAAAT

Dvv_Dre4_Region_1 (87) TTGATTGTAGAAATATGGAAAGGTACCGCAATTCCATAAATAGGTAAAAT

Dvv_Dre4-1_v1 (1) -------------------------------------------------T

Dvv-Dre4-1_v2 (1) --------------------------------------------------

1801 1850

Dvv_Dre4_ORF (1801) AACAGTTTCATACTTCTGGTCCACATATAATTTTAGTTCTTTTACCTCGG

Dvv_Dre4_Region_1 (137) AACAGTTTCATACTTCTGGTCCACATATAATTTTAGTTCTTTTACCTCGG

Dvv_Dre4-1_v1 (2) AACAGTTTCATACTTCTGGTCCACATATAATTTTAGTTCTTTTACCTCGG

Dvv-Dre4-1_v2 (1) ------------------------------TTTTAGTTCTTTTACCTCGG

1851 1900

Dvv_Dre4_ORF (1851) GAACTCTAGGCATTTGATTGACACTTTTGTATGACACAGTATTTTTTCTC

Dvv_Dre4_Region_1 (187) GAACTCTAGGCATTTGATTGACACTTTTGTATGACACAGTATTTTTTCTC

Dvv_Dre4-1_v1 (52) GAACTCTAGGCATTTGATTGACACTTTTGTATGACACAGTATTTTTTCTC

Dvv-Dre4-1_v2 (21) GAACTCTAGGCATTTGATTGACACTTTTGTATGACACAGTATTTTTTCTC

1901 1950

Dvv_Dre4_ORF (1901) ACTTTCTCTTTATCTTTAGATCCAGATTGTTTTGCCAAACGCTCTTTTGC

Dvv_Dre4_Region_1 (237) ACTTTCTCTTTATCTTTAGATCCAGATTGTTTTGCCAAACGCTCTTTTGC

Dvv_Dre4-1_v1 (102) ACTTTCTCTTTATCTTTAGATCCAGATTGTTTTGCCAAACGCTCTTTTGC

Dvv-Dre4-1_v2 (71) ACTTTCTCTTTATCTTTAGATCCAGATTGTTTTGCCAAACGCTCTTTTGC

1951 2000

Dvv_Dre4_ORF (1951) TTTTTCATTTAGTTGAGCTGCCAATTCTTTCTGGTGCTCTTTTCTTTTTT

Dvv_Dre4_Region_1 (287) TTTTTCATTTAGTTGAGCTGCCAATTCTTTCTGGTGCTCTTTTCTTTTTT

Dvv_Dre4-1_v1 (152) TTTTTCATTTAGTTGAGCTGCCAA--------------------------

Dvv-Dre4-1_v2 (121) TTTTTCA-------------------------------------------

2001 2050

Dvv_Dre4_ORF (2001) CTTCGGTGCTGTGTTCAGTACGCAGTTTTGATTCAATAACAGCAGTTCTT

Dvv_Dre4_Region_1 (337) CTTCGGTGCTGTGTTCAGTACGCAGTTTTGATTCAATAACAGCAGTTCTT

Dvv_Dre4-1_v1 (176) --------------------------------------------------

Dvv-Dre4-1_v2 (128) --------------------------------------------------

2051 2100

Dvv_Dre4_ORF (2051) TTACCCCTTCCCAAAATTTCGGTCTTTTTCGGAGTGTTTTCTTTTTCCTC

Dvv_Dre4_Region_1 (387) TTACCCCTTCCCAAAATTTCGGTCTTTTTCGGAGTGTTTTCTTTTTCCTC

Dvv_Dre4-1_v1 (176) --------------------------------------------------

Dvv-Dre4-1_v2 (128) --------------------------------------------------

2101 2150

Dvv_Dre4_ORF (2101) ATCATTTTCTTCTGCGTCAGAATCATCTTTAAGGAAAATACCGATGTTCT

Dvv_Dre4_Region_1 (437) ATCATTTTCTTCTGCGTCAGAAT---------------------------

Dvv_Dre4-1_v1 (176) --------------------------------------------------

Dvv-Dre4-1_v2 (128) --------------------------------------------------

2151 2200

Dvv_Dre4_ORF (2151) TAATCTTCTTCTTGGATGGTGTTAAGACACTTGCTGGCTGACCTTCATTA

Dvv_Dre4_Region_1 (460) --------------------------------------------------

Dvv_Dre4-1_v1 (176) --------------------------------------------------

Dvv-Dre4-1_v2 (128) --------------------------------------------------

2201 2250

Dvv_Dre4_ORF (2201) ACCAAAACAGTATCTCCAATGAATAAAGCATATATTTTTCCCTCCTTGTC

Dvv_Dre4_Region_1 (460) --------------------------------------------------

Dvv_Dre4-1_v1 (176) --------------------------------------------------

Dvv-Dre4-1_v2 (128) --------------------------------------------------

2251 2300

Dvv_Dre4_ORF (2251) AGAAGCTTCCTTATTTTCCAAATTAGAAAAACCTATATTTAAATTGAACA

Dvv_Dre4_Region_1 (460) --------------------------------------------------

Dvv_Dre4-1_v1 (176) --------------------------------------------------

Dvv-Dre4-1_v2 (128) --------------------------------------------------

2301 2350

Dvv_Dre4_ORF (2301) CCATCCCCTTTTTGGCTTCAACTGTAGTTTTTGGTCCAATAATCAGGGAA

Dvv_Dre4_Region_1 (460) --------------------------------------------------

Dvv_Dre4-1_v1 (176) --------------------------------------------------

Dvv-Dre4-1_v2 (128) --------------------------------------------------

2351 2400

Dvv_Dre4_ORF (2351) TTTTCTTTGAATTCTATCCCCATTGCGAATCCAAAATTTTTGGTCAAATG

Dvv_Dre4_Region_1 (460) --------------------------------------------------

Dvv_Dre4-1_v1 (176) --------------------------------------------------

Dvv-Dre4-1_v2 (128) --------------------------------------------------

2401 2450

Dvv_Dre4_ORF (2401) GTCAGCTAAATTTGGCTTTTCCTTTTTAACCATATTGTAACCAGTGTCAT

Dvv_Dre4_Region_1 (460) --------------------------------------------------

Dvv_Dre4-1_v1 (176) --------------------------------------------------

Dvv-Dre4-1_v2 (128) --------------------------------------------------

2451 2500

Dvv_Dre4_ORF (2451) AAACTTCCGACAATTTTACTCCATTCTGAAGCACTTTTATTAACTCCTCT

Dvv_Dre4_Region_1 (460) --------------------------------------------------

Dvv_Dre4-1_v1 (176) --------------------------------------------------

Dvv-Dre4-1_v2 (128) --------------------------------------------------

2501 2550

Dvv_Dre4_ORF (2501) TCAATTGTTAAAAGAAAATTATAGTTGTTTTGGATTTCCTCTGTGGGGTT

Dvv_Dre4_Region_1 (460) --------------------------------------------------

Dvv_Dre4-1_v1 (176) --------------------------------------------------

Dvv-Dre4-1_v2 (128) --------------------------------------------------

2551 2600

Dvv_Dre4_ORF (2551) TACTAACAAGGTTCGGACGATGTTGGAACAGTATGACTTATAGCGAGCGC

Dvv_Dre4_Region_1 (460) --------------------------------------------------

Dvv_Dre4-1_v1 (176) --------------------------------------------------

Dvv-Dre4-1_v2 (128) --------------------------------------------------

2601 2650

Dvv_Dre4_ORF (2601) CTAGAGAACAAACAATGGCTCCAAAATGTAGTGAATTTTTATCACTAATA

Dvv_Dre4_Region_1 (460) --------------------------------------------------

Dvv_Dre4-1_v1 (176) --------------------------------------------------

Dvv-Dre4-1_v2 (128) --------------------------------------------------

2651 2700

Dvv_Dre4_ORF (2651) ACACTGAATTTTAAATTGTAAGTGCCTCCAGACTGTATAATGGCAGGGTA

Dvv_Dre4_Region_1 (460) --------------------------------------------------

Dvv_Dre4-1_v1 (176) --------------------------------------------------

Dvv-Dre4-1_v2 (128) --------------------------------------------------

2701 2750

Dvv_Dre4_ORF (2701) GCACATATCAACTTGATTAACATCAACCCCTGAGACATATTTTTTATCAC

Dvv_Dre4_Region_1 (460) --------------------------------------------------

Dvv_Dre4-1_v1 (176) --------------------------------------------------

Dvv-Dre4-1_v2 (128) --------------------------------------------------

2751 2800

Dvv_Dre4_ORF (2751) TTATGGCAGATTCAACTCCTTCTGCTAGTTTGCTATGCTTGACTTTCTTT

Dvv_Dre4_Region_1 (460) --------------------------------------------------

Dvv_Dre4-1_v1 (176) --------------------------------------------------

Dvv-Dre4-1_v2 (128) --------------------------------------------------

2801 2850

Dvv_Dre4_ORF (2801) TCCGAATCAATAATCTCCATAATCTGATCTTTTAAATACTTTGTGAATAC

Dvv_Dre4_Region_1 (460) --------------------------------------------------

Dvv_Dre4-1_v1 (176) --------------------------------------------------

Dvv-Dre4-1_v2 (128) --------------------------------------------------

2851 2900

Dvv_Dre4_ORF (2851) ATCTACAGTTACCTGAGATGCTTTTTTCATGGTAGTAATTTCAGATTCTT

Dvv_Dre4_Region_1 (460) --------------------------------------------------

Dvv_Dre4-1_v1 (176) --------------------------------------------------

Dvv-Dre4-1_v2 (128) --------------------------------------------------

2901 2950

Dvv_Dre4_ORF (2901) CTTTAGGGCTAATGAGGTAAGCAATCTGAGAACTCATATCCGCAGTTTGA

Dvv_Dre4_Region_1 (460) --------------------------------------------------

Dvv_Dre4-1_v1 (176) --------------------------------------------------

Dvv-Dre4-1_v2 (128) --------------------------------------------------

2951 3000

Dvv_Dre4_ORF (2951) AATTCTTGTTTCTTGAGCATTGCTTTCCAATTATCAACAAAAGATCCAGA

Dvv_Dre4_Region_1 (460) --------------------------------------------------

Dvv_Dre4-1_v1 (176) --------------------------------------------------

Dvv-Dre4-1_v2 (128) --------------------------------------------------

3001 3050

Dvv_Dre4_ORF (3001) ATAATTATCCTTTGCAAAAACCCCCAAAGTTTTACCATTTTTGCTCTCCT

Dvv_Dre4_Region_1 (460) --------------------------------------------------

Dvv_Dre4-1_v1 (176) --------------------------------------------------

Dvv-Dre4-1_v2 (128) --------------------------------------------------

3051 3100

Dvv_Dre4_ORF (3051) TTATAGCTTCACAAAGAATTTTAAATTTTGCTTCGTCGCTGTCTTTATCG

Dvv_Dre4_Region_1 (460) --------------------------------------------------

Dvv_Dre4-1_v1 (176) --------------------------------------------------

Dvv-Dre4-1_v2 (128) --------------------------------------------------

3101 3150

Dvv_Dre4_ORF (3101) CGGACCAACAAGTTTAGTTGGATTTGGTTCTCATCCTTCTCTTTAAGTTC

Dvv_Dre4_Region_1 (460) --------------------------------------------------

Dvv_Dre4-1_v1 (176) --------------------------------------------------

Dvv-Dre4-1_v2 (128) --------------------------------------------------

3151 3200

Dvv_Dre4_ORF (3151) GGCTTGCCGAAGAAATTCAACCTTCTTTTTACTGGTTAAGAAATAAGCTT

Dvv_Dre4_Region_1 (460) --------------------------------------------------

Dvv_Dre4-1_v1 (176) --------------------------------------------------

Dvv-Dre4-1_v2 (128) --------------------------------------------------

3201 3250

Dvv_Dre4_ORF (3201) TTTTCTCTACAAGAACCATAATAGTATCTGTAAGTTCGTAGCCCAACAGC

Dvv_Dre4_Region_1 (460) --------------------------------------------------

Dvv_Dre4-1_v1 (176) --------------------------------------------------

Dvv-Dre4-1_v2 (128) --------------------------------------------------

3251 3300

Dvv_Dre4_ORF (3251) CATATTTGCAATGCACCTGATTTGCTGTACACAATATCATCGTCTTTGCC

Dvv_Dre4_Region_1 (460) --------------------------------------------------

Dvv_Dre4-1_v1 (176) --------------------------------------------------

Dvv-Dre4-1_v2 (128) --------------------------------------------------

3301 3350

Dvv_Dre4_ORF (3301) GACTGCAGTGACTAAAGCATCCATTTTTGAGAAACCATTTTCTCCATCGG

Dvv_Dre4_Region_1 (460) --------------------------------------------------

Dvv_Dre4-1_v1 (176) --------------------------------------------------

Dvv-Dre4-1_v2 (128) --------------------------------------------------

3351 3400

Dvv_Dre4_ORF (3351) TATTTTGCCATGAATTGTAGAGCTTCTTTAATCTACGGTGAAAAGACTGT

Dvv_Dre4_Region_1 (460) --------------------------------------------------

Dvv_Dre4-1_v1 (176) --------------------------------------------------

Dvv-Dre4-1_v2 (128) --------------------------------------------------

3401 3423

Dvv_Dre4_ORF (3401) TTGTCTAAATTGACATTAGCCAT

Dvv_Dre4_Region_1 (460) -----------------------

Dvv_Dre4-1_v1 (176) -----------------------

Dvv-Dre4-1_v2 (128) -----------------------

**Sequence 3:** *RpII140* Open Reading Frame (ORF), *RpII140 Region 1*, *RpII140 Region 1 v1* and *v2* alignment. Blue sequence is region red sequence is version.

1 50

Dvv_RpII140_ORF (1) ATGTATGATCAAGAAGATGATCAATTTGACGATGAAGATACAGAAGAAAT

Dvv_RpII140-1 (1) --------------------------------------------------

Dvv_RpII140_v1 (1) --------------------------------------------------

Dvv_RpII140_v2 (1) --------------------------------------------------

51 100

Dvv_RpII140_ORF (51) CAGTGGAGAACTCTGGCAGGAAGCCTGCTGGATTGTTATTAATGCCTACT

Dvv_RpII140-1 (1) --------------------------------------------------

Dvv_RpII140_v1 (1) --------------------------------------------------

Dvv_RpII140_v2 (1) --------------------------------------------------

101 150

Dvv_RpII140_ORF (101) TTGATGAAAAAGGTTTGGTACGGCAGCAGTTGGATAGTTTTGATGAATTC

Dvv_RpII140-1 (1) --------------------------------------------------

Dvv_RpII140_v1 (1) --------------------------------------------------

Dvv_RpII140_v2 (1) --------------------------------------------------

151 200

Dvv_RpII140_ORF (151) ATTCAAATGTCTGTACAGAGGATCGTTGAAGATTCCCCACAGATAGATCT

Dvv_RpII140-1 (1) --------------------------------------------------

Dvv_RpII140_v1 (1) --------------------------------------------------

Dvv_RpII140_v2 (1) --------------------------------------------------

201 250

Dvv_RpII140_ORF (201) ACAAGCAGAAGCGCAGCATACATCTGGGGAAATCGAAACCCCACCTCGAT

Dvv_RpII140-1 (1) --------------------------------------------------

Dvv_RpII140_v1 (1) --------------------------------------------------

Dvv_RpII140_v2 (1) --------------------------------------------------

251 300

Dvv_RpII140_ORF (251) ATTTGCTGAAGTTTGAACAGATCTATCTTTCAAAACCTACCCATTGGGAG

Dvv_RpII140-1 (1) -----------------------------------CCTACCCATTGGGAG

Dvv_RpII140_v1 (1) --------------------------------------------------

Dvv_RpII140_v2 (1) --------------------------------------------------

301 350

Dvv_RpII140_ORF (301) AAAGACGGTGCCCCATCTCCTATGATGCCTAATGAAGCTAGATTAAGAAA

Dvv_RpII140-1 (16) AAAGACGGTGCCCCATCTCCTATGATGCCTAATGAAGCTAGATTAAGAAA

Dvv_RpII140_v1 (1) --------------------------------------------------

Dvv_RpII140_v2 (1) --------------------------------------------------

351 400

Dvv_RpII140_ORF (351) TTTGACTTATTCTGCTCCTCTTTATGTAGATATAACAAAAACAATTGTGA

Dvv_RpII140-1 (66) TTTGACTTATTCTGCTCCTCTTTATGTAGATATAACAAAAACAATTGTGA

Dvv_RpII140_v1 (1) --------------------------------------------------

Dvv_RpII140_v2 (1) --------------------------------------------------

401 450

Dvv_RpII140_ORF (401) AAGAAGGAGAGGATCCTATAGAAACTCAACATCAGAAAACTTTTATAGGT

Dvv_RpII140-1 (116) AAGAAGGAGAGGATCCTATAGAAACTCAACATCAGAAAACTTTTATAGGT

Dvv_RpII140_v1 (1) --------------------------------------------------

Dvv_RpII140_v2 (1) --------------------------------------------------

451 500

Dvv_RpII140_ORF (451) AAAATTCCCATTATGTTGAGGTCAACATACTGTCTGCTCAGTGGATTAAC

Dvv_RpII140-1 (166) AAAATTCCCATTATGTTGAGGTCAACATACTGTCTGCTCAGTGGATTAAC

Dvv_RpII140_v1 (1) --------------------------------------------------

Dvv_RpII140_v2 (1) --------------------------------------------------

501 550

Dvv_RpII140_ORF (501) AGATCGTGATTTAACAGAATTAAACGAGTGTCCCTTAGATCCTGGCGGAT

Dvv_RpII140-1 (216) AGATCGTGATTTAACAGAATTAAACGAGTGTCCCTTAGATCCTGGCGGAT

Dvv_RpII140_v1 (1) --------------------------------------------------

Dvv_RpII140_v2 (1) --------------------------------------------------

551 600

Dvv_RpII140_ORF (551) ATTTCATAATTAACGGTTCTGAAAAAGTATTAATTGCTCAAGAGAAGATG

Dvv_RpII140-1 (266) ATTTCATAATTAACGGTTCTGAAAAAGTATTAATTGCTCAAGAGAAGATG

Dvv_RpII140_v1 (1) --------------------------------------------------

Dvv_RpII140_v2 (1) --------------------------------------------------

601 650

Dvv_RpII140_ORF (601) GCAACTAACACAGTATATGTATTTTCAATGAAAGACGGAAAATATGCGTA

Dvv_RpII140-1 (316) GCAACTAACACAGTATATGTATTTTCAATGAAAGACGGAAAATATGCGTA

Dvv_RpII140_v1 (1) --------------------------------------------------

Dvv_RpII140_v2 (1) --------------------------------------------------

651 700

Dvv_RpII140_ORF (651) CAAATCTGAAATAAGATCTTGTCTTGAGCACAGCTCTCGGCCAACATCAA

Dvv_RpII140-1 (366) CAAATCTGAAATAAGATCTTGTCTTGAGCACAGCTCTCGGCCAACATCAA

Dvv_RpII140_v1 (1) --------------------------------------------------

Dvv_RpII140_v2 (1) --------------------------------------------------

701 750

Dvv_RpII140_ORF (701) CTCTGTGGGTAAATATGATGGCTCGTGGTGGCCAGGCCATCAAAAAAGCT

Dvv_RpII140-1 (416) CTCTGTGGGTAAATATGATGGCTCGTGGTGGCCAGGCCATCAAAAAAGCT

Dvv_RpII140_v1 (1) --------------------------------------------------

Dvv_RpII140_v2 (1) --------------------------------------------------

751 800

Dvv_RpII140_ORF (751) GCTATTGGTCAACGTATTATAGCTATTCTTCCATACATCAAACAAGAGAT

Dvv_RpII140-1 (466) GCT-----------------------------------------------

Dvv_RpII140_v1 (1) --------------------------------------------------

Dvv_RpII140_v2 (1) --------------------------------------------------

801 850

Dvv_RpII140_ORF (801) CCCTATAATGATAGTTTTCAGAGCTTTAGGGTTTGTAGCAGACAGAGATA

Dvv_RpII140-1 (469) --------------------------------------------------

Dvv_RpII140_v1 (1) --------------------------------------------------

Dvv_RpII140_v2 (1) --------------------------------------------------

851 900

Dvv_RpII140_ORF (851) TTCTAGAACACATTATTTACGATTTCGACGATCCAGAATTGATGGAAATG

Dvv_RpII140-1 (469) --------------------------------------------------

Dvv_RpII140_v1 (1) --------------------------------------------------

Dvv_RpII140_v2 (1) --------------------------------------------------

901 950

Dvv_RpII140_ORF (901) GTAAAACCTTCGTTAGATGAAGCTTTCGTAATTCAGGAACAGAATATTGC

Dvv_RpII140-1 (469) --------------------------------------------------

Dvv_RpII140_v1 (1) --------------------------------------------------

Dvv_RpII140_v2 (1) --------------------------------------------------

951 1000

Dvv_RpII140_ORF (951) TTTGAATTTCATCGGAGCGAGAGGTGCCAGGCCGGGAGTAACTAAAGAAA

Dvv_RpII140-1 (469) --------------------------------------------------

Dvv_RpII140_v1 (1) --------------------------------------------------

Dvv_RpII140_v2 (1) --------------------------------------------------

1001 1050

Dvv_RpII140_ORF (1001) AGCGTGTGAAATATGCTAGAGAAATTTTACAAAAGGAAATGTTGCCTCAT

Dvv_RpII140-1 (469) --------------------------------------------------

Dvv_RpII140_v1 (1) --------------------------------------------------

Dvv_RpII140_v2 (1) --------------------------------------------------

1051 1100

Dvv_RpII140_ORF (1051) GTTGGTGTATCCGATTTTTGTGAGACTAAAAAAGCTTATTTTCTTGGATA

Dvv_RpII140-1 (469) --------------------------------------------------

Dvv_RpII140_v1 (1) --------------------------------------------------

Dvv_RpII140_v2 (1) --------------------------------------------------

1101 1150

Dvv_RpII140_ORF (1101) TATGGTACATCGACTTTTGCTGGCTGCACTCGGACGTCGAGAGTTAGATG

Dvv_RpII140-1 (469) --------------------------------------------------

Dvv_RpII140_v1 (1) --------------------------------------------------

Dvv_RpII140_v2 (1) --------------------------------------------------

1151 1200

Dvv_RpII140_ORF (1151) ACAGAGATCACTACGGAAATAAGAGACTCGATTTGGCTGGACCATTATTG

Dvv_RpII140-1 (469) --------------------------------------------------

Dvv_RpII140_v1 (1) ----------------AAATAAGAGACTCGATTTGGCTGGACCATTATTG

Dvv_RpII140_v2 (1) --------------------------------------------------

1201 1250

Dvv_RpII140_ORF (1201) GCTTTCCTCTTCAGAGGGCTTTTCAAGAACCTAATGAAAGAAGTTCGTAT

Dvv_RpII140-1 (469) --------------------------------------------------

Dvv_RpII140_v1 (35) GCTTTCCTCTTCAGAGGGCTTTTCAAGAACCTAATGAAAGAAGTTCGTAT

Dvv_RpII140_v2 (1) --------------------------------------------------

1251 1300

Dvv_RpII140_ORF (1251) GTATGCCCAGAAGTTTATCGATAGAGGCAAAGATTTCAATCTGGATCTGG

Dvv_RpII140-1 (469) --------------------------------------------------

Dvv_RpII140_v1 (85) GTATGCCCAGAAGTTTATCGATAGAGGCAAAGATTTCAATCTGGATCTGG

Dvv_RpII140_v2 (1) --------------------------------------------------

1301 1350

Dvv_RpII140_ORF (1301) CCATCAAAACCAAACTAATAACGGACGGTCTGAGGTATTCTCTCGCTACT

Dvv_RpII140-1 (469) --------------------------------------------------

Dvv_RpII140_v1 (135) CCATCAAAACCAAACTAATAACGGACGGTCTGAGGTATTCTCTCGC----

Dvv_RpII140_v2 (1) --------------------------------------------------

1351 1400

Dvv_RpII140_ORF (1351) GGAAACTGGGGTGACCAGAAAAAAGCCCATCAAGCAAGAGCTGGCGTGTC

Dvv_RpII140-1 (469) --------------------------------------------------

Dvv_RpII140_v1 (181) --------------------------------------------------

Dvv_RpII140_v2 (1) --------------------------------------------------

1401 1450

Dvv_RpII140_ORF (1401) CCAGGTATTGAATCGTCTAACTTTCGCATCGACGTTGTCACATTTAAGGC

Dvv_RpII140-1 (469) --------------------------------------------------

Dvv_RpII140_v1 (181) --------------------------------------------------

Dvv_RpII140_v2 (1) --------------------------------------------------

1451 1500

Dvv_RpII140_ORF (1451) GTGTCAATTCTCCAATTGGACGAGACGGTAAACTAGCCAAGCCTCGACAG

Dvv_RpII140-1 (469) --------------------------------------------------

Dvv_RpII140_v1 (181) --------------------------------------------------

Dvv_RpII140_v2 (1) --------------------------------------------------

1501 1550

Dvv_RpII140_ORF (1501) TTGCATAATACGTTGTGGGGAATGATTTGCCCTGCTGAAACTCCAGAAGG

Dvv_RpII140-1 (469) --------------------------------------------------

Dvv_RpII140_v1 (181) --------------------------------------------------

Dvv_RpII140_v2 (1) --------------------------------------------------

1551 1600

Dvv_RpII140_ORF (1551) TGCTGCTGTAGGACTTGTAAAAAATCTTGCTCTGATGGCTTATATTTCCG

Dvv_RpII140-1 (469) --------------------------------------------------

Dvv_RpII140_v1 (181) --------------------------------------------------

Dvv_RpII140_v2 (1) --------------------------------------------------

1601 1650

Dvv_RpII140_ORF (1601) TCGGTTCCCAACCATCGCCAATTTTGGAGTTCTTGGAGGAGTGGTCCATG

Dvv_RpII140-1 (469) --------------------------------------------------

Dvv_RpII140_v1 (181) --------------------------------------------------

Dvv_RpII140_v2 (1) --------------------------------------------------

1651 1700

Dvv_RpII140_ORF (1651) GAAAACTTGGAAGAAATAGCGCCATCTGCAATTGCTAACGCTACCAAAAT

Dvv_RpII140-1 (469) --------------------------------------------------

Dvv_RpII140_v1 (181) --------------------------------------------------

Dvv_RpII140_v2 (1) --------------------------------------------------

1701 1750

Dvv_RpII140_ORF (1701) CTTTGTGAATGGTTGTTGGGTGGGAATTCACAGAGACCCTGAACAACTTA

Dvv_RpII140-1 (469) --------------------------------------------------

Dvv_RpII140_v1 (181) --------------------------------------------------

Dvv_RpII140_v2 (1) --------------------------------------------------

1751 1800

Dvv_RpII140_ORF (1751) TGGCGACACTGCGTAAACTTCGACGTCAAATGGACATCATCGTATCAGAA

Dvv_RpII140-1 (469) --------------------------------------------------

Dvv_RpII140_v1 (181) --------------------------------------------------

Dvv_RpII140_v2 (1) --------------------------------------------------

1801 1850

Dvv_RpII140_ORF (1801) GTATCGATGATTCGAGATATTCGTGACAGAGAAATAAGAATTTACACAGA

Dvv_RpII140-1 (469) --------------------------------------------------

Dvv_RpII140_v1 (181) --------------------------------------------------

Dvv_RpII140_v2 (1) --------------------------------------------------

1851 1900

Dvv_RpII140_ORF (1851) CGCCGGAAGAATCTGTAGACCTTTGTTAATCGTCGAAAATGGCCAGTTGC

Dvv_RpII140-1 (469) --------------------------------------------------

Dvv_RpII140_v1 (181) --------------------------------------------------

Dvv_RpII140_v2 (1) --------------------------------------------------

1901 1950

Dvv_RpII140_ORF (1901) TATTGAAGAAAAGACATATTGATATGTTAAAAGAAAGAGAATATAATAAT

Dvv_RpII140-1 (469) --------------------------------------------------

Dvv_RpII140_v1 (181) --------------------------------------------------

Dvv_RpII140_v2 (1) --------------------------------------------------

1951 2000

Dvv_RpII140_ORF (1951) TATGGTTGGCAGGTACTGGTCGCGTCTGGTGTGGTGGAGTACATAGACAC

Dvv_RpII140-1 (469) --------------------------------------------------

Dvv_RpII140_v1 (181) --------------------------------------------------

Dvv_RpII140_v2 (1) --------------------------------------------------

2001 2050

Dvv_RpII140_ORF (2001) CTTAGAAGAAGAAACCGTAATGATTGCCATGAACCCCGAAGATTTACGCC

Dvv_RpII140-1 (469) --------------------------------------------------

Dvv_RpII140_v1 (181) --------------------------------------------------

Dvv_RpII140_v2 (1) --------------------------------------------------

2051 2100

Dvv_RpII140_ORF (2051) AAGACAAAGAATGCGCTTATTGTACAACATACACTCATTGTGAAATTCAT

Dvv_RpII140-1 (469) --------------------------------------------------

Dvv_RpII140_v1 (181) --------------------------------------------------

Dvv_RpII140_v2 (1) --------------------------------------------------

2101 2150

Dvv_RpII140_ORF (2101) CCAGCCATGATTTTAGGAGTTTGCGCTTCCATCATTCCGTTCCCAGATCA

Dvv_RpII140-1 (469) --------------------------------------------------

Dvv_RpII140_v1 (181) --------------------------------------------------

Dvv_RpII140_v2 (1) --------------------------------------------------

2151 2200

Dvv_RpII140_ORF (2151) CAACCAGAGTCCCAGAAACACCTATCAAAGTGCTATGGGTAAACAAGCCA

Dvv_RpII140-1 (469) --------------------------------------------------

Dvv_RpII140_v1 (181) --------------------------------------------------

Dvv_RpII140_v2 (1) --------------------------------------------------

2201 2250

Dvv_RpII140_ORF (2201) TGGGTGTCTATATCACAAACTTCCACGTTAGAATGGACACACTGGCCCAC

Dvv_RpII140-1 (469) --------------------------------------------------

Dvv_RpII140_v1 (181) --------------------------------------------------

Dvv_RpII140_v2 (1) --------------------------------------------------

2251 2300

Dvv_RpII140_ORF (2251) GTCCTGTACTATCCACATAAACCTCTGGTAACGACAAGATCTATGGAGTA

Dvv_RpII140-1 (469) --------------------------------------------------

Dvv_RpII140_v1 (181) --------------------------------------------------

Dvv_RpII140_v2 (1) --------------------------------------------------

2301 2350

Dvv_RpII140_ORF (2301) TCTTAGATTCAGAGAGTTACCGGCTGGAATCAATAGTATCGTTGCGATTG

Dvv_RpII140-1 (469) --------------------------------------------------

Dvv_RpII140_v1 (181) --------------------------------------------------

Dvv_RpII140_v2 (1) --------------------------------------------------

2351 2400

Dvv_RpII140_ORF (2351) CTTGTTATACAGGATACAATCAGGAAGATTCTGTTATCTTGAATGCTTCT

Dvv_RpII140-1 (469) --------------------------------------------------

Dvv_RpII140_v1 (181) --------------------------------------------------

Dvv_RpII140_v2 (1) ----------------------------------------------TTCT

2401 2450

Dvv_RpII140_ORF (2401) GCAGTAGAAAGAGGATTTTTCAGATCTGTGTTTTACCGGTCTTATAAAGA

Dvv_RpII140-1 (469) --------------------------------------------------

Dvv_RpII140_v1 (181) --------------------------------------------------

Dvv_RpII140_v2 (5) GCAGTAGAAAGAGGATTTTTCAGATCTGTGTTTTACCGGTCTTATAAAGA

2451 2500

Dvv_RpII140_ORF (2451) CGCCGAATCCAAACGTATAGGAGACCAGGAAGAACAATTCGAAAAACCGA

Dvv_RpII140-1 (469) --------------------------------------------------

Dvv_RpII140_v1 (181) --------------------------------------------------

Dvv_RpII140_v2 (55) CGCCGAATCCAAACGTATAGGAGACCAGGAAGAACAATTCGAAAAACCGA

2501 2550

Dvv_RpII140_ORF (2501) CAAGACAGACGTGCCAGGGCATGAGGAATGCCCTTTACGATAAATTAGAC

Dvv_RpII140-1 (469) --------------------------------------------------

Dvv_RpII140_v1 (181) --------------------------------------------------

Dvv_RpII140_v2 (105) CAAGACAGACGTGCCAGGGCATGAGGAATGCCCTTTACGATAAATTAGAC

2551 2600

Dvv_RpII140_ORF (2551) GACGACGGACTGATATCTCCAGGTATTCGTGTGTCTGGAGACGATGTAGT

Dvv_RpII140-1 (469) --------------------------------------------------

Dvv_RpII140_v1 (181) --------------------------------------------------

Dvv_RpII140_v2 (155) GACGAC--------------------------------------------

2601 2650

Dvv_RpII140_ORF (2601) TATAGGAAAAACTATGACGCTACCAGAAACAGATGACGAGTTGGATGGAA

Dvv_RpII140-1 (469) --------------------------------------------------

Dvv_RpII140_v1 (181) --------------------------------------------------

Dvv_RpII140_v2 (161) --------------------------------------------------

2651 2700

Dvv_RpII140_ORF (2651) CTACAAAACGGTACTCCAAGAGAGACGCATCAACATTTTTGCGTAACAGT

Dvv_RpII140-1 (469) --------------------------------------------------

Dvv_RpII140_v1 (181) --------------------------------------------------

Dvv_RpII140_v2 (161) --------------------------------------------------

2701 2750

Dvv_RpII140_ORF (2701) GAAACTGGAGTTGTTGATCAAGTGATGTTAACGCTAAATTCAGAAGGATA

Dvv_RpII140-1 (469) --------------------------------------------------

Dvv_RpII140_v1 (181) --------------------------------------------------

Dvv_RpII140_v2 (161) --------------------------------------------------

2751 2800

Dvv_RpII140_ORF (2751) TAAGTTTTGTAAGATAAGGGTGAGATCTGTGAGGATACCACAAATTGGAG

Dvv_RpII140-1 (469) --------------------------------------------------

Dvv_RpII140_v1 (181) --------------------------------------------------

Dvv_RpII140_v2 (161) --------------------------------------------------

2801 2850

Dvv_RpII140_ORF (2801) ACAAGTTCGCGTCTAGGCACGGACAAAAGGGAACGTGCGGTATACAATAC

Dvv_RpII140-1 (469) --------------------------------------------------

Dvv_RpII140_v1 (181) --------------------------------------------------

Dvv_RpII140_v2 (161) --------------------------------------------------

2851 2900

Dvv_RpII140_ORF (2851) CGTCAAGAGGATATGATTTTTAGTGCTGAAGGTATTACTCCAGACATTAT

Dvv_RpII140-1 (469) --------------------------------------------------

Dvv_RpII140_v1 (181) --------------------------------------------------

Dvv_RpII140_v2 (161) --------------------------------------------------

2901 2950

Dvv_RpII140_ORF (2901) CATCAATCCTCACGCTATCCCCTCCCGTATGACGATTGGTCACTTGATCG

Dvv_RpII140-1 (469) --------------------------------------------------

Dvv_RpII140_v1 (181) --------------------------------------------------

Dvv_RpII140_v2 (161) --------------------------------------------------

2951 3000

Dvv_RpII140_ORF (2951) AGTGCATCCAAGGTAAGGTATCATCCAATAAAGGAGAAATCGGTGATGCT

Dvv_RpII140-1 (469) --------------------------------------------------

Dvv_RpII140_v1 (181) --------------------------------------------------

Dvv_RpII140_v2 (161) --------------------------------------------------

3001 3050

Dvv_RpII140_ORF (3001) ACACCTTTCAACGATGCCGTCAACGTACAGAAAATCTCCACTTTGTTACA

Dvv_RpII140-1 (469) --------------------------------------------------

Dvv_RpII140_v1 (181) --------------------------------------------------

Dvv_RpII140_v2 (161) --------------------------------------------------

3051 3100

Dvv_RpII140_ORF (3051) AGAATATGGTTATCAACTCAGAGGCAACGAAGTAATGTACAACGGCCACA

Dvv_RpII140-1 (469) --------------------------------------------------

Dvv_RpII140_v1 (181) --------------------------------------------------

Dvv_RpII140_v2 (161) --------------------------------------------------

3101 3150

Dvv_RpII140_ORF (3101) CTGGACGTAAGATAAATGCTCAGATTTTCTTAGGACCTACTTACTATCAA

Dvv_RpII140-1 (469) --------------------------------------------------

Dvv_RpII140_v1 (181) --------------------------------------------------

Dvv_RpII140_v2 (161) --------------------------------------------------

3151 3200

Dvv_RpII140_ORF (3151) AGATTGAAGCACATGGTGGACGATAAGATCCATTCAAGAGCTAGAGGACC

Dvv_RpII140-1 (469) --------------------------------------------------

Dvv_RpII140_v1 (181) --------------------------------------------------

Dvv_RpII140_v2 (161) --------------------------------------------------

3201 3250

Dvv_RpII140_ORF (3201) GTTGCAGATTCTCGTCAGACAGCCTATGGAGGGTCGTGCAAGAGACGGTG

Dvv_RpII140-1 (469) --------------------------------------------------

Dvv_RpII140_v1 (181) --------------------------------------------------

Dvv_RpII140_v2 (161) --------------------------------------------------

3251 3300

Dvv_RpII140_ORF (3251) GTCTACGTTTCGGTGAGATGGAACGAGATTGTCAAATTTCACACGGTGCT

Dvv_RpII140-1 (469) --------------------------------------------------

Dvv_RpII140_v1 (181) --------------------------------------------------

Dvv_RpII140_v2 (161) --------------------------------------------------

3301 3350

Dvv_RpII140_ORF (3301) GCACAATTCTTAAGAGAGAGGCTGTTTGAAGTTTCTGATCCTTATAGAAT

Dvv_RpII140-1 (469) --------------------------------------------------

Dvv_RpII140_v1 (181) --------------------------------------------------

Dvv_RpII140_v2 (161) --------------------------------------------------

3351 3400

Dvv_RpII140_ORF (3351) CCATATTTGCAACTTCTGTGGTTTGATAGCTATCGCCAATTTGCGTAACA

Dvv_RpII140-1 (469) --------------------------------------------------

Dvv_RpII140_v1 (181) --------------------------------------------------

Dvv_RpII140_v2 (161) --------------------------------------------------

3401 3450

Dvv_RpII140_ORF (3401) ACACCTTTGAATGTAAGGGTTGCAAAAATAAGACGCAGATATCGCAAGTT

Dvv_RpII140-1 (469) --------------------------------------------------

Dvv_RpII140_v1 (181) --------------------------------------------------

Dvv_RpII140_v2 (161) --------------------------------------------------

3451 3500

Dvv_RpII140_ORF (3451) AGACTGCCTTATGCTGCGAAATTACTGTTCCAAGAGCTAATGTCGATGAA

Dvv_RpII140-1 (469) --------------------------------------------------

Dvv_RpII140_v1 (181) --------------------------------------------------

Dvv_RpII140_v2 (161) --------------------------------------------------

3501 3528

Dvv_RpII140_ORF (3501) TATAGCGCCGAGACTTATGGTACTGTAG

Dvv_RpII140-1 (469) ----------------------------

Dvv_RpII140_v1 (181) ----------------------------

Dvv_RpII140_v2 (161) ----------------------------

**Sequence 4:** Ncm Open Reading Frame (ORF), Ncm Region 1, Ncm Region 2, Ncm Region 1 v1 and v2 alignment. Blue sequence is region red sequence is version. Sequence is reverse complement.

1 50

Dvv_Ncm_ORF (1) TTAGCTCCTTCTTCTTTCTCTGCTTCTACGTCTACGCTCTTCCTTGACTT

Dvv_Ncm_Region_2 (1) --------------------------------------------------

Dvv_Ncm_Region_1 (1) --------------------------------------------------

Dvv_Ncm-1_v2 (1) --------------------------------------------------

Dvv_Ncm-1_v1 (1) --------------------------------------------------

51 100

Dvv_Ncm_ORF (51) TTACCCTGTCTCTAGATCTTGATCGTCGTTTGGAAGACTTTTCAAATACC

Dvv_Ncm_Region_2 (1) --------------------------------------------------

Dvv_Ncm_Region_1 (1) --------------------------------------------------

Dvv_Ncm-1_v2 (1) --------------------------------------------------

Dvv_Ncm-1_v1 (1) --------------------------------------------------

101 150

Dvv_Ncm_ORF (101) CTTTTATCGTTTTTTAATTGCTTAATATCATCTTCATATCTGCTCTTCAT

Dvv_Ncm_Region_2 (1) --------------------------------------------------

Dvv_Ncm_Region_1 (1) --------------------------------------------------

Dvv_Ncm-1_v2 (1) --------------------------------------------------

Dvv_Ncm-1_v1 (1) --------------------------------------------------

151 200

Dvv_Ncm_ORF (151) CCATTCGTAGTCTTTCTTCCTCTTGTTATCTTCTTTAGAATATTTTTTGG

Dvv_Ncm_Region_2 (1) --------------------------------------------------

Dvv_Ncm_Region_1 (1) --------------------------------------------------

Dvv_Ncm-1_v2 (1) --------------------------------------------------

Dvv_Ncm-1_v1 (1) --------------------------------------------------

201 250

Dvv_Ncm_ORF (201) ACGAGGATCTATCGGGATTTTTGTCAGATTTATGTCTATCTTCCTTTCTA

Dvv_Ncm_Region_2 (1) --------------------------------------------------

Dvv_Ncm_Region_1 (1) --------------------------------------------------

Dvv_Ncm-1_v2 (1) --------------------------------------------------

Dvv_Ncm-1_v1 (1) --------------------------------------------------

251 300

Dvv_Ncm_ORF (251) TGTTTGTCTCTAGGTTTGTCTGCGTGTTCTTTCTCTTTTGATCTAGGTCT

Dvv_Ncm_Region_2 (1) --------------------------------------------------

Dvv_Ncm_Region_1 (1) --------------------------------------------------

Dvv_Ncm-1_v2 (1) --------------------------------------------------

Dvv_Ncm-1_v1 (1) --------------------------------------------------

301 350

Dvv_Ncm_ORF (301) AGACTTAGAATTTACTTTCGAATTTTTTTCTTCTAGCTTTTTGGTTTTTT

Dvv_Ncm_Region_2 (1) --------------------------------------------------

Dvv_Ncm_Region_1 (1) --------------------------------------------------

Dvv_Ncm-1_v2 (1) --------------------------------------------------

Dvv_Ncm-1_v1 (1) --------------------------------------------------

351 400

Dvv_Ncm_ORF (351) TCTTCTTTTTGCCTCTATCGTCTTCAGAACTTGATGAATCTTCAGAGCTG

Dvv_Ncm_Region_2 (1) --------------------------------------------------

Dvv_Ncm_Region_1 (1) --------------------------------------------------

Dvv_Ncm-1_v2 (1) --------------------------------------------------

Dvv_Ncm-1_v1 (1) --------------------------------------------------

401 450

Dvv_Ncm_ORF (401) TCGTCACTACTATCTTCCTCAGAACTACTTTCTGAACTACTACTACCACT

Dvv_Ncm_Region_2 (1) --------------------------------------------------

Dvv_Ncm_Region_1 (1) --------------------------------------------------

Dvv_Ncm-1_v2 (1) --------------------------------------------------

Dvv_Ncm-1_v1 (1) --------------------------------------------------

451 500

Dvv_Ncm_ORF (451) GCTTTCCTTTTCTTTAGTGGCTAACTTCATTTCCATCATTTTTGGAATAT

Dvv_Ncm_Region_2 (1) --------------------------------------------------

Dvv_Ncm_Region_1 (1) --------------------------------------------------

Dvv_Ncm-1_v2 (1) --------------------------------------------------

Dvv_Ncm-1_v1 (1) --------------------------------------------------

501 550

Dvv_Ncm_ORF (501) TTTTTAAATGTTCTCTGAGTTCATCAGTTAATCCTCCCAAACCGATAGAG

Dvv_Ncm_Region_2 (1) --------------------------------------------------

Dvv_Ncm_Region_1 (1) --------------------------------------------------

Dvv_Ncm-1_v2 (1) --------------------------------------------------

Dvv_Ncm-1_v1 (1) --------------------------------------------------

551 600

Dvv_Ncm_ORF (551) GTAAAAAAATTAATAGAAAATCTGGTATTCTTTGGGTTATCTCTAGGAAA

Dvv_Ncm_Region_2 (1) --------------------------------------------------

Dvv_Ncm_Region_1 (1) --------------------------------------------------

Dvv_Ncm-1_v2 (1) --------------------------------------------------

Dvv_Ncm-1_v1 (1) --------------------------------------------------

601 650

Dvv_Ncm_ORF (601) CAGTCCTGAAAAATAAGCCTGCAGGGTCTCATCCTTTAGCCTTGCGTTTA

Dvv_Ncm_Region_2 (1) --------------------------------------------------

Dvv_Ncm_Region_1 (1) --------------------------------------------------

Dvv_Ncm-1_v2 (1) --------------------------------------------------

Dvv_Ncm-1_v1 (1) --------------------------------------------------

651 700

Dvv_Ncm_ORF (651) ATTTTCCTAGTCCCATATATTCAGCCAATTCTTGAAACAAGATTTTTATG

Dvv_Ncm_Region_2 (1) --------------------------------------------------

Dvv_Ncm_Region_1 (1) --------------------------------------------------

Dvv_Ncm-1_v2 (1) --------------------------------------------------

Dvv_Ncm-1_v1 (1) --------------------------------------------------

701 750

Dvv_Ncm_ORF (701) AAAATCCTACTAGAACTATTGGTATCCTCTTCATTCAATTTCATGATGTC

Dvv_Ncm_Region_2 (1) --------------------------------------------------

Dvv_Ncm_Region_1 (1) --------------------------------------------------

Dvv_Ncm-1_v2 (1) --------------------------------------------------

Dvv_Ncm-1_v1 (1) --------------------------------------------------

751 800

Dvv_Ncm_ORF (751) AAGGACTTCCCATCCAATGGCATCCGTAAAAAGTAAATGCGCAAAAAATT

Dvv_Ncm_Region_2 (1) --------------------------------------------------

Dvv_Ncm_Region_1 (1) --------------------------------------------------

Dvv_Ncm-1_v2 (1) --------------------------------------------------

Dvv_Ncm-1_v1 (1) --------------------------------------------------

801 850

Dvv_Ncm_ORF (801) TGCTGACGTTTCTTAACCTGTTAGCATCTAGTCTGTGAGTGGTAGAATAG

Dvv_Ncm_Region_2 (1) -------------------GTTAGCATCTAGTCTGTGAGTGGTAGAATAG

Dvv_Ncm_Region_1 (1) --------------------------------------------------

Dvv_Ncm-1_v2 (1) --------------------------------------------------

Dvv_Ncm-1_v1 (1) --------------------------------------------------

851 900

Dvv_Ncm_ORF (851) GTATCTTTAAAAATTTGTTGGAAAGGCTCGATATACACTTTGTTGATTTG

Dvv_Ncm_Region_2 (32) GTATCTTTAAAAATTTGTTGGAAAGGCTCGATATACACTTTGTTGATTTG

Dvv_Ncm_Region_1 (1) --------------------------------------------------

Dvv_Ncm-1_v2 (1) --------------------------------------------------

Dvv_Ncm-1_v1 (1) --------------------------------------------------

901 950

Dvv_Ncm_ORF (901) ACAAAATCTTTGAGCCAAAAGACCATAAAACTTTTCGTAGGTTCTTTGTT

Dvv_Ncm_Region_2 (82) ACAAAATCTTTGAGCCAAAAGACCATAAAACTTTTCGTAGGTTCTTTGTT

Dvv_Ncm_Region_1 (1) --------------------------------------------------

Dvv_Ncm-1_v2 (1) --------------------------------------------------

Dvv_Ncm-1_v1 (1) --------------------------------------------------

951 1000

Dvv_Ncm_ORF (951) CTGCGCAGCAGTCAAGAAACATGTGACACAATTCTATTTCTTGTCCAGGT

Dvv_Ncm_Region_2 (132) CTGCGCAGCAGTCAAGAAACATGTGACACAATTCTATTTCTTGTCCAGGT

Dvv_Ncm_Region_1 (1) --------------------------------------------------

Dvv_Ncm-1_v2 (1) --------------------------------------------------

Dvv_Ncm-1_v1 (1) --------------------------------------------------

1001 1050

Dvv_Ncm_ORF (1001) TTCAACTCCATCTTCAGTAGCTTATGTGCACATTCTTCAAAATCTAAACT

Dvv_Ncm_Region_2 (182) TTCAACTCCATCTTCAGTAGCTTATGTGCACATTCTTCAAAATCTAAACT

Dvv_Ncm_Region_1 (1) --------------------------------------------------

Dvv_Ncm-1_v2 (1) --------------------------------------------------

Dvv_Ncm-1_v1 (1) --------------------------------------------------

1051 1100

Dvv_Ncm_ORF (1051) AGACTGAATAGTCAAATATATGGTTCTTCTAAGAGAAATTAAATTCGTTT

Dvv_Ncm_Region_2 (232) AGACTGAATAGTCAAATATATGGTTCTTCTAAGAGAAATTAAATTCGTTT

Dvv_Ncm_Region_1 (1) --------------------------------------------------

Dvv_Ncm-1_v2 (1) --------------------------------------------------

Dvv_Ncm-1_v1 (1) --------------------------------------------------

1101 1150

Dvv_Ncm_ORF (1101) CAGTATTGTCAATAATTGTTCCCTGGTCCTTGACTTCGTCCTCATTTTCA

Dvv_Ncm_Region_2 (282) CAGTATTGTCAATAATTGTTCCCTGGTCCTTGACTTCGTCCTCATTTTCA

Dvv_Ncm_Region_1 (1) --------------------------------------------------

Dvv_Ncm-1_v2 (1) --------------------------------------------------

Dvv_Ncm-1_v1 (1) --------------------------------------------------

1151 1200

Dvv_Ncm_ORF (1151) TTATCAGATTCCTCTTCTGAACCTTCGGAACCTGATTCAGACTCACCATC

Dvv_Ncm_Region_2 (332) TTATCAGATTCCTCTTCTGAACCTTCGGAACCTGATTCAGACTCACCATC

Dvv_Ncm_Region_1 (1) --------------------------------------------------

Dvv_Ncm-1_v2 (1) --------------------------------------------------

Dvv_Ncm-1_v1 (1) --------------------------------------------------

1201 1250

Dvv_Ncm_ORF (1201) ACTGCCTAAGATTTCTTTACTAAGGGTTTTGTATTTATCTTCATTTTCTT

Dvv_Ncm_Region_2 (382) ACTGCCTAAGATTTCTTTACTAAGGG------------------------

Dvv_Ncm_Region_1 (1) --------------------------------------------------

Dvv_Ncm-1_v2 (1) --------------------------------------------------

Dvv_Ncm-1_v1 (1) --------------------------------------------------

1251 1300

Dvv_Ncm_ORF (1251) CATAACTCTCATCAAATTTGAACACATTCAATATATCCTCTGCATCAGCC

Dvv_Ncm_Region_2 (408) --------------------------------------------------

Dvv_Ncm_Region_1 (1) --------------------------------------------------

Dvv_Ncm-1_v2 (1) --------------------------------------------------

Dvv_Ncm-1_v1 (1) --------------------------------------------------

1301 1350

Dvv_Ncm_ORF (1301) TCTTTAACATCATCTAACATAATAAGATGAGTGAATTGATCTTCCTCTTC

Dvv_Ncm_Region_2 (408) --------------------------------------------------

Dvv_Ncm_Region_1 (1) --------------------------------------------------

Dvv_Ncm-1_v2 (1) --------------------------------------------------

Dvv_Ncm-1_v1 (1) --------------------------------------------------

1351 1400

Dvv_Ncm_ORF (1351) TACTAAATCTAATTCTTCTACGACAGCAGCATGATCCTTAAATCCGTCTT

Dvv_Ncm_Region_2 (408) --------------------------------------------------

Dvv_Ncm_Region_1 (1) --------------------------------------------------

Dvv_Ncm-1_v2 (1) --------------------------------------------------

Dvv_Ncm-1_v1 (1) --------------------------------------------------

1401 1450

Dvv_Ncm_ORF (1401) TCCTTATTTGAAACATAACTTCATCATGTACTGAATTCTTTTTTTCTAGC

Dvv_Ncm_Region_2 (408) --------------------------------------------------

Dvv_Ncm_Region_1 (1) --------------------------------------------------

Dvv_Ncm-1_v2 (1) --------------------------------------------------

Dvv_Ncm-1_v1 (1) --------------------------------------------------

1451 1500

Dvv_Ncm_ORF (1451) TGGCCTTCATGTAAAATGTTTCTTAACATCTCAAATATAGCAGTAATACC

Dvv_Ncm_Region_2 (408) --------------------------------------------------

Dvv_Ncm_Region_1 (1) --------------------------------------------------

Dvv_Ncm-1_v2 (1) --------------------------------------------------

Dvv_Ncm-1_v1 (1) --------------------------------------------------

1501 1550

Dvv_Ncm_ORF (1501) TCTACTTGAAACTTCTGTCAGTTTTTGTCCACATTCCTTCAAAAATGAAA

Dvv_Ncm_Region_2 (408) --------------------------------------------------

Dvv_Ncm_Region_1 (1) --------------------------------------------------

Dvv_Ncm-1_v2 (1) --------------------------------------------------

Dvv_Ncm-1_v1 (1) --------------------------------------------------

1551 1600

Dvv_Ncm_ORF (1551) TGGCCACCTCCACAGAATCATCTGTAGGAGTCTCCACCAACAATGTAAGT

Dvv_Ncm_Region_2 (408) --------------------------------------------------

Dvv_Ncm_Region_1 (1) --------------------------------------------------

Dvv_Ncm-1_v2 (1) --------------------------------------------------

Dvv_Ncm-1_v1 (1) --------------------------------------------------

1601 1650

Dvv_Ncm_ORF (1601) ATCTCCAAAGCCAAAATTTCATGTGCCACTCTCTGATTTACTAAATGAGC

Dvv_Ncm_Region_2 (408) --------------------------------------------------

Dvv_Ncm_Region_1 (1) --------------------------------------------------

Dvv_Ncm-1_v2 (1) --------------------------------------------------

Dvv_Ncm-1_v1 (1) --------------------------------------------------

1651 1700

Dvv_Ncm_ORF (1651) TACGAAAGTAGTAGCCGATATGCAAATAGACTTATTATTTTGTTTAAACC

Dvv_Ncm_Region_2 (408) --------------------------------------------------

Dvv_Ncm_Region_1 (1) --------------------------------------------------

Dvv_Ncm-1_v2 (1) --------------------------------------------------

Dvv_Ncm-1_v1 (1) --------------------------------------------------

1701 1750

Dvv_Ncm_ORF (1701) CTCTTTTGAACTGCAAAACCAACCTCTTCAATAAAAGCTCTCCTATACTT

Dvv_Ncm_Region_2 (408) --------------------------------------------------

Dvv_Ncm_Region_1 (1) --------------------------------------------------

Dvv_Ncm-1_v2 (1) --------------------------------------------------

Dvv_Ncm-1_v1 (1) --------------------------------------------------

1751 1800

Dvv_Ncm_ORF (1751) GGAAACTTCGAATTAATAACAGCTACTAAGGCTGCGTAAACGTTTGTAAA

Dvv_Ncm_Region_2 (408) --------------------------------------------------

Dvv_Ncm_Region_1 (1) -----------------------------GGCTGCGTAAACGTTTGTAAA

Dvv_Ncm-1_v2 (1) -----------------------------GGCTGCGTAAACGTTTGTAAA

Dvv_Ncm-1_v1 (1) --------------------------------------------------

1801 1850

Dvv_Ncm_ORF (1801) AGTAGGAGATGCTGCTTGTGCTTGTATTATTGACTTGCACAGCAAACCTC

Dvv_Ncm_Region_2 (408) --------------------------------------------------

Dvv_Ncm_Region_1 (22) AGTAGGAGATGCTGCTTGTGCTTGTATTATTGACTTGCACAGCAAACCTC

Dvv_Ncm-1_v2 (22) AGTAGGAGATGCTGCTTGTGCTTGTATTATTGACTTGCACAGCAAACCTC

Dvv_Ncm-1_v1 (1) --------------------------------------------------

1851 1900

Dvv_Ncm_ORF (1851) TACCTCTTACTATATTTTCATGCAATAATTCTCTGGCGATGATGCCAATA

Dvv_Ncm_Region_2 (408) --------------------------------------------------

Dvv_Ncm_Region_1 (72) TACCTCTTACTATATTTTCATGCAATAATTCTCTGGCGATGATGCCAATA

Dvv_Ncm-1_v2 (72) TACCTCTTACTATATTTTCATGCAATAATTCTCTGGCGATGATGCCAAT-

Dvv_Ncm-1_v1 (1) --------------------------------------------------

1901 1950

Dvv_Ncm_ORF (1901) TTCGAGGTGTTAATTTTATTAATGTAACCATGAACGGATTTCTTTAAGGC

Dvv_Ncm_Region_2 (408) --------------------------------------------------

Dvv_Ncm_Region_1 (122) TTCGAGGTGTTAATTTTATTAATGTAACCATGAACGGATTTCTTTAAGGC

Dvv_Ncm-1_v2 (121) --------------------------------------------------

Dvv_Ncm-1_v1 (1) ------------------TTAATGTAACCATGAACGGATTTCTTTAAGGC

1951 2000

Dvv_Ncm_ORF (1951) TTCCCATGCTATACGCTGATAGGCTGCTGATGTTTTGTCTGTAATACTGG

Dvv_Ncm_Region_2 (408) --------------------------------------------------

Dvv_Ncm_Region_1 (172) TTCCCATGCTATACGCTGATAGGCTGCTGATGTTTTGTCTGTAATACTGG

Dvv_Ncm-1_v2 (121) --------------------------------------------------

Dvv_Ncm-1_v1 (33) TTCCCATGCTATACGCTGATAGGCTGCTGATGTTTTGTCTGTAATACTGG

2001 2050

Dvv_Ncm_ORF (2001) CTTGTAACAATCGTAGCTTAGCTGGGGGAATATAAGCACCACCAGTCCTC

Dvv_Ncm_Region_2 (408) --------------------------------------------------

Dvv_Ncm_Region_1 (222) CTTGTAACAATCGTAGCTTAGCTGGGGGAATATAAGCACCACCAGTCCTC

Dvv_Ncm-1_v2 (121) --------------------------------------------------

Dvv_Ncm-1_v1 (83) CTTGTAACAATCGTAGCTTAGCTGGGGGAATATAAGCACCACCAGTCCTC

2051 2100

Dvv_Ncm_ORF (2051) GATGTTAACATATCTACAGTTTTTCTTTCTCTTGGTTTGATGACTGATTT

Dvv_Ncm_Region_2 (408) --------------------------------------------------

Dvv_Ncm_Region_1 (272) GATGTTAACATATCTACAGTTTTTCTTTCTCTTGGTTTGATGACTGATTT

Dvv_Ncm-1_v2 (121) --------------------------------------------------

Dvv_Ncm-1_v1 (133) GATGTTAACATATCTACAGTTTTTCTTTCTCTTGGTTTGATGACTG----

2101 2150

Dvv_Ncm_ORF (2101) ATCAGGAACAGACTCCATTGGCTTTGGTAACTCCTTCTCCTCTGAATTTC

Dvv_Ncm_Region_2 (408) --------------------------------------------------

Dvv_Ncm_Region_1 (322) ATCAGGAACAGACTCCATTGGCTTTGGTAACTCCTTCTCCTCTGAATTTC

Dvv_Ncm-1_v2 (121) --------------------------------------------------

Dvv_Ncm-1_v1 (179) --------------------------------------------------

2151 2200

Dvv_Ncm_ORF (2151) TATCTGGCCCCTTCTTGCCAGATTCTTCGGGAGCCGCATCATAGTATCTT

Dvv_Ncm_Region_2 (408) --------------------------------------------------

Dvv_Ncm_Region_1 (372) TATCTGGCCCCTTCTTGCCAGATTCTTCGGGAGCCGCATC----------

Dvv_Ncm-1_v2 (121) --------------------------------------------------

Dvv_Ncm-1_v1 (179) --------------------------------------------------

2201 2250

Dvv_Ncm_ORF (2201) TGGCCAACTTTGGTAGTATCTTTCCTTGGATATTTATTCCAATATTTGTC

Dvv_Ncm_Region_2 (408) --------------------------------------------------

Dvv_Ncm_Region_1 (412) --------------------------------------------------

Dvv_Ncm-1_v2 (121) --------------------------------------------------

Dvv_Ncm-1_v1 (179) --------------------------------------------------

2251 2300

Dvv_Ncm_ORF (2251) ACTGTTTGGGCGATCTTCCCCATAGTATCTCTGATAATCTTTAGGGTTGT

Dvv_Ncm_Region_2 (408) --------------------------------------------------

Dvv_Ncm_Region_1 (412) --------------------------------------------------

Dvv_Ncm-1_v2 (121) --------------------------------------------------

Dvv_Ncm-1_v1 (179) --------------------------------------------------

2301 2350

Dvv_Ncm_ORF (2301) AACCTTCCTCTGATGAATCTCTGTCTCTACTATTGTGGGCTTTCTTTTTG

Dvv_Ncm_Region_2 (408) --------------------------------------------------

Dvv_Ncm_Region_1 (412) --------------------------------------------------

Dvv_Ncm-1_v2 (121) --------------------------------------------------

Dvv_Ncm-1_v1 (179) --------------------------------------------------

2351 2400

Dvv_Ncm_ORF (2351) GAAGACTTTTTCTCTTTTCGTTCTGGAGATTTAGATCTACTCTTTCTTCG

Dvv_Ncm_Region_2 (408) --------------------------------------------------

Dvv_Ncm_Region_1 (412) --------------------------------------------------

Dvv_Ncm-1_v2 (121) --------------------------------------------------

Dvv_Ncm-1_v1 (179) --------------------------------------------------

2401 2450

Dvv_Ncm_ORF (2401) TCTTTTACGTTCAGGAGAACTCAAATTAGCATCCTTGGTATCCTTGGCAT

Dvv_Ncm_Region_2 (408) --------------------------------------------------

Dvv_Ncm_Region_1 (412) --------------------------------------------------

Dvv_Ncm-1_v2 (121) --------------------------------------------------

Dvv_Ncm-1_v1 (179) --------------------------------------------------

2451 2466

Dvv_Ncm_ORF (2451) CCTTGGTATCTGGCAT

Dvv_Ncm_Region_2 (408) ----------------

Dvv_Ncm_Region_1 (412) ----------------

Dvv_Ncm-1_v2 (121) ----------------

Dvv_Ncm-1_v1 (179) ----------------

**Sequence 5:** Rpb7 Open Reading Frame (ORF), Rpb7 Region 1, and Rpb7 Region 1 v1 alignment. Blue sequence is region red sequence is version.

1 50

Dvv_Rpb7_ORF (1) ATGTTTTACCACATATCTCTAGAACACGAAATCCTACTACATCCACAATA

Dvv_Rpb7_Region_1 (1) --------------------------------------------------

Dvv_Rpb7-1_v1 (1) --------------------------------------------------

51 100

Dvv_Rpb7_ORF (51) TTTCGGACCACAACTGTTAGAAAAAGTCAAAACTAAACTGTATACCGAAG

Dvv_Rpb7_Region_1 (1) -------CCACAACTGTTAGAAAAAGTCAAAACTAAACTGTATACCGAAG

Dvv_Rpb7-1_v1 (1) --------------------------------------------------

101 150

Dvv_Rpb7_ORF (101) TTGAAGGAACTTGCACAGGAAAGTATGGATTTGTGATTGCAGTAACCACT

Dvv_Rpb7_Region_1 (44) TTGAAGGAACTTGCACAGGAAAGTATGGATTTGTGATTGCAGTAACCACT

Dvv_Rpb7-1_v1 (1) --------------------------------------------------

151 200

Dvv_Rpb7_ORF (151) ATAGATAGCATAGGTGCCGGTTTGATACTACCCGGACAAGGCTTTGTAGT

Dvv_Rpb7_Region_1 (94) ATAGATAGCATAGGTGCCGGTTTGATACTACCCGGACAAGGCTTTGTAGT

Dvv_Rpb7-1_v1 (1) --------------------------------------------------

201 250

Dvv_Rpb7_ORF (201) CTACCCGGTGAAATATAAAGCCATTGTGTTCCGTCCATTCAAAGGTGAAG

Dvv_Rpb7_Region_1 (144) CTACCCGGTGAAATATAAAGCCATTGTGTTCCGTCCATTCAAAGGTGAAG

Dvv_Rpb7-1_v1 (1) ----------------AAAGCCATTGTGTTCCGTCCATTCAAAGGTGAAG

251 300

Dvv_Rpb7_ORF (251) TCCTGGATGCGGTGGTTCGACAAGTCAACAAAGTTGGCATGTTCGCCGAA

Dvv_Rpb7_Region_1 (194) TCCTGGATGCGGTGGTTCGACAAGTCAACAAAGTTGGCATGTTCGCCGAA

Dvv_Rpb7-1_v1 (35) TCCTGGATGCGGTGGTTCGACAAGTCAACAAAGTTGGCATGTTCGCCGAA

301 350

Dvv_Rpb7_ORF (301) ATAGGTCCTTTATCTTGTTTCATTTCTCATCATTCCATACCCGCAGAAAT

Dvv_Rpb7_Region_1 (244) ATAGGTCCTTTATCTTGTTTCATTTCTCATCATTCCATACCCGCAGAAAT

Dvv_Rpb7-1_v1 (85) ATAGGTCCTTTATCTTGTTTCATTTCTCATCATTCCATACCCGCAGAAAT

351 400

Dvv_Rpb7_ORF (351) GGAGTTTTGTCCTAACGTTAATCCCCAATGCTATAAGACTAAAGACGAAG

Dvv_Rpb7_Region_1 (294) GGAGTTTTGTCCTAACGTTAATCCCCAATGCTATAAGACTAAAGACGAAG

Dvv_Rpb7-1_v1 (135) GGAGTTTTGTCCTAACGTTAATCCCCAATGCTATAAGACTAAAGACGAAG

401 450

Dvv_Rpb7_ORF (401) ATGTTGTGATACGAGCAGAAGGAGAAATCAGATTGAAAATAGTGGGTACG

Dvv_Rpb7_Region_1 (344) ATGTTGTGATACGAGCAGAAGGAGAAATCAGATTGAAAATAGTGGGT---

Dvv_Rpb7-1_v1 (185) ATGTTGTGATACGAGCAGAAGGAGAAATCAGATTGA--------------

451 500

Dvv_Rpb7_ORF (451) AGAGTAGACGCCTCAGGGATATTTGCCATTGGAACCTTAATGGATGATTA

Dvv_Rpb7_Region_1 (391) --------------------------------------------------

Dvv_Rpb7-1_v1 (221) --------------------------------------------------

501 522

Dvv_Rpb7_ORF (501) TCTGGGATTAATAAGTAATTAA

Dvv_Rpb7_Region_1 (391) ----------------------

Dvv_Rpb7-1_v1 (221) ----------------------

**Sequence 6:** CG34184 homolog Open Reading Frame (ORF), CG34184 Region 2, and CG34184 region 2 v1 and v2 alignments. Blue sequence is region red sequences ae version.

1 50

Dvv_CG34184_ORF (1) GTGGCAGATGGTTACGTGACTAGATTTACTGGTGATTTAGTGATGCTAAA

Dvv_CG34184_Region_2 (1) --------------------------------------------------

Dvv_CG34184-2_v1 (1) --------------------------------------------------

Dvv_CG34184-2_v2 (1) --------------------------------------------------

51 100

Dvv_CG34184_ORF (51) GTTATTGTGGGTATGGATAATTTTGCTAACAGAATATGAAGTATATGTTA

Dvv_CG34184_Region_2 (1) --------------------------------------------------

Dvv_CG34184-2_v1 (1) --------------------------------------------------

Dvv_CG34184-2_v2 (1) --------------------------------------------------

101 150

Dvv_CG34184_ORF (101) GTTCTTTGGAAGTCCTTAATCCGTACTTCCAATATCCAGGGGGCTCAGAT

Dvv_CG34184_Region_2 (1) --------------------------TTCCAATATCCAGGGGGCTCAGAT

Dvv_CG34184-2_v1 (1) --------------------------TTCCAATATCCAGGGGGCTCAGAT

Dvv_CG34184-2_v2 (1) --------------------------------------------------

151 200

Dvv_CG34184_ORF (151) CCTTTTATGGGGATTTTAGTAACGGTGGCAGTTCCTGTTGAAGCCGGAAC

Dvv_CG34184_Region_2 (25) CCTTTTATGGGGATTTTAGTAACGGTGGCAGTTCCTGTTGAAGCCGGAAC

Dvv_CG34184-2_v1 (25) CCTTTTATGGGGATTTTAGTAACGGTGGCAGTTCCTGTTGAAGCCGGAAC

Dvv_CG34184-2_v2 (1) --------------------------------------------------

201 250

Dvv_CG34184_ORF (201) TCCTGGAGAAGTTTTACTGTCAACCCTTTTGGAAGCCAACTATGTGATTC

Dvv_CG34184_Region_2 (75) TCCTGGAGAAGTTTTACTGTCAACCCTTTTGGAAGCCAACTATGTGATTC

Dvv_CG34184-2_v1 (75) TCCTGGAGAAGTTTTACTGTCAACCCTTTTGGAAGCCAACTATGTGATTC

Dvv_CG34184-2_v2 (1) --------------------------------------------------

251 300

Dvv_CG34184_ORF (251) CAAGTAACCAAACTCGGTTTACCTGGCCACCCGATTACCCTGCAACTACT

Dvv_CG34184_Region_2 (125) CAAGTAACCAAACTCGGTTTACCTGGCCACCCGATTACCCTGCAACTACT

Dvv_CG34184-2_v1 (125) CAAGTAACCAAACTCGGTTTACCTGGCCACCCGATTACCCTGCAACTACT

Dvv_CG34184-2_v2 (1) --------------------------------------------------

301 350

Dvv_CG34184_ORF (301) TCAGAAAGGATGTTTTTATACAATTTATTTAAAAGAAAAATTGAAGACTT

Dvv_CG34184_Region_2 (175) TCAGAAAGGATGTTTTTATACAATTTATTTAAAAGAAAAATTGAAGACTT

Dvv_CG34184-2_v1 (175) TCAGAAAGGATGTTTTTATACAAT--------------------------

Dvv_CG34184-2_v2 (1) --------------------------------------------------

351 400

Dvv_CG34184_ORF (351) TGGGTTCCCTGGGCAAAGTTGTCTGCTAAGGGCAATATGTGAATCAGCAC

Dvv_CG34184_Region_2 (225) TGGGTTCCCTGGGCAAAGTTGTCTGCTAAGGGCAATATGTGAATCAGCAC

Dvv_CG34184-2_v1 (199) --------------------------------------------------

Dvv_CG34184-2_v2 (1) --------------------------------------------------

401 450

Dvv_CG34184_ORF (401) AAATGAGCAGCCAGCATACAGGTCTCCTTGGGGATATTCTTCATATCCTA

Dvv_CG34184_Region_2 (275) AAATGAGCAGCCAGCATACAGGTCTCCTTGGGGATATTCTTCATATCCTA

Dvv_CG34184-2_v1 (199) --------------------------------------------------

Dvv_CG34184-2_v2 (1) ---------------------GTCTCCTTGGGGATATTCTTCATATCCTA

451 500

Dvv_CG34184_ORF (451) TTAACACCATCTAGTTCCAAAATGGAGGAGCAGCTTGTTGAATACGAAGA

Dvv_CG34184_Region_2 (325) TTAACACCATCTAGTTCCAAAATGGAGGAGCAGCTTGTTGAATACGAAGA

Dvv_CG34184-2_v1 (199) --------------------------------------------------

Dvv_CG34184-2_v2 (30) TTAACACCATCTAGTTCCAAAATGGAGGAGCAGCTTGTTGAATACGAAGA

501 550

Dvv_CG34184_ORF (501) AGCTGAACGCCAAGGAAAAGAAAACACATGTGAAAAATACTATAAGAAAT

Dvv_CG34184_Region_2 (375) AGCTGAACGCCAAGGAAAAGAAAACACATGTGAAAAATACTATAAGAAAT

Dvv_CG34184-2_v1 (199) --------------------------------------------------

Dvv_CG34184-2_v2 (80) AGCTGAACGCCAAGGAAAAGAAAACACATGTGAAAAATACTATAAGAAAT

551 600

Dvv_CG34184_ORF (551) GCCCCCATAGCATATTAGACTCCATTACCCGAGTCACTAACATAGTAGAT

Dvv_CG34184_Region_2 (425) GCCCCCATAGCATATTAGACTCC---------------------------

Dvv_CG34184-2_v1 (199) --------------------------------------------------

Dvv_CG34184-2_v2 (130) GCCCCCATAGCATATTAGACTCC---------------------------

601 648

Dvv_CG34184_ORF (601) TATGAAGCTACCAAATATTTTTCAAAAAATATTGTCAAACTATTTTAA

Dvv_CG34184_Region_2 (448) ------------------------------------------------

Dvv_CG34184-2_v1 (199) ------------------------------------------------

Dvv_CG34184-2_v2 (153) ------------------------------------------------

**Sequence 7:** Amino acid alignment and % identity matrix of Dre4 from *T. castaneum, D. v. virgifera, and M. aeneus.*


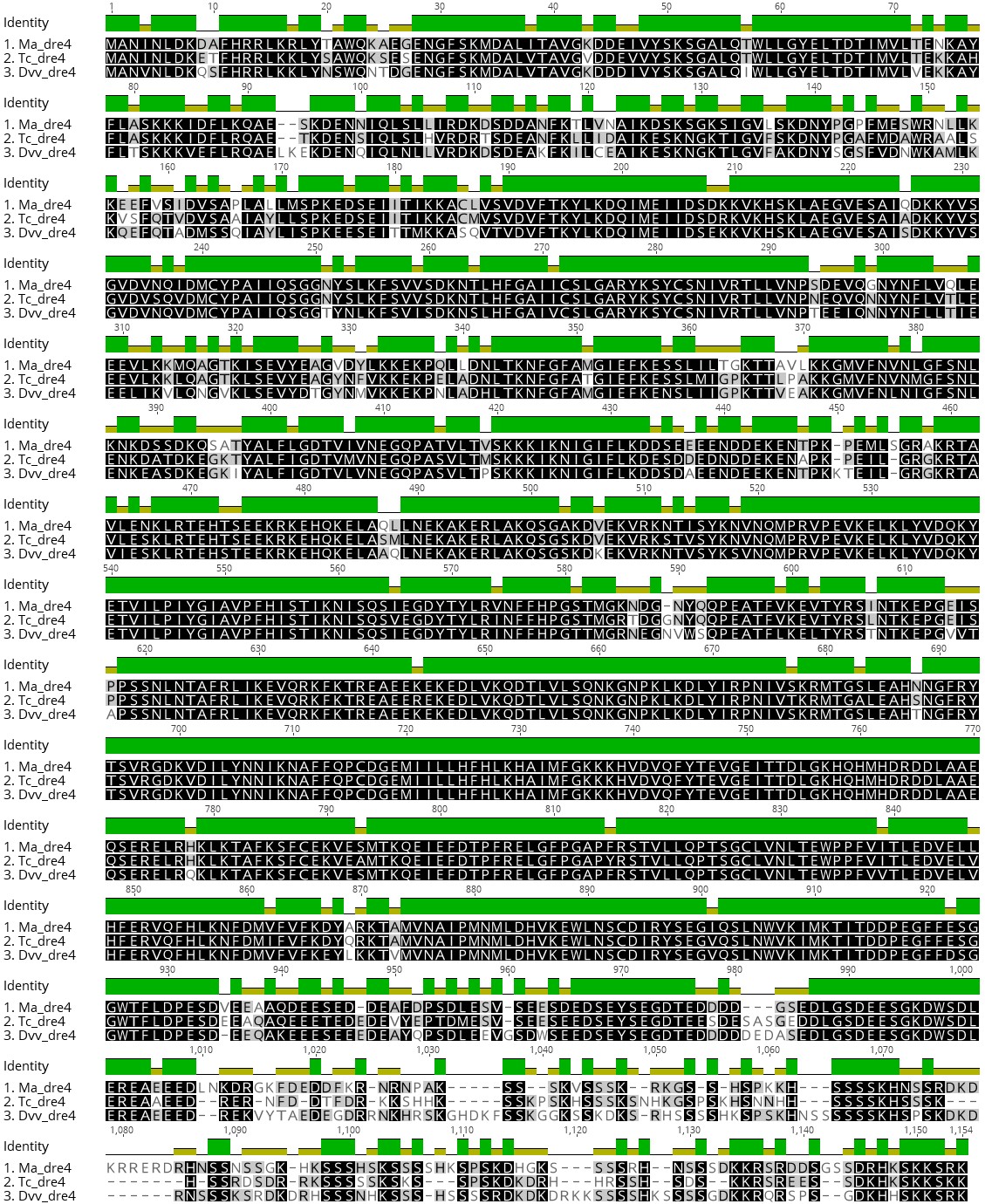


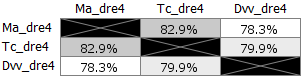


**Sequence 8:** Amino acid alignment and % identity matrix of RpII140 from *T. castaneum, D. v. virgifera, and M. aeneus.*


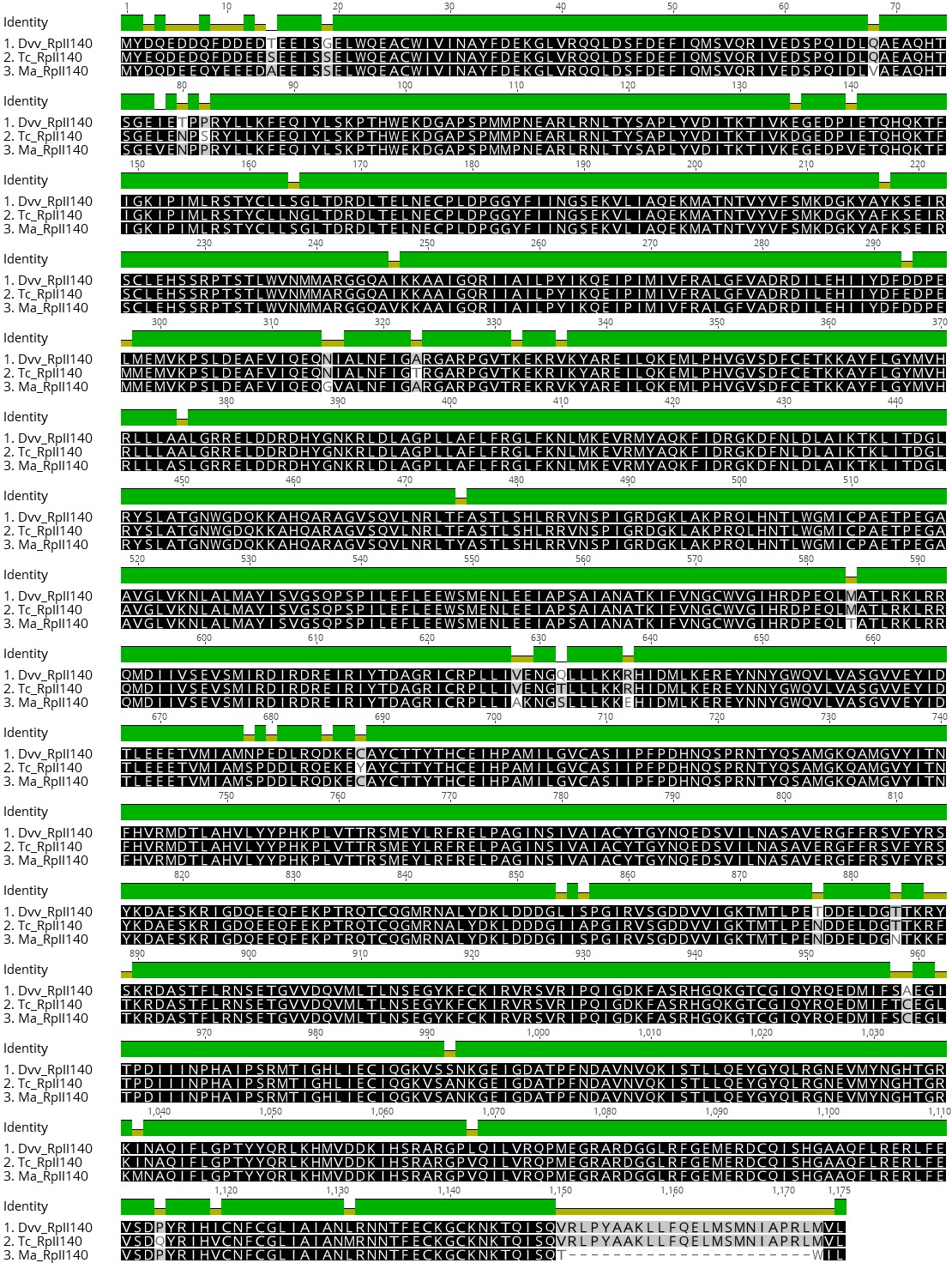


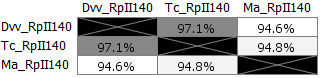


**Sequence 9:** Amino acid alignment and % identity matrix of Rop from *T.castaneum, D.v.virgifera, and M.aeneus*


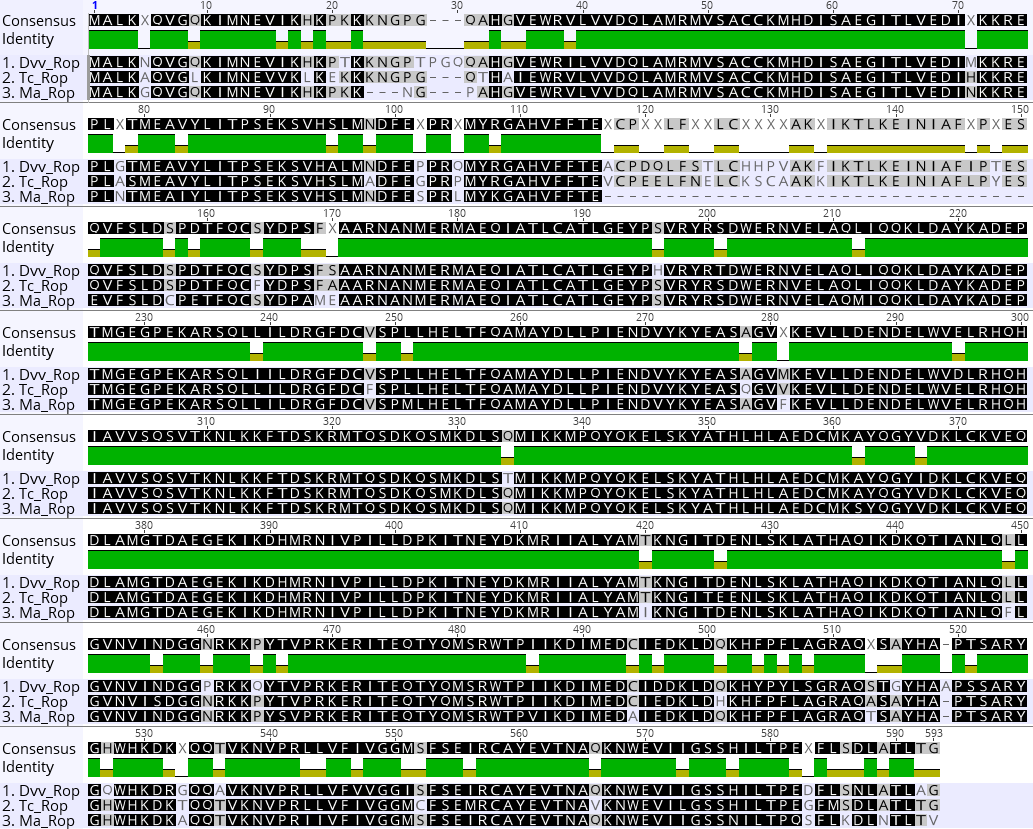


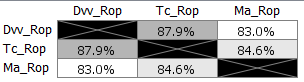


**Sequence 10:** Nucleotide alignment, performed with ClustalW, and % identity matrix of *Rop* from *T. castaneum, D. v. virgifera, and M. aeneus.*
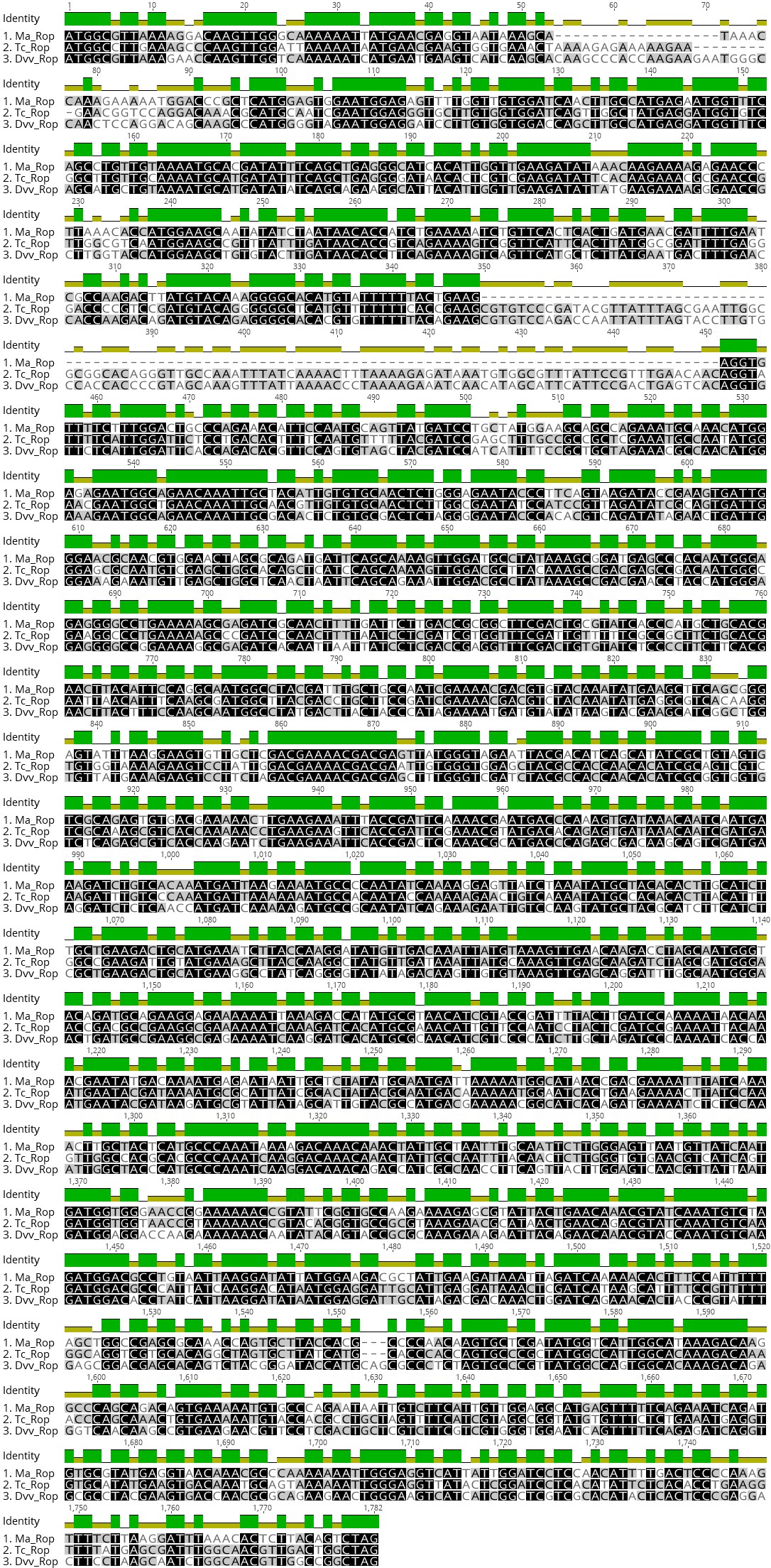


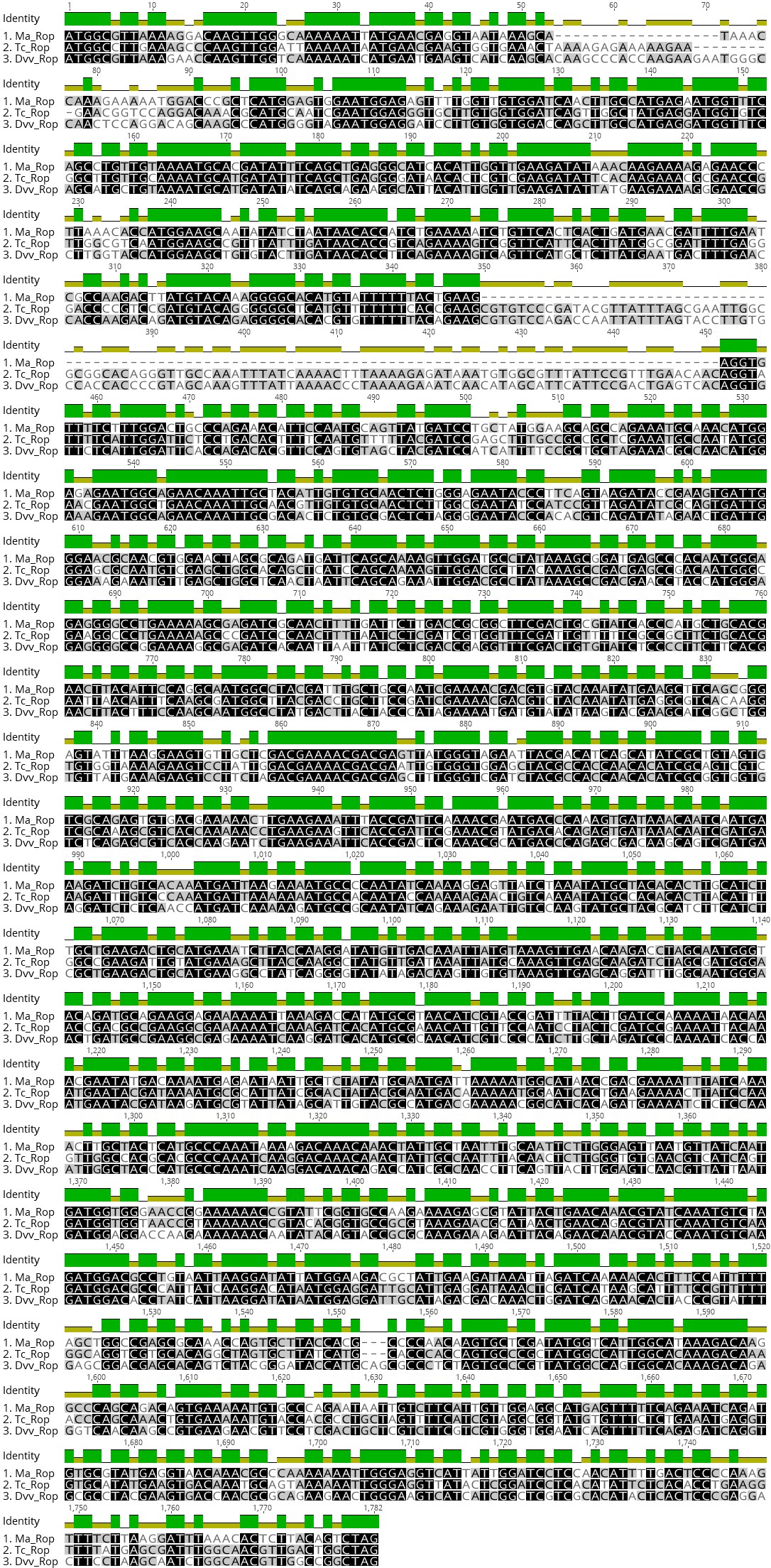

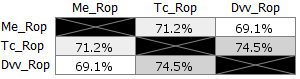


**Sequence 11:** Nucleotide alignment, performed with ClustalW, and % identity matrix of *dre4* from *T. castaneum, D. v. virgifera, and M. aeneus.* **
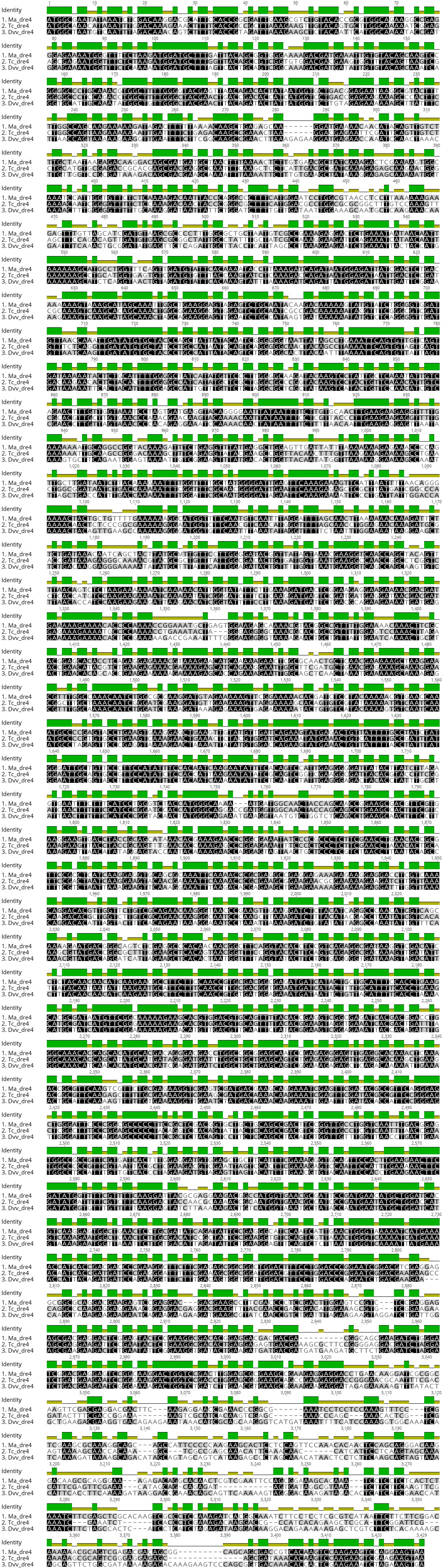
**

**
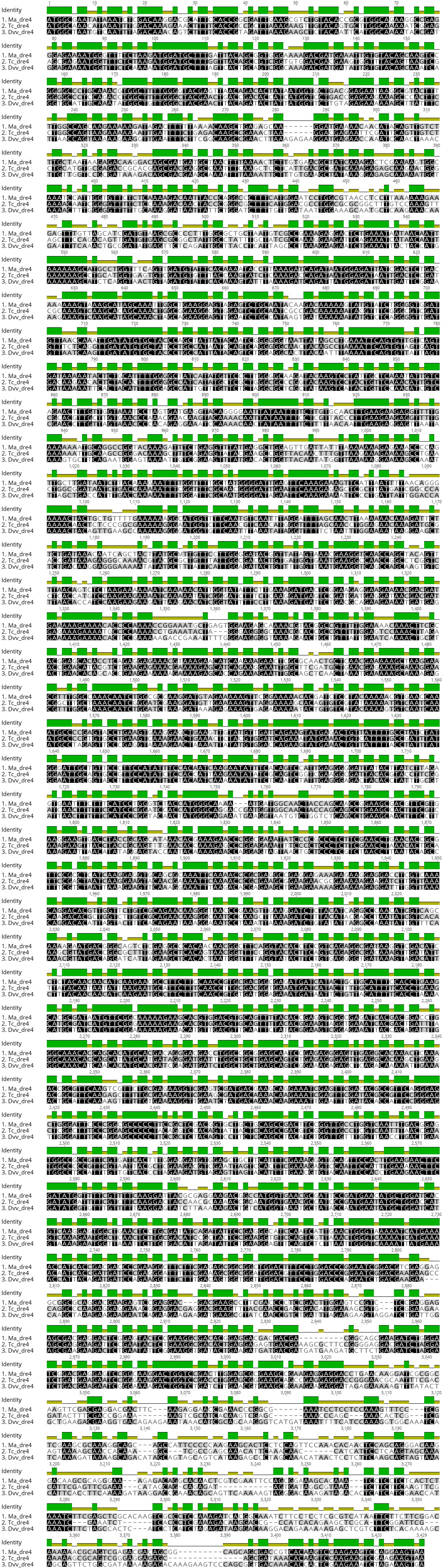
**

**
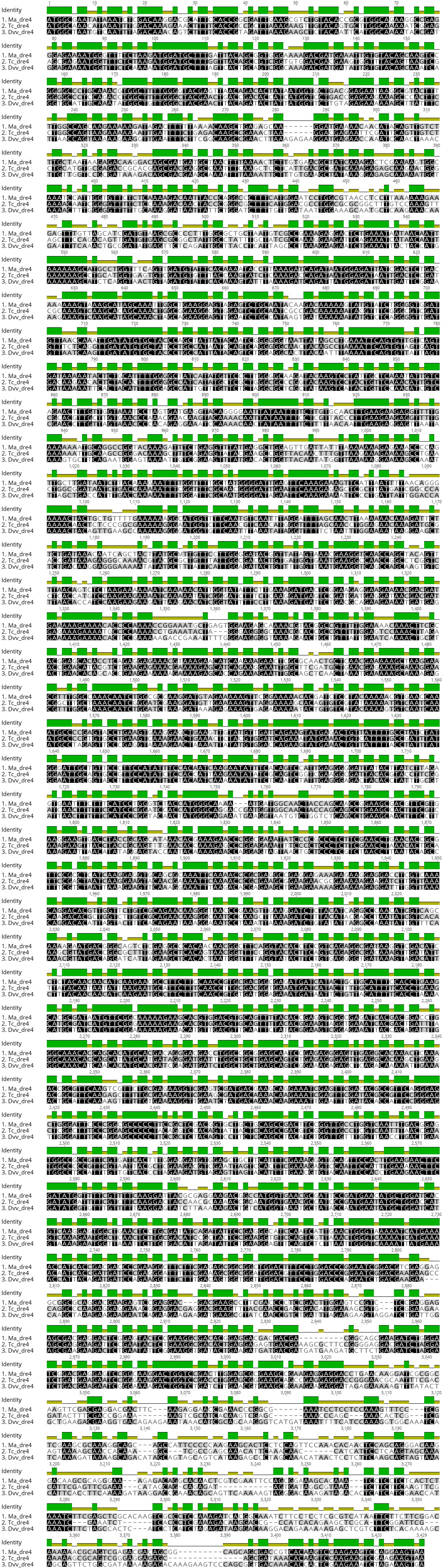
**


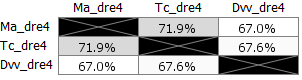


**Sequence 12:** Nucleotide alignment, performed with ClustalW, and % identity matrix of *RpII140* from *T. castaneum, D. v. virgifera, and M. aeneus.*


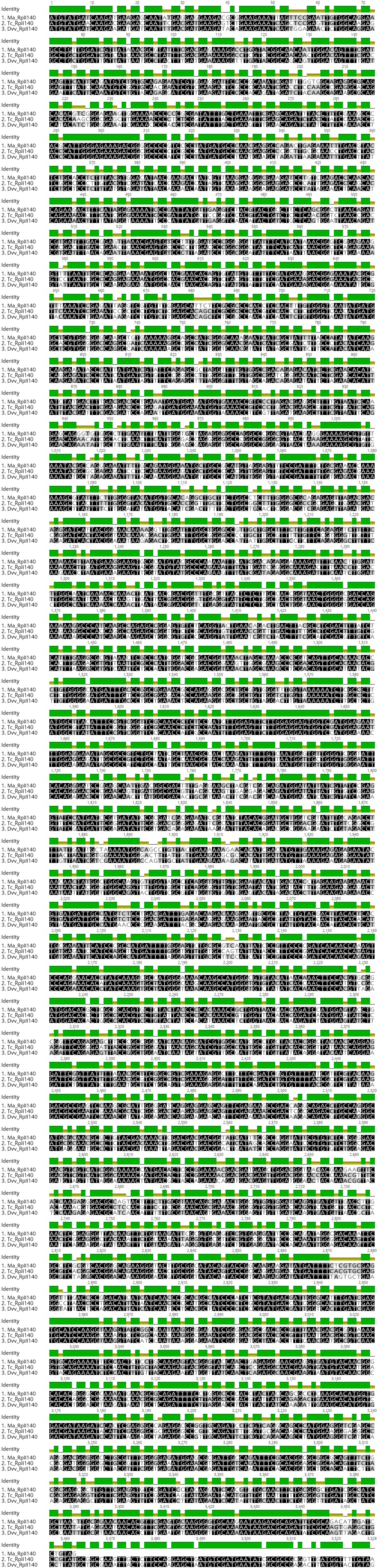


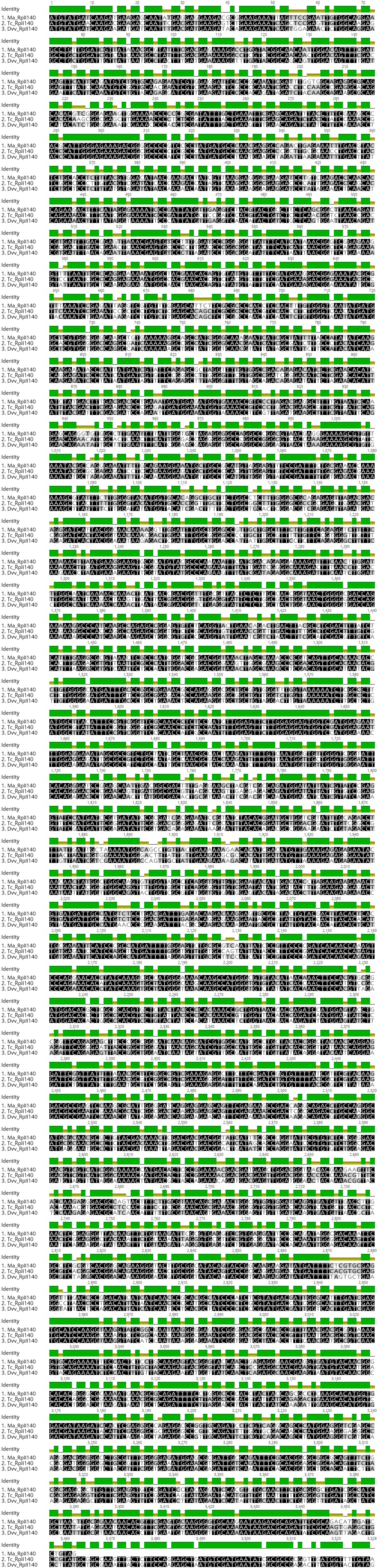


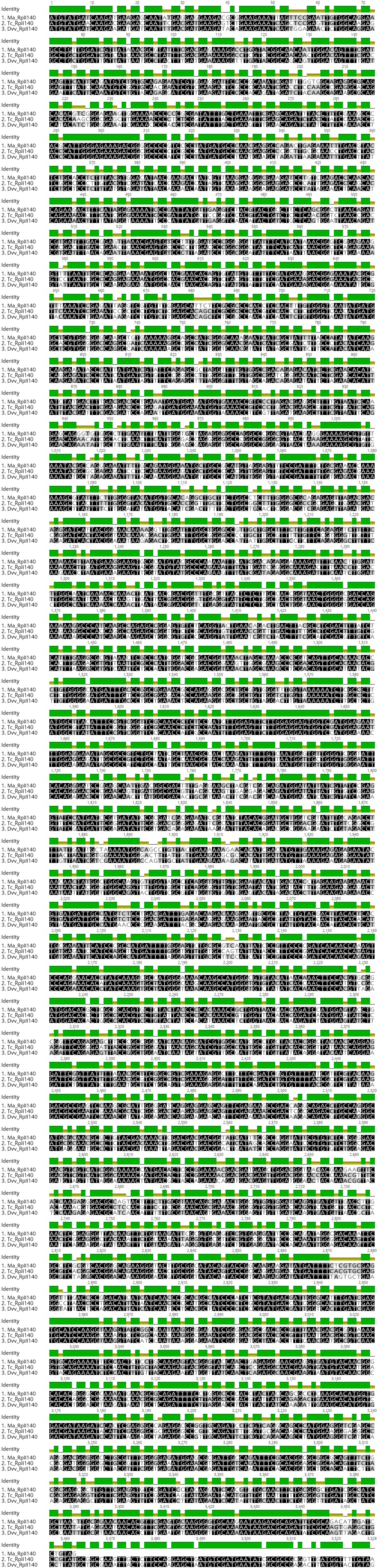


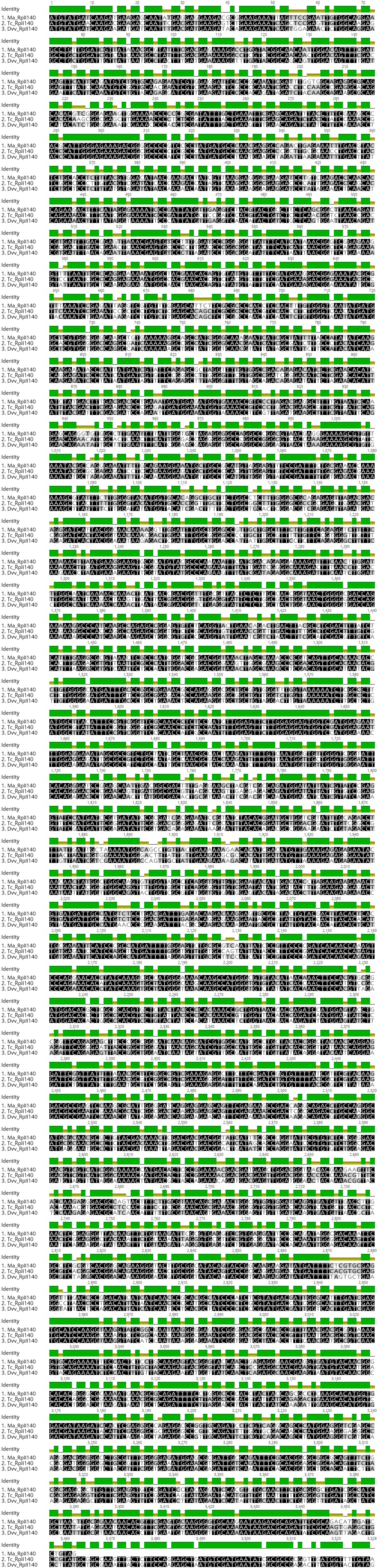


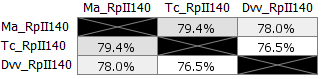

Supplement: Supplementary file 2 — Supplementary Figures and Materials [file 41598_2018_20416_MOESM2_ESM.docx]
